# (2‐Ethylhexyl)sodium: A Hexane‐Soluble Reagent for Br/Na‐Exchanges and Directed Metalations in Continuous Flow

**DOI:** 10.1002/anie.202103031

**Published:** 2021-05-03

**Authors:** Johannes H. Harenberg, Niels Weidmann, Alexander J. Wiegand, Carla A. Hoefer, Rajasekar Reddy Annapureddy, Paul Knochel

**Affiliations:** ^1^ Department Chemie Ludwig-Maximilians-Universität München Butenandtstrasse 5–13, Haus F 81377 München Germany

**Keywords:** Br/Na-exchange, directed sodiation, flow chemistry, packed-bed reactor, sodium

## Abstract

We report the on‐demand generation of hexane‐soluble (2‐ethylhexyl)sodium (**1**) from 3‐(chloromethyl)heptane (**2**) using a sodium‐packed‐bed reactor under continuous flow conditions. Thus, the resulting solution of **1** is free of elemental sodium and therefore suited for a range of synthetic applications. This new procedure avoids the storage of an alkylsodium and limits the handling of metallic sodium to a minimum. (2‐Ethylhexyl)sodium (**1**) proved to be a very useful reagent and undergoes in‐line Br/Na‐exchanges as well as directed sodiations. The resulting arylsodium intermediates are subsequently trapped in batch with various electrophiles such as ketones, aldehydes, Weinreb‐amides, imines, allyl bromides, disulfides and alkyl iodides. A reaction scale‐up of the Br/Na‐exchange using an in‐line electrophile quench was also reported.

## Graphical abstract

A generation of sodium metal free, hexane‐soluble (2‐ethylhexyl)sodium using a sodium‐packed‐bed reactor in a continuous flow set‐up was reported. (2‐Ethylhexyl)sodium proved to be a very useful sodium organometallic reagent and was used for in‐line sodiations as well as Br/Na‐exchange reactions. The arylsodiums were trapped with various electrophiles. A reaction scale‐up of the Br/Na‐exchange using an in‐line electrophile quench was reported.

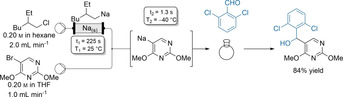

Organosodium reagents are highly reactive organometallics towards various electrophiles due to the very ionic character of the C−Na bond.^[1]^ Despite the appealing chemical properties and the low price, high abundancy and low toxicity of sodium, these compounds have seldomly found applications in organic syntheses.^[2]^ Dimethylethylamine soluble NaDA (sodium diisopropylamide) was prepared by Collum and co‐workers as an alternative to the frequently used LDA (lithium diisopropylamide).^[3]^ Recently, Asako and Takai have reported a new method for the preparation of arylsodiums via a Br/Na‐exchange using neopentylsodium, which was prepared by the reaction of neopentyl chloride with sodium dispersion (Scheme 1 a). This procedure seems to limit the trapping of the resulting arylsodium to R_3_SiCl, D_2_O and transmetalation reactions.^[4]^ The presence of residual sodium dispersion may hamper the use of more complex electrophiles. In contrast to well established lithium chemistry,^[5]^ the use of organosodium reagents remains underexploited in continuous flow due to their poor solubility.^[6]^ We have reported the generation of organosodium and ‐potassium derivatives in continuous flow using Na‐ and K‐amide bases.^[7]^ In the course of this work, we envisioned a new procedure for generating soluble alkylsodiums in continuous flow expanding pioneering work of Alcázar,^[8]^ Ley,^[9]^ McQuade^[8a]^ and others,^[10]^ which established the use of metal‐packed‐bed reactors for the direct preparation of Mg or Zn organometallics in continuous flow. Herein, we report a new sodium‐packed‐bed reactor for on‐demand generation of the hexane‐soluble sodium reagent (2‐ethylhexyl)sodium (**1**)^[11]^ from readily available 3‐(chloromethyl)heptane (**2**), which was used for performing in‐line Br/Na‐exchanges as well as directed metalations (Scheme 1 b) in continuous flow.

**Scheme 1:**
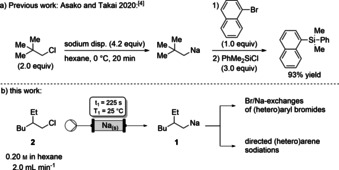
a) Generation of neopentylsodium in batch and its use in halogen/sodium‐exchange reactions. b) On‐demand continuous flow generation of (2‐ethylhexyl)sodium (**1**) and subsequent in‐line Br/Na‐exchange and directed metalation.

To prepare the packed‐bed reactor, we charged a glass column (7.5 mL) with sodium particles (3.4 mL, Ø ca. 1 mm).[^12^, ^13^] The resulting mixed‐bed reactor^[14]^ was flushed with dry hexane and was activated using a 0.1 m solution of *i*‐PrOH in hexane. Pumping alkyl chloride **2** (0.2 m in hexane, 2.0 mL min^−1^, 25 °C) through the reactor afforded a slightly yellow solution of **1** in hexane (ca. 0.15 m).^[15]^ This soluble alkylsodium species^[16]^ was free of metallic sodium and was directly used for in‐line Br/Na‐exchanges as well as directed sodiations. Collected aliquots of **1** prepared in continuous flow showed moderate stability (Figure 1), demonstrating the importance of the direct use of the sodium species. This on‐demand procedure avoids storage problems of instable **1** and considerably limits hazards of working with metallic sodium. Whereas preparation of **1** in batch led to a dark solution over metallic sodium, the flow procedure resulted in a slightly yellow solution of **1** free of elemental sodium (Figure 1).

**Figure 1:**
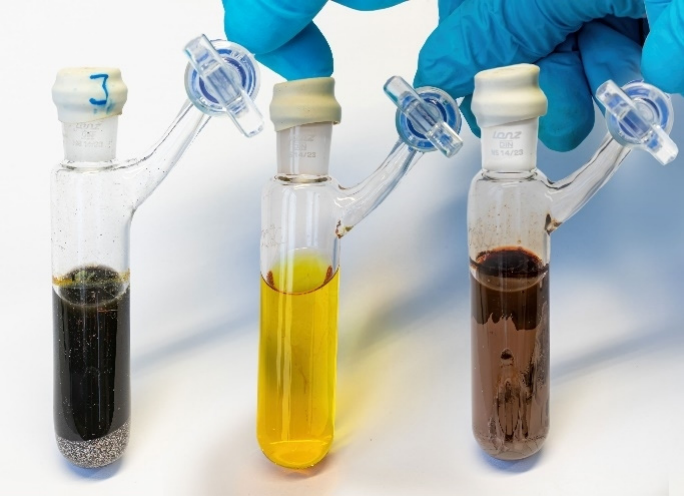
From left to right: (2‐Ethylhexyl)sodium (**1**) in hexane prepared in batch over metallic sodium, 5 min after addition of **2**. (2‐Ethylhexyl)sodium (**1**) in hexane prepared via a sodium‐packed‐bed reactor, 5 min after collecting. (2‐Ethylhexyl)sodium (**1**) in hexane prepared via a packed‐bed sodium reactor, 18 h after collecting.

The sodium‐packed‐bed reactor was used without clogging for ca. 1 h pumping a 0.2 m solution of **2** in hexane with a flow rate of 2.0 mL min^−1^. The soluble organosodium **1** was directly used for Br/Na‐exchanges with various aryl bromides of type **3**. Thus, mixing a THF‐solution of 1‐bromonaphthalene (**3 a**, 0.2 m, 1.0 mL min^−1^) with (2‐ethylhexyl)sodium (**1**, 0.2 m, 2.0 mL min^−1^) in a T‐shaped mixer gave 1‐naphthylsodium (**4 a**) (−40 °C, 1.3 s).^[17]^

Subsequent batch‐quench of **4 a** with benzophenone (**5 a**) or enolisable 2‐ethylbutyraldehyde (**5 b**) afforded the desired alcohols (**6 aa**–**6 ab**) in 70–87 % yield (Table 1). The resulting arylsodiums reacted instantly with various electrophiles such as ketones, aldehydes, Weinreb‐amides, imines, allyl bromides, disulfides and alkyl iodides. Weinreb‐amide **5 c** and imine **5 d** gave the expected products **6 bc** and **6 bd** in 65–73 % yield upon Br/Na‐exchange on 1‐bromo‐3,5‐di‐*tert*‐butylbenzene (**3 b**). Halogen‐ and trifluoromethyl‐substituted aryl bromides such as **3 c** and **3 d** furnished after batch quenching the functionalized arenes **6 cd**, **6 ce**, **6 da** and **6 df** in 62–90 % yield. Electron‐rich bromoarenes were well suited for such a Br/Na‐exchange in continuous flow affording the polyfunctionalized arenes **6 ec**, **6 eg**, **6 fh**, **6 fi**, **6 fj 6 gk**, **6 gd** and **6 gl** in 67–95 % yield.

**Table 1:** On‐demand preparation of alkylsodium reagent **1** from alkyl chloride **2** followed by Br/Na‐exchange on aryl bromides of type **3** leading to arylsodiums of type **4** and subsequent batch quench with electrophiles of type **5** leading to products of type **6**. 
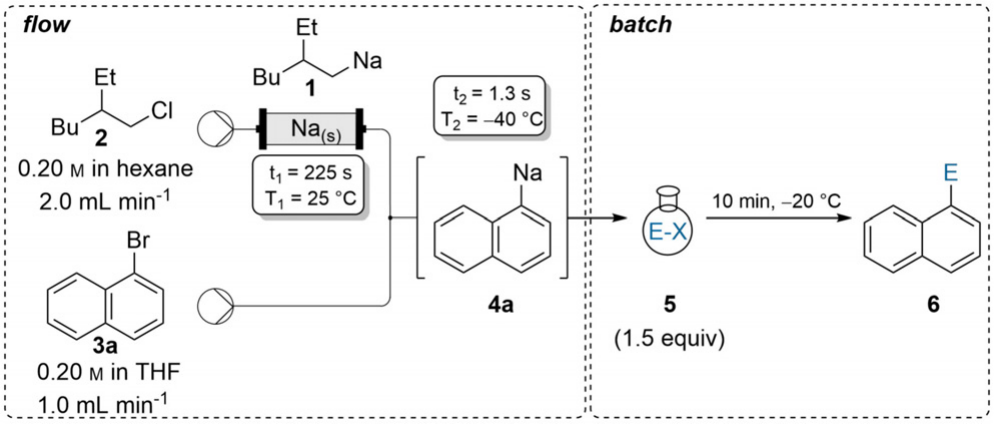

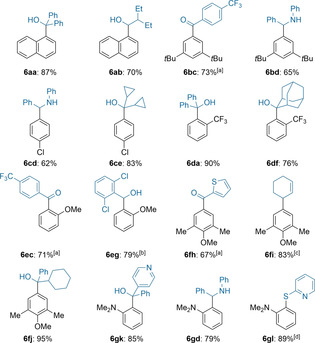

Yields of analytically pure products. [a] From the Weinreb‐amide [b] 2.0 equiv E‐X were used. [c] From the allyl bromide with addition of 50 mol % CuCN⋅2 LiCl. [d] From the disulfide.

Nitrogen containing heterocycles are important building blocks in pharmaceutical and agricultural chemistry.^[18]^ Therefore, the functionalization of those scaffolds is an ongoing task in synthetic chemistry.^[19]^ The exchange procedure was extended towards heterocyclic bromides using the optimized reaction conditions. Br/Na‐exchange on 2‐bromopyridine (**7 a**) at −40 °C using a combined flow rate of 3.0 mL min^−1^ led to the desired aryl‐sodium **8 a**, which was subsequently quenched in batch with ketones **5 a** and **5 m** affording the tertiary alcohols **9 aa** and **9 am** in 81–86 % yield (Table 2). Similarly, 5‐methyl‐2‐bromopyridine (**7 b**) and highly substituted bromopyrimidine **7 c** underwent Br/Na‐exchanges. Batch quenching using various electrophiles of type **5** led to the functionalized *N*‐heterocycles **9 bc**, **9 cg**, **9 cn**, **9 cc** and **9 cl** in 78–96 % yield. Furthermore, 2‐bromothiazole (**7 d**) was converted into the corresponding sodium species **8 d**, which was quenched with ketone **5 j** resulting in **9 dj** (66 % yield). Trapping **8 d** with a racemic mixture of α‐ionone (**5 o**) gave the 1,2‐addition product **9 do** (50 % yield, *dr* 1:1).

**Table 2:** On‐demand preparation of alkylsodium reagent **1** from alkyl chloride **2** followed by Br/Na‐exchange on heteroaryl bromides of type **7** leading to heteroarylsodiums of type **8** and subsequent batch quench with electrophiles of type **5** leading to products of type **9**. 
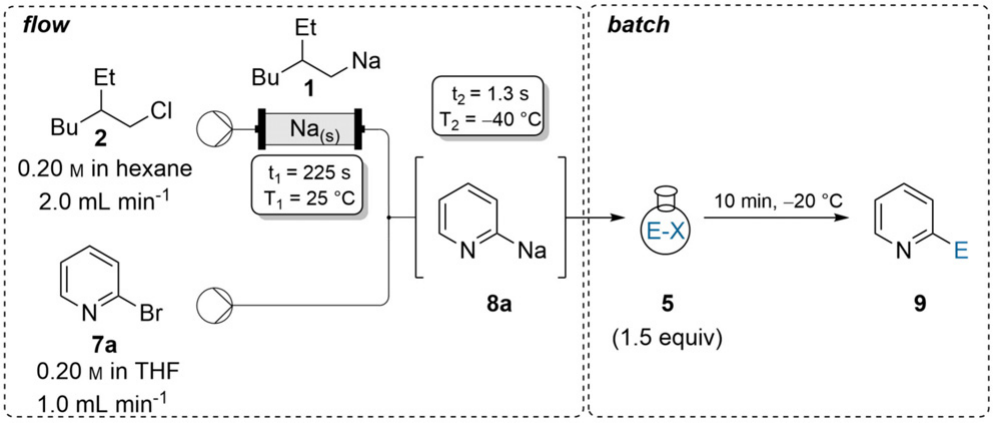

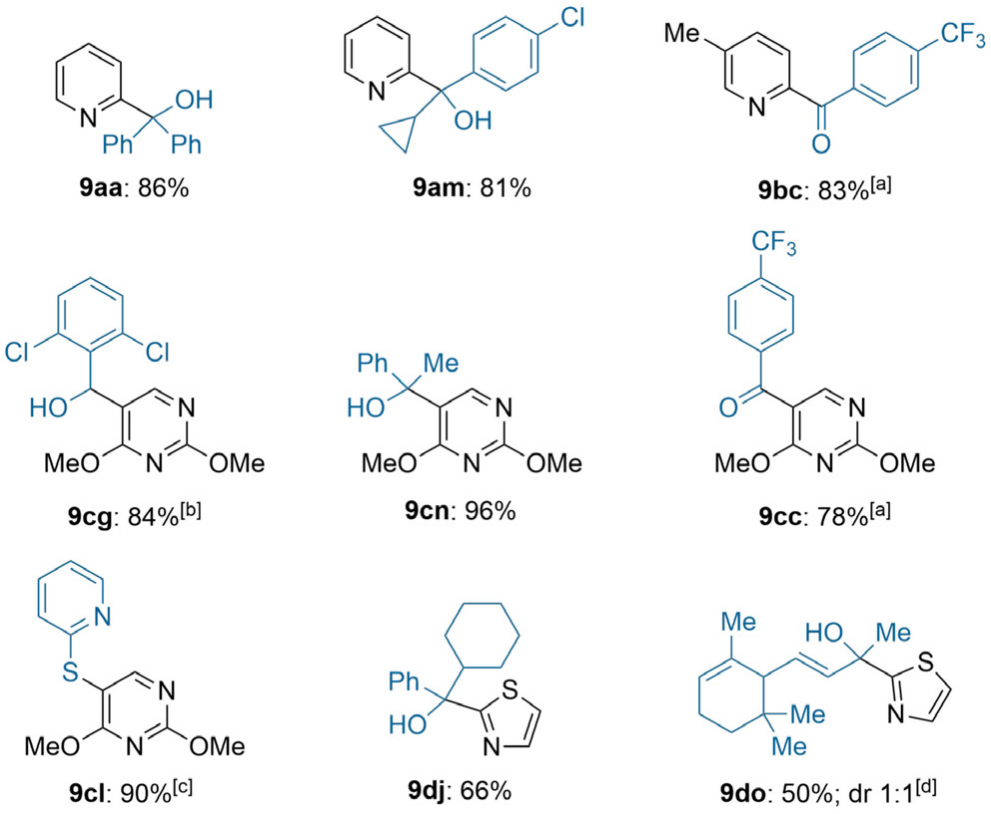

Yields of analytically pure products. [a] From the Weinreb‐amide. [b] 2.0 equiv E‐X were used. [c] From the disulfide. [d] From racemic α‐ionone.

To demonstrate the scalability^[20]^ of the Br/Na‐exchange reaction, an in‐line electrophile quench was set up. Thus, pumping a solution of **2** (0.2 m, 2.0 mL min^−1^) through the sodium‐packed reactor resulted in the sodium exchange reagent **1**. 2‐Bromopyridine (**7 a**, 0.2 m, 1.0 mL min^−1^) was mixed with the solution of **1** in a T‐shaped mixer. After passing through a micro‐reactor (0.6 s, −40 °C, combined flow rate: 3.0 mL min^−1^), the pyridylsodium **8 a** was trapped in‐line with a solution of benzophenone (**5 a**, 0.1 m, 3.0 mL min^−1^). Increasing the runtime 10‐ or 17.5‐fold (2.0 or 3.5 mmol) led to the functionalized pyridine **9 aa** in 64–65 % isolated yield (Scheme 2).

**Scheme 2:**
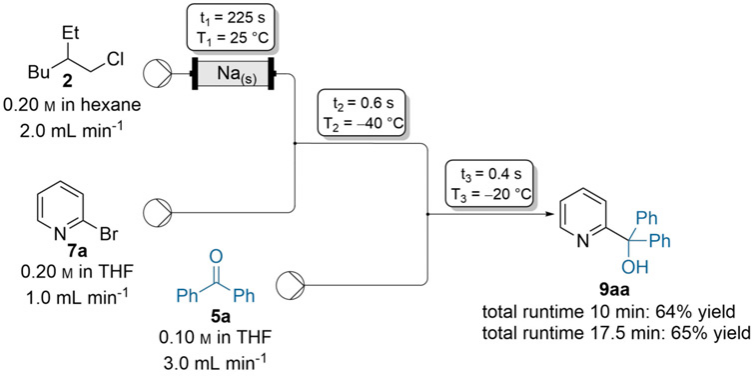
Scale‐up of the Br/Na‐exchange reaction using 2‐bromopyridine (**7 a**), (2‐ethylhexyl)sodium (**1**) as exchange reagent and benzophenone (**5 a**) as electrophile, applying in‐line quenching conditions.

Apart from halogen/lithium‐exchanges, alkyllithiums are frequently used in directed metalations converting readily available arene starting materials into highly reactive aryllithiums, therefore allowing the functionalization of previously unreactive aromatic C−H bonds.^[21]^ We expected **1** to behave similarly, and indeed without changing the set‐up of the continuous flow procedure, (2‐ethylhexyl)sodium (**1**) was able to metalate benzothiophene (**10 a**) resulting in the corresponding sodium species **11 a**.^[22]^ Quenching with carbonyl electrophiles **5 m**, **5 c**, and **5 g** gave the expected products **12 am**, **12 ac** and **12 ag** in 73–87 % yield (Table 3). Imidazole **10 b** was metalated similarly and subsequent batch quench gave the products **12 bl**, **12 bd** and **12 bf** in 55–79 % isolated yield. The electron rich 1,3‐dimethoxybenzene (**10 c**) was converted to the arylsodium **11 c**. Trapping with ketone **5 m** or disulfide **5 p** gave the desired products **12 cm** and **12 cp** in 86–88 % yield. Additionally, transition metal free Wurtz‐type coupling,^[23]^ with iodooctane (**5 q**) gave the alkylated product **12 cq** in 46 % yield.

**Table 3:** On‐demand preparation of alkylsodium reagent **1** from alkyl chloride **2** followed by directed metalation of (hetero)arenes of type **10** leading to (hetero)arylsodiums of type **11** and subsequent batch quench with electrophiles of type **5** leading to products of type **12**. 
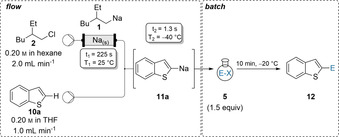

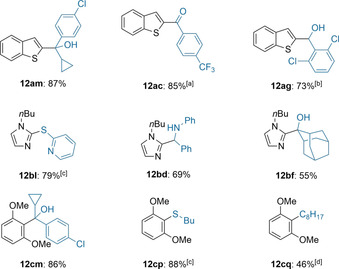

Yields of analytically pure products. [a] From the Weinreb‐amide. [b] 2.0 equiv E‐X were used [c] From the disulfide. [d] From the alkyl iodide.

In summary, we have reported the on‐demand generation of sodium metal free, hexane‐soluble (2‐ethylhexyl)sodium from 3‐(chloromethyl)heptane using a sodium‐packed‐bed reactor in a commercially available continuous flow set‐up. The procedure avoids storage of alkylsodium species and limits the handling of metallic sodium to a minimum. (2‐Ethylhexyl)sodium was used for in‐line sodiations and Br/Na‐exchange reactions. The resulting arylsodiums were subsequently trapped with various electrophiles such as ketones, aldehydes, Weinreb‐amides, imines, allyl bromides, disulfides and alkyl iodides. A reaction scale‐up of the Br/Na‐exchange using an in‐line electrophile quench was reported. Further investigations on the use of alkylsodium reagents are currently under way in our laboratories.

## Conflict of interest

The authors declare no conflict of interest.

## Supporting information

As a service to our authors and readers, this journal provides supporting information supplied by the authors. Such materials are peer reviewed and may be re‐organized for online delivery, but are not copy‐edited or typeset. Technical support issues arising from supporting information (other than missing files) should be addressed to the authors.

Supplementary
