# Supplementary material for: (2‐Ethylhexyl)sodium: A Hexane‐Soluble Reagent for Br/Na‐Exchanges and Directed Metalations in Continuous Flow
Source: Angew Chem Int Ed Engl. 2021 May 3;60(26):14296–301. doi: 10.1002/anie.202103031 (PMC8252725; doi:10.1002/anie.202103031)

Angewandte Chemie
Eine Zeitschrift der Gesellschaft Deutscher Chemiker

Supporting Information

(2-Ethylhexyl)sodium: A Hexane-Soluble Reagent for Br/Na-Exchanges and Directed Metalations in Continuous Flow

*Johannes H. Harenberg, Niels Weidmann, Alexander J. Wiegand, Carla A. Hoefer, Rajasekar Reddy Annapureddy, and Paul Knochel**

anie_202103031_sm_miscellaneous_information.pdf

Department of Chemistry, Ludwig-Maximilians-Universität Munich,
Butenandtstr. 5 – 13, 81377 Munich, Germany
E-Mail: knochel@cup.uni-muenchen.de

Table of contents

General information 2
Solvents 2
Analytical data 2
Typical procedures and screenings 3
Preparation of the products 18
NMR data 55

General information

Solvents

Hexane was continuously refluxed and freshly distilled from sodium benzophenone ketyl under nitrogen and stored over molecular sieves. Solvents for column chromatography were distilled prior to use.

Reagents

All reagents were obtained from commercial sources and used without further purification unless otherwise stated.

CuCN·2LiCl[1] solution (1.00 M in THF) was prepared by drying CuCN (8.96 g, 100 mmol) and LiCl (8.48 g, 200 mmol) in a Schlenk flask under vacuum for 5 h at 150 °C. After cooling to 25 °C, dry THF (100 mL) was added and stirred until the salts were dissolved.

Chromatography

Flash column chromatography was performed using SiO_2 60 (0.040-0.063 mm, 230-400 mesh ASTM) from Merck. Thin layer chromatography (TLC) was performed using aluminum plates covered with SiO_2 (Merck 60, F-254). Spots were visualized under UV light.

Analytical data

Yields refer to isolated yields of compounds estimated to be >95% pure as determined by ^{1}H-NMR (25 °C) and capillary GC. NMR spectra were recorded on Bruker ARX 200, AC 300, WH 400 or AMX 600 instruments. Chemical shifts are reported as δ-values in ppm relative to the deuterated solvent peak: $CDCl_3$ (δH: 7.26; δC: 77.16). For the observation of the observed signal multiplicities, the following abbreviations were used: s (singlet), d (doublet), dd (doublet of doublets), t (triplet), q (quartet), quint (quintet), sext (sextet), sept (septet) and m (multiplet). Melting points are uncorrected and were measured on a Büchi B.540 apparatus. Infrared spectra were recorded from 4000-400 cm^{-1} on a Nicolet 510 FT-IR or a Perkin-Elmer 281 IR spectrometer. Absorption bands are reported in wavenumbers (cm^{-1}). Gas chromatography (GC) was performed with instruments of the type Hewlett-Packard 6890 or 5890 Series II, using a column of the type HP 5 (Hewlett-Packard, 5% phenylmethylpolysiloxane; length: 10 m, diameter: 0.25 mm, film thickness: 0.25 µm). The detection was accomplished using a flame ionization detector. Mass spectra (MS) and high resolution mass spectra (HRMS) were recorded on a Finnigan MAT95Q or Finnigan MAT90 instrument for electron impact ionization (EI) and electrospray ionization (ESI). For the combination of gas chromatography with mass spectroscopic detection, a GC-MS of the type Hewlett-Packard 6890 / MSD 5793 networking

---

[1] P. Knochel, M. C. P. Yeh, S. C. Berk, J. Talbert, *J. Org. Chem.* **1988**, *53*, 2390-2392.

was used (column: HP 5-MS, Hewlett-Packard; 5% phenylmethylpolysiloxane; length: 15 m, diameter 0.25 mm; film thickness: 0.25 µm).

Typical procedures and screenings

General remarks on flow and subsequent batch quenching reactions

Tetradecane (n-$C_{14}H_{30}$), tridecane (n-$C_{13}H_{28}$), dodecane (n-$C_{12}H_{26}$) or undecane (n-$C_{11}H_{24}$) were used as internal standards. All flasks were heat gun dried (650 °C) under vacuum and backfilled with argon after cooling. Syringes, which were used to transfer reagents and solvents, were purged with argon three times prior to use. Batch quenching reactions were carried out with magnetic stirring. Flow reactions were performed on commercially available flow systems. A Vapourtec E-series Integrated Flow Chemistry System with 3rd Pump Kit, Organometallic Kit, Collection Valve Kit and Cryogenic Reaction Kit was used. Hexane solutions of the 3-(chloromethyl)heptane and THF solutions of the remaining reactants were kept in flasks with rubber septa under an argon atmosphere during the reactions. All reactions were performed in coiled tube reactors. Coiled reactors were made from PFA or PTFE Teflon (I.D. = 0.8 mm or 0.25 mm, O.D. = 1.6 mm) tubing and T-pieces (I.D. = 0.5 mm) were used as mixers. Prior to performing reactions, the system was dried by flushing with dry THF (flow rate: 1.00 mL/min; run-time: 10 to 30 min) or by first flushing six times with MeOH followed by dry *n*-hexane (flow rate: 1.00 mL/min; run-time: 10 to 30 min).

Typical procedure 1 (TP1): Preparation and activation of the sodium-packed-bed reactor

A 50 ml round bottom flask was charged with an oval shaped stirring bar (length: 2.5 cm; width: 1.2 cm) and sodium dispersion (30 wt% in toluene, particle size <0.1 mm, 10 mL). The sodium dispersion was stirred for 4 h at 300 to 400 rpm (Figure SI 1). An ovendried Omnifit® Labware glass column (length: 25 cm; inner diameter: 6.6 mm, figure SI 2) was closed at one side with a nonadjustable PTFE endpiece. The column was charged with the previously stirred sodium dispersion (particle size ca. 1 mm) using a 10 mL syringe without a cannula until the sodium metal reached a height of 10 cm (≙ 3.4 mL of sodium, Figure SI 3). The adjustable PTFE endpiece was used to close the column and was adjusted to give the maximum height of 22 cm (≙ V_{R1} = 7.5 mL). The packed-bed reactor was then installed in the flow setup, attaching the ETFE nut of the inlet to the tubing (V_{pre1} = 0.60 mL) connected to the pump and the nut of the outlet to the precooling loop (V_{pre2} = 0.35 mL) connected to the T-shaped mixer. The packed-bed reactor was placed upright (the inlet of the reactor facing downwards, Figure SI 4) in an *i*-PrOH bath to maintain a temperature of 25 °C. After washing with *n*-hexane (runtime: 10 min; flow rate: 2.0 mL/min), the sodium was activated by pumping a solution of *i*-PrOH (0.1 M in *n*-hexane; runtime: 2 min; flow rate: 5.0 mL/min) through the packed-bed reactor. A solution of 3-(chloromethyl)heptane (**2**, 0.2 M in *n*-hexane; flow rate: 2.0 mL/min) was pumped through the column.

After 15 min, an aliquot was taken and analyzed by GC to monitor full conversion of the 3-(chloromethyl)heptane (**2**) to the corresponding organosodium derivative and steady state.

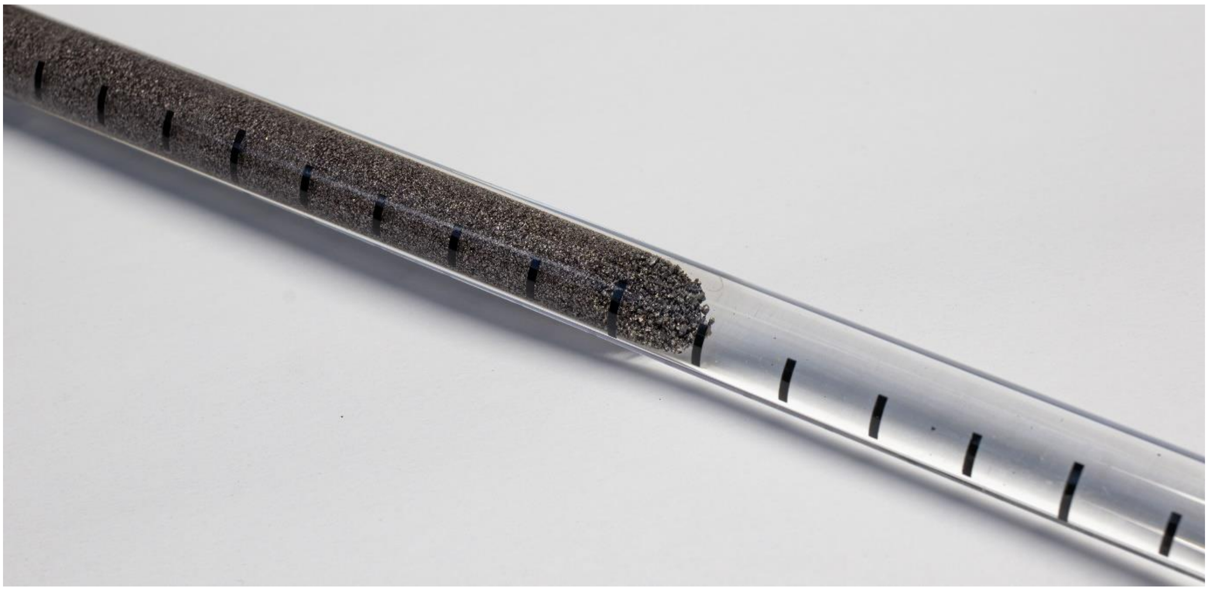

Figure SI 1: Packed-bed reactor filled with sodium particles of appropriate size.

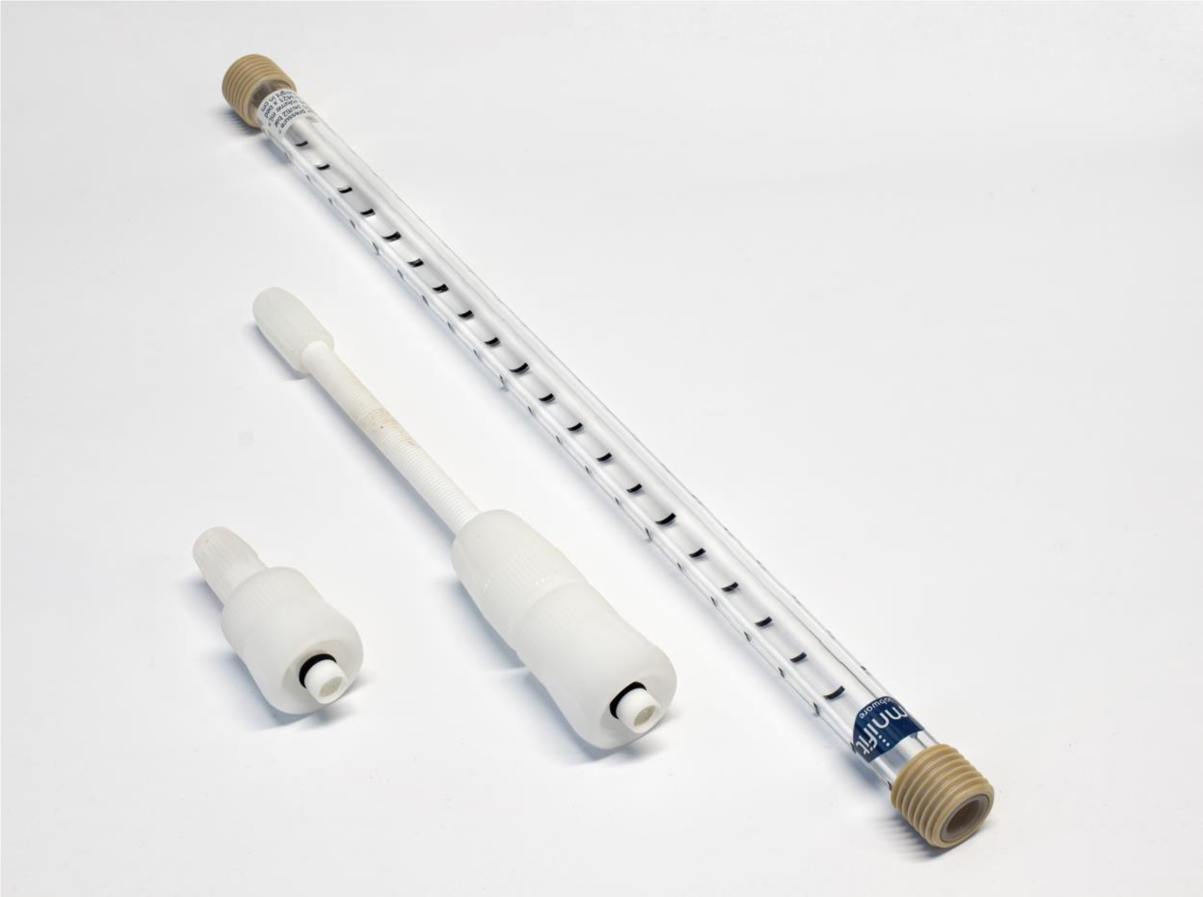

Figure SI 2: From left to right: nonadjustable PTFE endpiece with ETFE nut; adjustable PTFE endpiece with ETFE nut; Omnifit® Labware glass column.

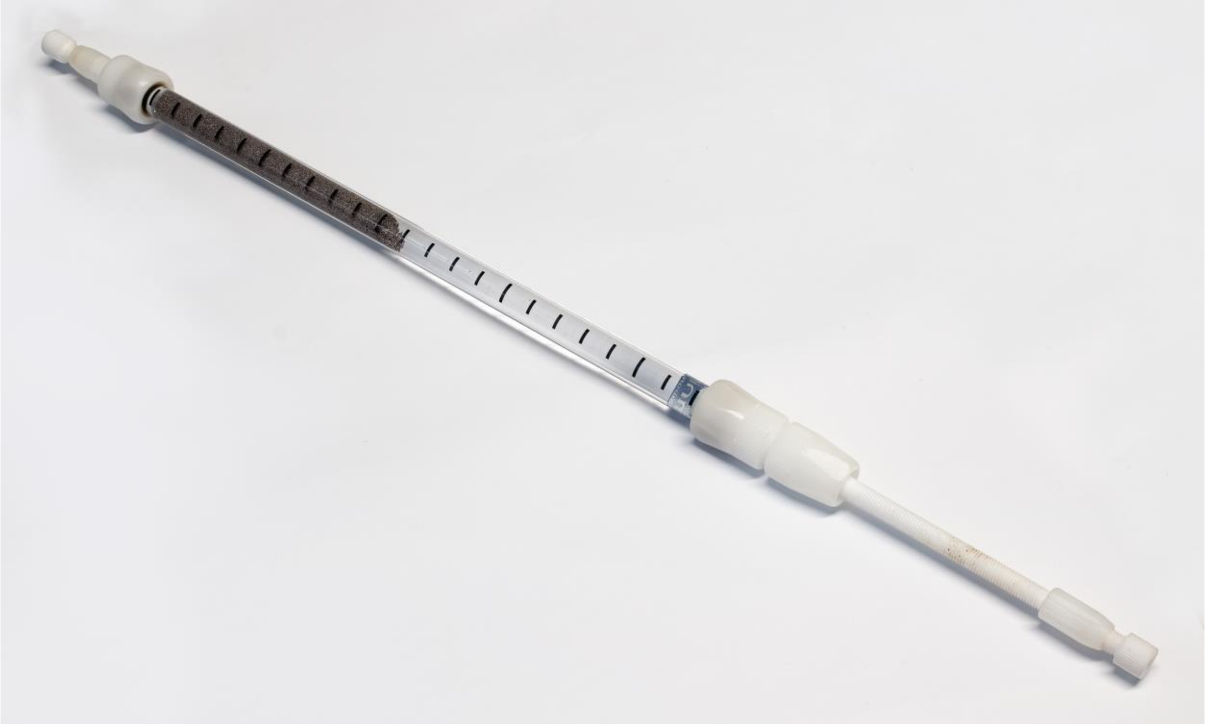

Figure SI 3: Closed packed-bed reactor filled with sodium particles. Adjustable endpiece set to give the maximum volume of V_{R1} = 7.5 mL.

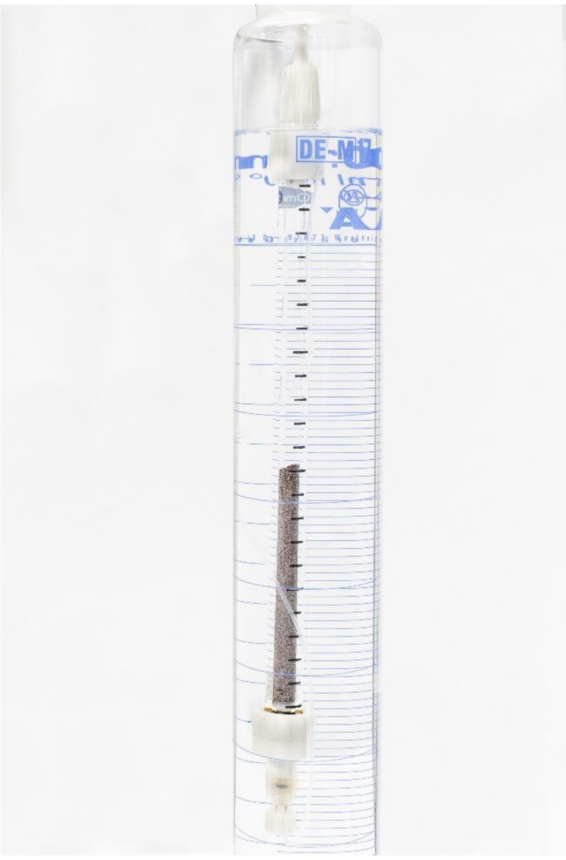

Figure SI 4: Packed-bed sodium reactor in an *i*-PrOH bath connected *via* a nonadjustable endpiece (bottom) to the tubing (V_{pre1}) attached to the pump. Adjustable endpiece (top) connected to the tubing (V_{pre2}) attached to the T-shaped mixer.

Screening of the conditions for the generation of (2-ethylhexyl)sodium using a packed-bed sodium reactor.

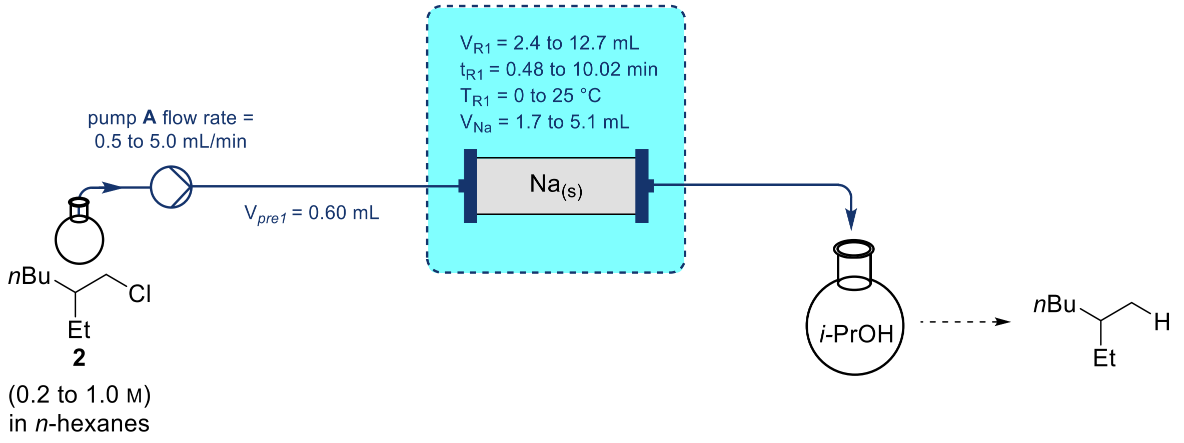

Scheme SI 1: Set-up for the optimization screening for the preparation of (2-ethylhexyl)sodium using a packed-bed sodium reactor.

Solutions of 3-(chloromethyl)heptane **2** (0.2 to 1.0 M) and tetradecane in *n*-hexane were prepared. The solution was pumped with flow rates from 0.5 to 5 mL/min through packed-bed sodium reactors (V_{R1} = 2.4 to 12.7 mL, V_{Na} = 1.7 to 5.1 mL) at various temperatures (T_{R1} = 0 to 25 °C). After passing the reactor (t_{R1} = 0.48 to 10.02 min), the reaction mixture was added to a solution of *i*-PrOH (1.1 M) in *n*-hexane. Conversion of **2** was monitored by GC-analysis.

Table SI 1: Optimization screening for the preparation of (2-ethylhexyl)sodium using a packed-bed sodium reactor.

Entry	c (**2**) [M]	V_{R1} [mL]	V (Na) [mL]	Flow rate [ml/min]	T [°C]	Conversion [%]
1	0.2	2.4	1.7	0.5	0	47
2	0.2	2.4	1.7	0.5	25 [a]	100
3	0.2	2.4	1.7	1.0	25 [a]	96
4	0.2	2.4	1.7	2.0	25 [a]	92
5	0.2	2.4	1.7	5.0	25 [a]	70
6	0.5	2.4	1.7	0.5	0	48
7	0.5	2.4	1.7	1.0	0	39
8	0.5	2.4	1.7	2.0	0	30
9	0.5	2.4	1.7	5.0	0	15
10	0.5	2.4	1.7	0.5	25 [a]	98
11	0.5	2.4	1.7	1.0	25 [a]	93
12	0.5	2.4	1.7	2.0	25 [a]	66
13	0.5	2.4	1.7	5.0	25 [a]	58
14	1.0	2.4	1.7	0.5	0	Clogging

15	1.0	2.4	1.7	0.5	25 [a]	100 [b]
16	1.0	2.4	1.7	1.0	25 [a]	81 [b]
17	1.0	2.4	1.7	2.0	25 [a]	90 [b]
18	0.2	7.5	3.4	0.5	25	100
19	0.2	7.5	3.4	1.0	25	100
20	0.2	7.5	3.4	2.0	25	100
21	0.5	7.5	3.4	0.5	25	99
22	0.5	7.5	3.4	0.5	5	82
23	0.5	7.5	3.4	1.0	5	89
24	0.5	7.5	3.4	2.0	5	62
25	0.5	7.5	3.4	5.0	5	33
26	0.5	12.7	5.1	0.5	5	100
27	0.5	12.7	5.1	1.0	5	100
28	0.5	12.7	5.1	2.0	5	100
29	0.5	12.7	5.1	5.0	5	77
30	0.5	12.7	5.1	0.5	25	100
31	0.2	12.7	5.1	2.0	25	100

[a] Without external *i*-PrOH bath (inefficient heat transfer). [b] Temperature of the packed-bed reactor increased significantly due to inefficient heat removal.

In addition to the results shown in table SI 1, it was observed that charging the entire volume of the packed-bed reactor with sodium led to clogging. Moreover, the particle size of the sodium is important. Especially, small particles led to fast clogging of the reactor, which is why the sodium dispersion was stirred for 4 h at 300 to 400 rpm (for an appropriate sodium particle size see figure SI 1).

Without any activation, the conversion of **2** remained unsatisfactory. Mechanical activation by pre-stirring the sodium in a round bottom flask did not result in high conversion rates. Best results were obtained by chemically activating the sodium by pumping a *i*-PrOH solution (0.1 M in *n*-hexane; runtime: 2 min; flow rate: 5.0 mL/min) through the packed-bed reactor. Even though, the longest column gave satisfactory results (Table SI 1 entry 26 -31), the medium sized column was used to reduce the amount of sodium used and to decrease the retention time of the generated alkyl sodium reagent **1**.

Solubility studies of (2-ethylhexyl)sodium:

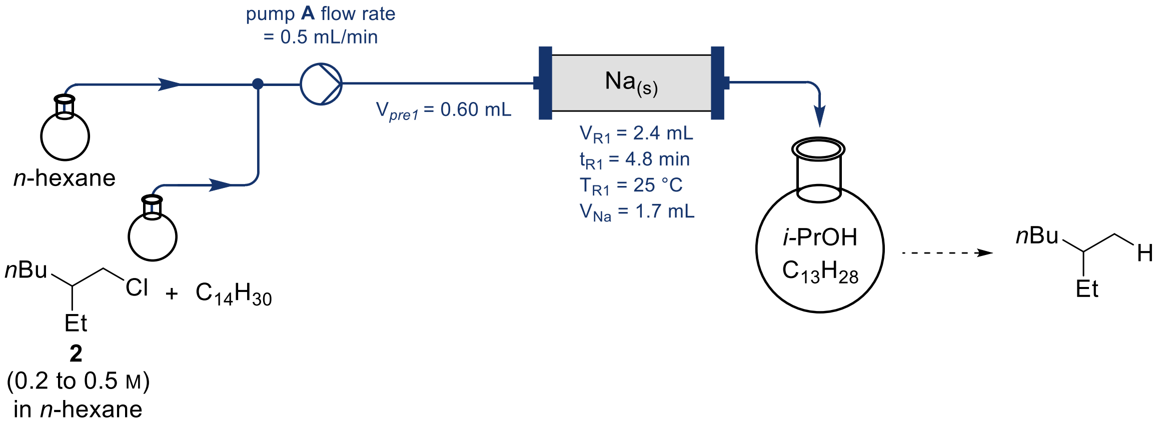

Scheme SI 2: Set-up for the solubility studies of (2-ethylhexyl)sodium (**1**).

A solution of 3-(chloromethyl)heptane (**2**, 0.2 M or 0.5 M) and tetradecane in *n*-hexane was prepared. After pumping *n*-hexane for 1 min, the solution of **2** was pumped through an activated sodium packed-bed reactor by pump A (flow rate: 0.5 mL/min) and injected for 20 s into 0.5 mL of a stock solution of *i*-PrOH (1.1 M) and tridecane in *n*-hexane. After 16 min, pump A was switched back from pumping the solution of **2** to pumping *n*-hexane. For the first 10 min a sample was taken every 20 s starting at minute 3. Starting from minute 10, an aliquot of 20 s was taken each minute until minute 32.

For comprehensibility, the highest value of the data set of the ratio of tetra- to tridecane (grey) and the hydrolysis product (orange) in figure SI 5 and 6 is normalized deliberately to 100%. Figure SI 5 shows that steady state is reached 9.0 minutes after switching from pumping *n*-hexane to pumping the solution of **2** at minute 1.0, as can be seen by the constant maximum level of the grey and orange curves. After switching back to pumping *n*-hexane at minute 16 (red line), the steady state is maintained for additional 4 min until minute 20. Furthermore, the blue curve shows a conversion of just 83% during steady state, which indicates a lack of activation. The conversion is increased before and after steady state, which can be explained by the lower concentration of 3-(chloromethyl)heptane (**2**). The ratio of tetra- to tridecane and the hydrolysis product correlate with each other, indicating a high solubility of (2-ethylhexyl)sodium (**1**). If the sodium species would precipitate in the sodium packed-bed reactor tailing of the hydrolysis product would be expected upon pumping *n*-hexane since additional solvent should dissolve the precipitated alkyl sodium species over time. Therefore, figure SI 5 displays the solubility of (2-ethylhexyl)sodium (**1**) using a 0.2 M solution of **2.**

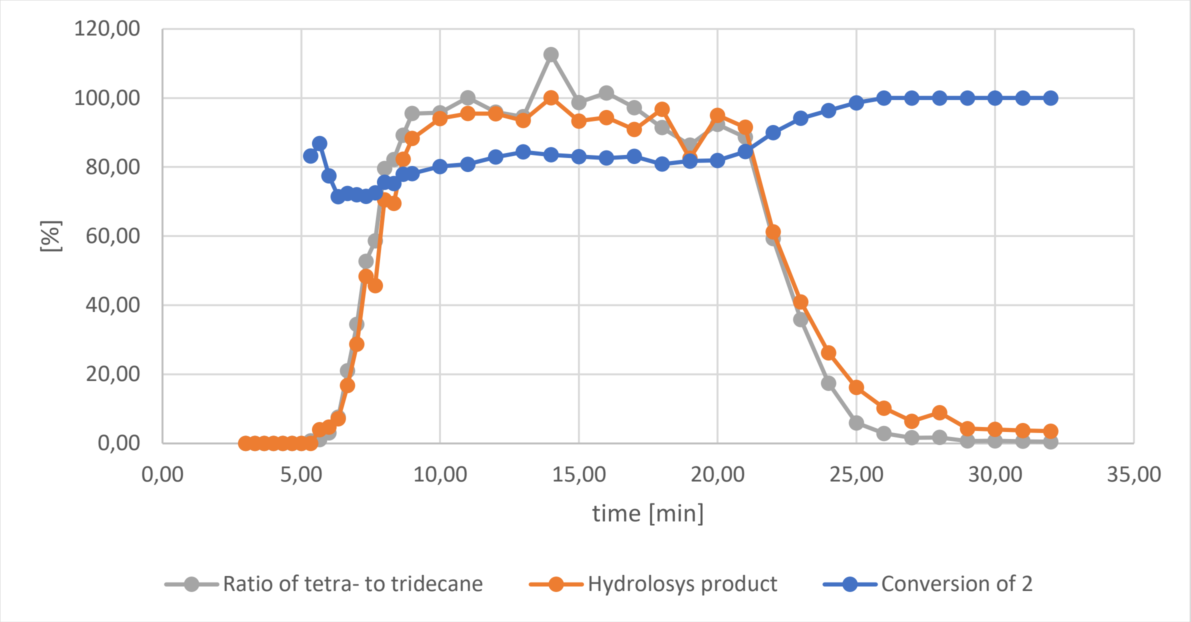

Figure SI 5: Solubility study of (2-ethylhexyl)sodium (**1**) using a 0.2 M solution of 3-(chloromethyl)heptane (**2**).

The same observation was made using a 0.5 M solution of **2**. The same correlation of the ratio of tetra- to tridecane (grey) with the hydrolysis product (orange) can be observed in figure SI 6 starting at minute 12. The lower values of the hydrolysis product compared to the ratio of tetra- to tridecane until minute 12 are in agreement with the lower values for the conversion of **2** (blue). At minute 12, the conversion reaches a peak level of about 96%.

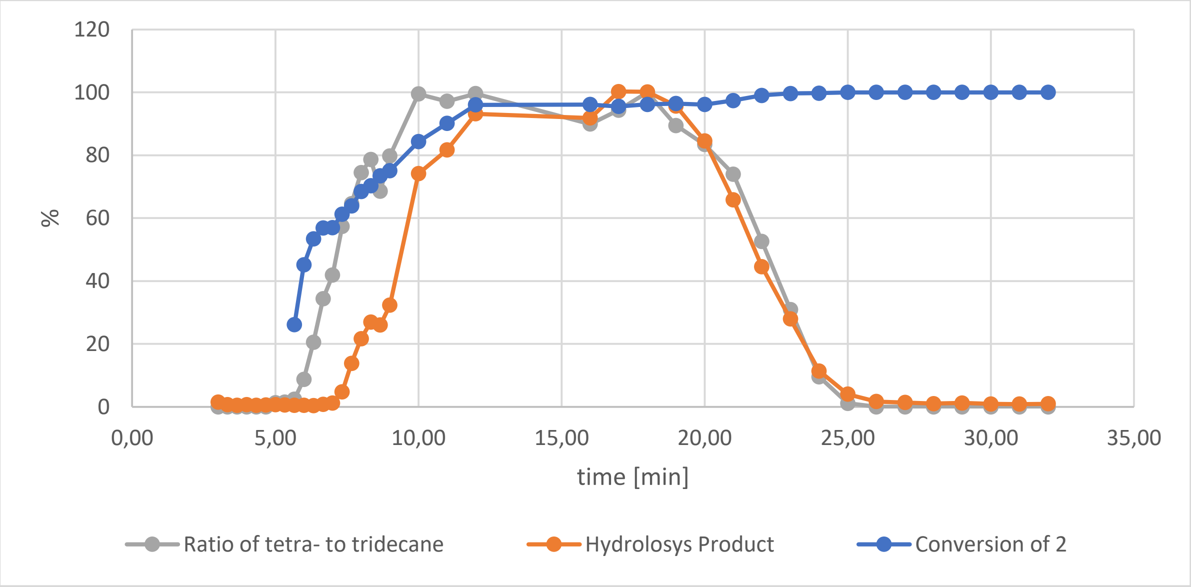

Figure SI 6: Solubility study of (2-ethylhexyl)sodium (**1**) using a 0.5 M solution of 3-(chloromethyl)heptane (**2**).

Optimization screening for the Br/Na-exchange

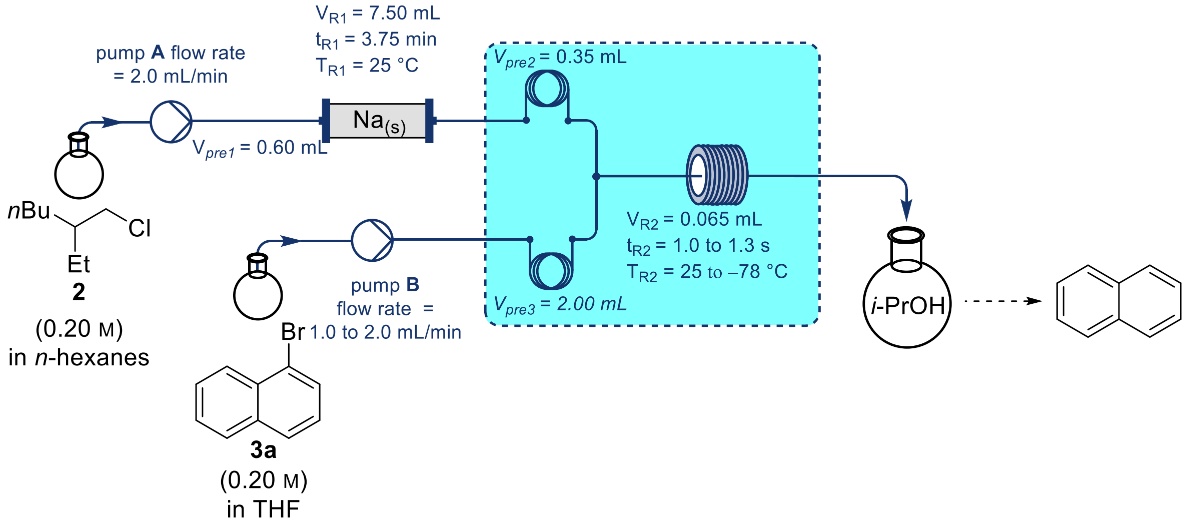

Scheme SI 3: Set-up for the screening of the on demand generation of (2-ethylhexyl)sodium (**1**) and subsequent in-line Br/Na-exchange.

Solutions of 1-bromonaphthalene (**3a**, 0.2 M) in THF, 3-(chloromethyl)heptane (**2**, 0.2 M) in *n*-hexane were prepared. The solution of **2** was pumped through the activated sodium packed-bed reactor (see **TP1**) by pump A (flow rate: 2.0 mL/min) into the precooling loop (V_{pre2} = 0.35 mL), which was cooled to the corresponding temperature (T_{R2} = 25 to –78 °C). The solution of **3a** was pumped by pump B (flow rate: 1.0 to 2.0 mL/min) through a precooling loop (V_{pre3} = 2.00 mL), which was cooled to the corresponding temperature (T_{R2} = 25 to –78 °C). The precooled solutions were mixed with an overall flow rate of 3.0 to 4.0 mL/min in a T-shaped mixer. The combined stream passed a tube reactor and a metal needle (V_{R2} = 0.065 mL, t_{R2} = 1.0 to 1.3 s), subsequently upon reaching the steady state, it was injected into a vial charged with a solution of *i*-PrOH (1.1 M) in *n*-hexane. Conversion of **3a** and formation of naphthalene was monitored by GC.

Table SI 2: Optimization screening for the Br/Na-exchange using (2-ethylhexyl)sodium (**1**) and 1-bromonaphthalene (**3a**).

Entry	Flow rate B [ml/min]	V_{R2} [ml]	t_{R2} [s]	T_{R2} [°C]	Conversion [%]	Normalized GC-Yield [a] [%]
1	2.0	0.065	1.0	25	56	53
2	1.8	0.065	1.0	25	67	59
3	1.5	0.065	1.1	25	64	71
4	1.2	0.065	1.2	25	82	82
5	1.0	0.065	1.3	25	93	93
6	2.0	0.065	1.0	0	79	62
7	1.8	0.065	1.0	0	69	68

8	1.5	0.065	1.1	0	83	73
9	1.2	0.065	1.2	0	78	66
10	1.0	0.065	1.3	0	84	81
11	2.0	0.065	1.0	−40	65	64
12	1.8	0.065	1.0	−40	70	70
13	1.5	0.065	1.1	−40	76	78
14	1.2	0.065	1.2	−40	90	95
15	1.0	0.065	1.3	−40	92	100
16	2.0	0.065	1.0	−78	72	49
17	1.8	0.065	1.0	−78	66	80
18	1.5	0.065	1.1	−78	74	70
19	1.2	0.065	1.2	−78	81	77

[a] The largest integrated area under the curve corresponding to naphthalene was normalized to 100% GC-yield the other integrals are adjusted accordingly.

Typical procedure 2 (TP2): On-demand synthesis of (2-ethylhexyl)sodium and its use in Br/Na exchange reactions.

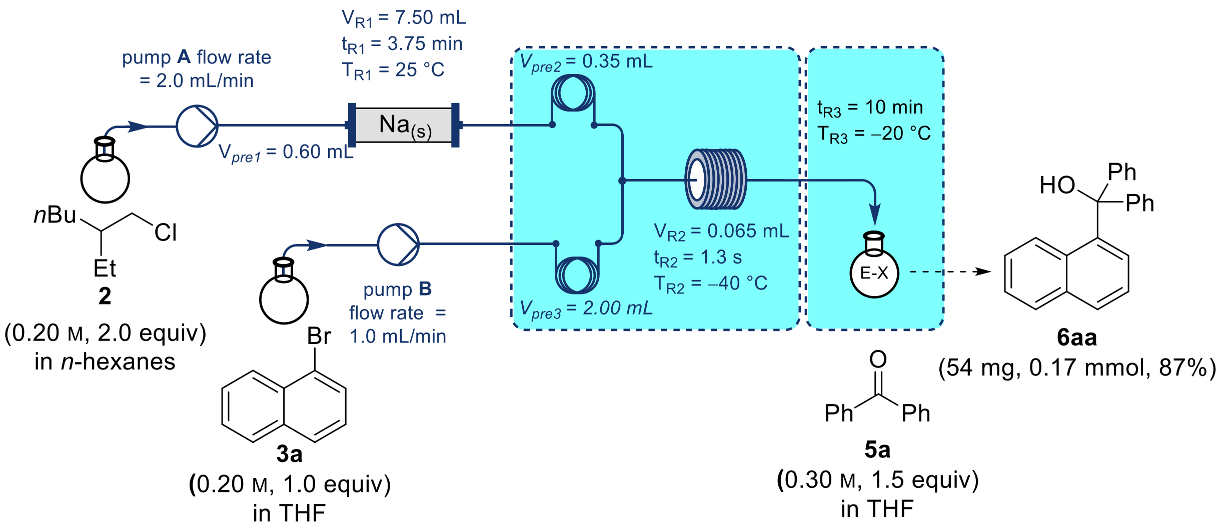

Scheme SI 4: Set-up for the on demand generation of (2-ethylhexyl)sodium (**1**), in-line Br/Na-exchange and subsequent batch quench with electrophiles.

Solutions of 1-bromonaphthalene (**3a**, 0.2 M, 1.0 equiv) in THF, 3-(chloromethyl)heptane (**2**, 0.2 M, 2.0 equiv) in *n*-hexane were prepared. The solution of **2** was pumped through the activated sodium packed-bed reactor (see **TP1**) by pump A (flow rate: 2.0 mL/min) into the precooling loop (V_{pre2} = 0.35 mL) cooled to –40 °C. The solution of **3a** was pumped by pump B (flow rate: 1.0 mL/min) through a precooling loop (V_{pre3} = 2.00 mL), which was cooled to –40 °C. The precooled solutions were mixed with an overall flow rate of 3.0 mL/min in a T-shaped mixer. The combined stream passed through a tube reactor and a metal needle (V_{R2} = 0.065 mL, t_{R2} = 1.3 s). Subsequently upon reaching the steady state, it was injected into a flask charged with benzophenone (**5a**, 55 mg, 0.3 mmol, 1.5 equiv) in THF (1.0 mL) at –20 °C for 60 s. The reaction mixture was stirred at –20 °C for 10 min and allowed to warm to 25 °C before sat. *aq.* NH_4Cl solution was added for quenching the reaction mixture. The aqueous layer was extracted three times with EtOAc (3×30 mL) and the combined organic layers were dried over anhydrous $MgSO_4$ and filtrated. After removal of the solvent, flash column chromatographical purification (silica gel, isohexane:EtOAc = 99:1 → 98:2) afforded the title compound **6aa** as a white solid (54 mg, 0.17 mmol, 87% yield).

Optimization screening for the metalation of benzothiophene

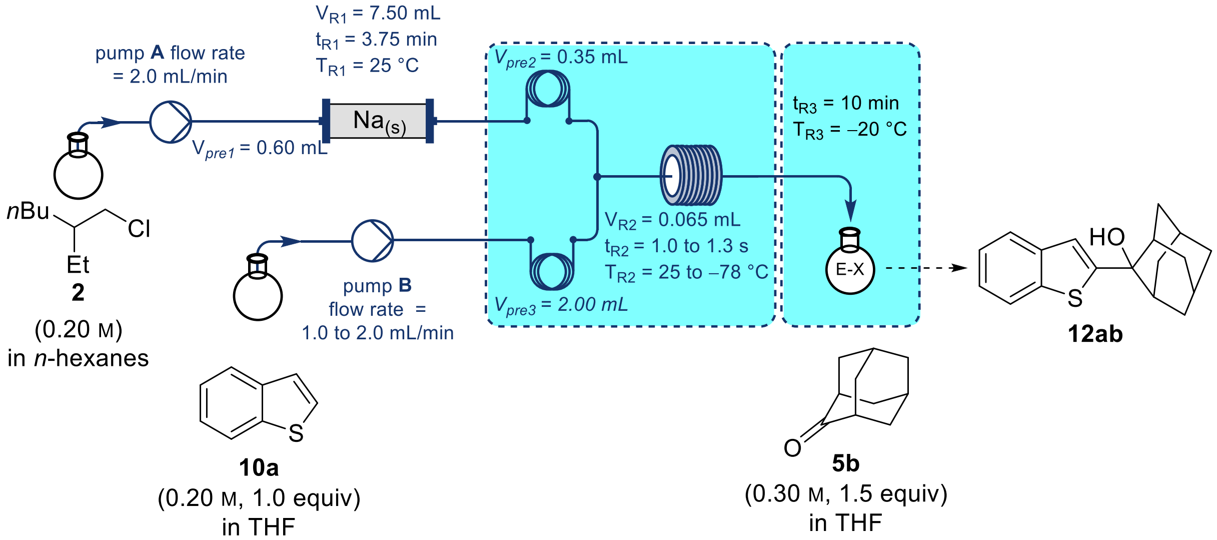

Scheme SI 5: Set-up for the optimization of the on demand generation of (2-ethylhexyl)sodium (**1**), in-line sodiation and subsequent batch quench with adamantanone.

Solutions of benzothiophene (**10a**, 0.2 M) in THF, 3-(chloromethyl)heptane (**2**, 0.2 M) in *n*-hexane were prepared. The solution of **2** was pumped through the activated sodium packed-bed reactor (see **TP1**) by pump A (flow rate: 2.0 mL/min) into the precooling loop (V_{pre2} = 0.35 mL), which was cooled to the corresponding temperature (T_{R2} = –40 to –78 °C). The solution of **10a** was pumped by pump B (flow rate: 1.0 to 2.0 mL/min) through a precooling loop (V_{pre3} = 2.00 mL) cooled to the corresponding temperature (T_{R2} = –40 to –78 °C). The precooled solutions were mixed with an overall flow rate of 3.0 to 4.0 mL/min in a T-shaped mixer. The combined stream passed through a tube reactor and a metal needle (V_{R2} = 0.065 to 2.0 mL, t_{R2} = 1.0 to 40 s). Subsequently upon reaching the steady state it was injected into a flask charged with adamantanone (**5b**, 45 mg, 0.3 mmol, 1.5 equiv) in THF (1.0 mL) cooled to –20 °C. Conversion of **10a** and formation of **12ab** was monitored by GC-analysis.

Table SI 3: Optimization screening for the directed sodiation of benzothiophene (**10a**) using (2-ethylhexyl)sodium (**1**).

Entry	Flowrate B [ml/min]	V_{R2} [ml]	t_{R2} [s]	T_{R2} [°C]	Conversion [%]	Normalized GC-Yield (**12ab**) [a] [%]
1	2.0	0.065	1.0	−40	69	64
2	1.8	0.065	1.0	−40	76	76
3	1.5	0.065	1.1	−40	80	75
4	1.2	0.065	1.2	−40	100	100
5	1.0	0.065	1.3	−40	100	94
6	2.0	2.0	30.0	−40	67	69
7	1.8	2.0	31.6	−40	73	74

8	1.5	2.0	34.3	−40	92	94
9	1.2	2.0	37.5	−40	96	96
10	1.0	2.0	40.0	−40	100	88

[[a] The largest integrated area under the curve corresponding to **12ab** was normalized to 100% GC-yield the other integrals are adjusted accordingly.

Typical procedure 3 (TP3): On-demand synthesis of (2-ethylhexyl)sodium and its use in metalation reactions.

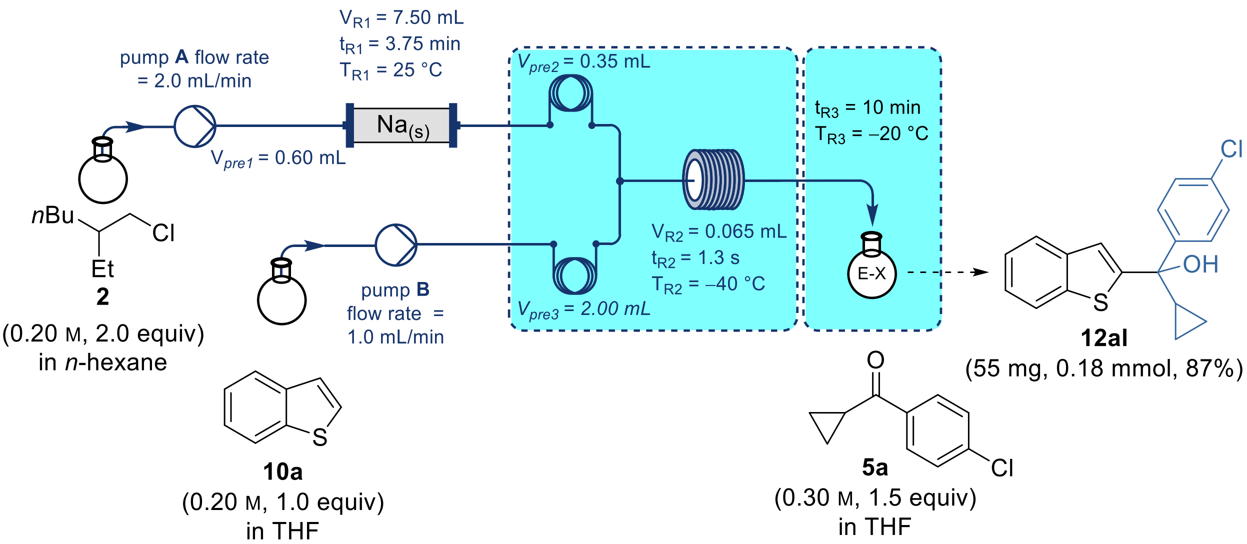

Scheme SI 6: Set-up for the on demand generation of (2-ethylhexyl)sodium (**1**), in-line sodiations and subsequent batch quench with electrophiles.

Solutions of benzothiophene (**10a**, 0.2 M, 1.0 equiv) in THF and 3-(chloromethyl)heptane (**2**, 0.2 M, 2.0 equiv) in *n*-hexane were prepared. The solution of **2** was pumped through the activated sodium packed-bed reactor (see **TP1**) by pump A (flow rate: 2.0 mL/min) into the precooling loop (V_{pre2} = 0.35 mL), which was cooled to –40 °C. The solution of **10a** was pumped by pump B (flow rate: 1.0 mL/min) through a precooling loop (V_{pre3} = 2.00 mL), which was cooled to –40 °C. The precooled solutions were mixed with an overall flow rate of 3.0 mL/min in a T-shaped mixer. The combined stream passed through a tube reactor and a metal needle (V_{R2} = 0.065 mL, t_{R2} = 1.3 s). Subsequently upon reaching the steady state, it was injected into a flask charged with (4-chlorophenyl)(cyclopropyl)methanone (**5l**, 54 mg, 0.3 mmol, 1.5 equiv) in THF (1.0 mL) cooled to –20 °C for 60 s. The reaction mixture was stirred at –20 °C for 10 min and allowed to warm to 25 °C before sat. *aq.* NH_4Cl solution was added for quenching the reaction mixture. The aqueous layer was extracted three times with EtOAc (3×30 mL) and the combined organic layers were dried over anhydrous $MgSO_4$ and filtrated. After removal of the solvent, flash column chromatographical purification (silica gel, isohexane:EtOAc = 99:1 → 9:1) afforded the title compound **12al** as a slightly brownish oil (55 mg, 0.18 mmol, 87% yield).

On-demand synthesis of (2-ethylhexyl)sodium and its use in a Br/Na-exchange reactions followed by an in-line quench.

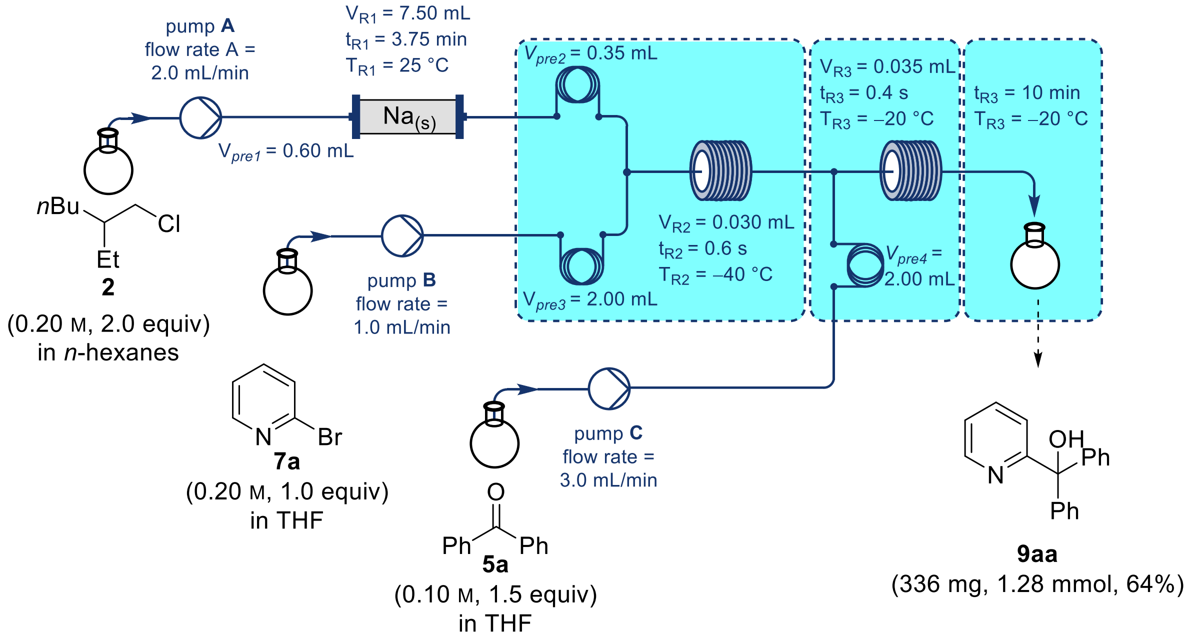

Scheme SI 7: Set-up for the on demand generation of (2-ethylhexyl)sodium (**1**), in-line Br/Na-exchange using 2-bromopyridine (**7a**) and subsequent in-line quench with benzophenone (**5a**) electrophiles.

Solutions of 2-bromopyridine (**7a**, 0.2 M, 1.0 equiv) in THF, 3-(chloromethyl)heptane (**2**, 0.2 M, 2.0 equiv) in *n*-hexane and benzophenone (**5a**, 0.1 M, 1.5 equiv) in THF were prepared. The solution of ar**2** was pumped through the activated sodium packed-bed reactor (see **TP1**) by pump A (flow rate: 2.0 mL/min) into the precooling loop (V_{pre2} = 0.35 mL) cooled to –40 °C. The solution of **7a** was pumped by pump B (flow rate: 1.0 mL/min) through a precooling loop (V_{pre3} = 2.00 mL), which was cooled to –40 °C. The precooled solutions were mixed with an overall flow rate of 3.0 mL/min in a T-shaped mixer. The combined stream passed through a tube reactor (V_{R2} = 0.030 mL, t_{R2} = 0.6 s). Pump C (flow rate: 3.0 mL/min) pumped the solution of benzophenone **5a**, through a precooling loop (V_{pre4} = 2.00 mL), which was cooled to –20 °C. The two streams were mixed in another T-shaped mixer, the combined reaction mixture passed a metal needle (V_{R3} = 0.035 mL) and was, subsequently upon reaching the steady state, injected into an argon filled flask cooled to –20 °C for 10 or 17.5 min. The reaction mixture was stirred at –20 °C for 10 min and allowed to warm to 25 °C before sat. *aq.* NH_4Cl solution was added for quenching the reaction mixture. The aqueous layer was extracted three times with EtOAc (3×70 mL) and the combined organic layers were dried over anhydrous $MgSO_4$ and filtrated. After removal of the solvent, flash column chromatographical purification (silica gel, isohexane:EtOAc

= 95:5 → 90:10) afforded the title compound **9aa** as colorless crystals (10 min runtime: 336 mg, 1.28 mmol, 64% yield; 17.5 min runtime: 592 mg, 2.27 mmol, 65% yield).

Typical procedure 4 (TP4): Batch preparation of neopentyl chloride followed by Br/Na-exchange and aldehyde quench.

According to the procedure of Asako, Takai and co-workers[2] sodium dispersion (30 wt% in toluene, particle size <0.1 mm, 1.05 mmol, 4.2 equiv) was added into a flame dried round bottom flask charged with a stirring bar, the toluene was removed *in vacuo* n-hexane (2.0 mL) was added. Neopentyl chloride was added (53 mg, 0.50 mmol, 2.0 equiv) at 0 °C. The mixture was stirred at 0 °C for 20 min before **3a** (52 mg, 0.25 mmol, 1.0 equiv) was added at 0 °C and the mixture was again stirred at this temperature for 30 min. **5b** (30 mg, 0.30 mmol, 1.2 equiv) was added at 0 °C the mixture was allowed to warm to 25 °C and stirred for 30 min before it was quenched with NH_4Cl. The aqueous layer was extracted three times with EtOAc (3×70 mL) and the combined organic layers were dried over anhydrous $MgSO_4$ and filtrated. After removal of the solvent, flash column chromatographical purification (silica gel, pentane:EtOAc = 95:5) afforded the title compound **6ab** as colorless crystals (36 mg, 0.16 mmol, 63% yield).

[2] S. Asako, I. Takahashi, H. Nakajama, L. Ilies, K. Takai, **2020**, ChemRxiv preprint DOI 10.26434/chemrxiv.12378104.v1

Preparation of the products

3-(chloromethyl)heptane (2)

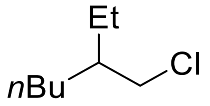

Thionyl chloride (61.7 mL, 0.85 mol, 1.7 equiv) was added over 1 h to a solution of 2-ethylhexan-1-ol (78.5 mL, 0.50 mmol, 1.0 equiv) and pyridine (23 mL) at 0 °C. The reaction mixture was stirred over night at 55 °C. Afterwards the mixture was quenched with H_2O (100 mL) at 0 °C. The aqueous layer was extracted three times with EtOAc (3×100 mL) and the combined organic layers were dried over anhydrous $MgSO_4$ and filtrated. After removal of the solvent, flash column chromatographical purification (silica gel, isohexane:EtOAc = 99.5:0.5) afforded the title compound **2** as colorless oil (66 g, 0.45 mmol, 89% yield).

^{1}H-NMR (400 MHz, $CDCl_3$): δ / ppm = 3.58 – 3.48 (m, 2H), 1.59 (m, 1H), 1.51 – 1.12 (m, 8H), 0.90 (m, 6H)

^{13}C-NMR (100 MHz, $CDCl_3$): δ / ppm = 48.4, 41.6, 31.0, 29.0, 24.3, 23.0, 14.1, 10.9.

683.

MS (EI, 70 eV): *m/z* (%) = 83 (41), 70 (15), 57 (100), 55 (29), 41 (71).

HRMS (EI-orbitrap): *m/z:* [M – C_2H_5] calc. for [$C_6H_{12}Cl$]: 119.0628; found 119.0622.

Naphthalen-1-yldiphenylmethanol (6aa)

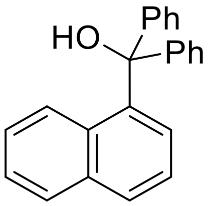

According to **TP2**, solutions of 1-bromonaphthalene (**3a**, 0.2 M, 1.0 equiv) in THF, 3-(chloromethyl)heptane (**2**, 0.2 M, 2.0 equiv) in *n*-hexane were prepared. The solution of **2** was pumped through the activated sodium packed-bed reactor (see **TP1**) by pump A (flow rate: 2.0 mL/min) into the precooling loop (V_{pre2} = 0.35 mL) cooled to –40 °C. The solution of **3a** was pumped by pump B (flow rate: 1.0 mL/min) through a precooling loop (V_{pre3} = 2.00 mL) cooled to –40 °C. The precooled solutions were mixed with an overall flow rate of 3.0 mL/min in a T-shaped mixer. The combined stream passed through a tube reactor and a metal needle (V_{R2} = 0.065 mL, t_{R2} = 1.3 s), subsequently upon reaching the steady state, it was injected into a flask charged with benzophenone (**5a**, 55 mg, 0.3 mmol, 1.5 equiv) in THF (1.0 mL) at –20 °C for 60 s. The reaction mixture was stirred at –20 °C for 10 min and allowed to warm to 25 °C before sat. *aq.* NH_4Cl solution was added for quenching the reaction mixture. The aqueous layer was extracted three times with EtOAc (3×30 mL) and the combined organic layers were dried over anhydrous $MgSO_4$ and filtrated. After removal of the solvent, flash column chromatographical purification (silica gel, isohexane:EtOAc = 99:1 → 98:2) afforded the title compound **6aa** as colorless crystals (54 mg, 0.17 mmol, 87% yield).

^{1}H-NMR (400 MHz, $CDCl_3$): δ / ppm = 8.12 (d, *J* = 8.7 Hz, 1H), 7.86 (d, *J* = 8.0 Hz, 1H), 7.82 (d, *J* = 8.2 Hz, 1H), 7.41 (t, *J* = 7.5 Hz, 1H), 7.36 – 7.26 (m, 12H), 6.88 (dd, J = 7.3, 0.9 Hz, 1H), 3.35 (s, 1H).

^{13}C-NMR (100 MHz, $CDCl_3$): δ / ppm = 147.1 (2C), 142.2, 135.1, 131.4, 129.5, 129.0, 128.3, 128.2 (5C), 127.9 (4C), 127.3 (2C), 125.7, 125.5, 124.4, 83.4.

IR (Diamond-ATR, neat): $\tilde{\nu}$ / cm^{-1} = 3560, 1490, 1446, 1396, 1329, 1174, 1165, 1157, 1151, 1143, 1032,

155 (15), 155 (23), 128 (20, 127 (16), 105 (100), 77 (54).

HRMS (EI-orbitrap): *m/z:* [M] calc. for [$C_{23}H_{18}O$]: 310.1358; found 310.1352.

m.p. (°C): 129.8 – 135.5.

2-Ethyl-1-(naphthalen-1-yl)butan-1-ol (6ab)

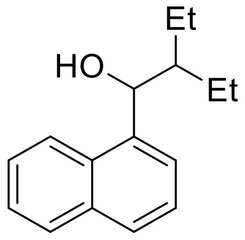

According to **TP2**, solutions of 1-bromonaphthalene (**3a**, 0.2 M, 1.0 equiv) in THF, 3-(chloromethyl)heptane (**2**, 0.2 M, 2.0 equiv) in *n*-hexane were prepared. The solution of **2** was pumped through the activated sodium packed-bed reactor (see **TP1**) by pump A (flow rate: 2.0 mL/min) into the precooling loop (V_{pre2} = 0.35 mL) cooled to –40 °C. The solution of **3a** was pumped by pump B (flow rate: 1.0 mL/min) through a precooling loop (V_{pre3} = 2.00 mL) cooled to –40 °C. The precooled solutions were mixed with an overall flow rate of 3.0 mL/min in a T-shaped mixer. The combined stream passed through a tube reactor and a metal needle (V_{R2} = 0.065 mL, t_{R2} = 1.3 s), subsequently upon reaching the steady state, it was injected into a flask charged with 2-ethylbutanal (**5b**, 30 mg, 0.3 mmol, 1.5 equiv) in THF (1.0 mL) at –20 °C for 60 s. The reaction mixture was stirred at –20 °C for 10 min and allowed to warm to 25 °C before sat. *aq.* NH_4Cl solution was added for quenching the reaction mixture. The aqueous layer was extracted three times with EtOAc (3×30 mL) and the combined organic layers were dried over anhydrous $MgSO_4$ and filtrated. After removal of the solvent, flash column chromatographical purification (silica gel, isohexane:EtOAc = 99:1 → 9:1) afforded the title compound **6ab** as a colorless oil (31 mg, 0.14 mmol, 70% yield).

According to **TP4**, sodium dispersion (30 wt% in toluene, particle size <0.1 mm, 1.05 mmol, 4.2 equiv) was added into a flame dried round bottom flask charged with a stirring bar, the toluene was removed *in vacuo* n-hexane (2.0 mL) was added. Neopentyl chloride was added (53 mg, 0.50 mmol, 2.0 equiv) at 0 °C. The mixture was stirred at 0 °C for 20 min before **3a** (52 mg, 0.25 mmol, 1.0 equiv) was added at 0 °C and the mixture was again stirred at this temperature for 30 min. **5b** (30 mg, 0.30 mmol, 1.2 equiv) was added at 0 °C the mixture was allowed to warm to 25 °C and stirred for 30 min before it was quenched with NH_4Cl. The aqueous layer was extracted three times with EtOAc (3×70 mL) and the combined organic layers were dried over anhydrous $MgSO_4$ and filtrated. After removal of the solvent, flash column chromatographical purification (silica gel, pentane:EtOAc = 95:5) afforded in our hands the title compound **6ab** as colorless oil (36 mg, 0.16 mmol, 63% yield).

^{1}H-NMR (400 MHz, $CDCl_3$): δ / ppm = 8.16 – 8.05 (m, 1H), 7.92 – 7.83 (m, 1H), 7.83 – 7.74 (m, 1H), 7.65 (m, 1H), 7.55 – 7.42 (m, 3H), 5.50 (dd, *J* = 5.6, 3.1 Hz, 1H), 1.82 (dd, *J* = 7.8, 3.7 Hz, 2H), 1.63 – 1.29 (m, 4H), 0.99 (t, *J* = 7.4 Hz, 3H), 0.82 (t, *J* = 7.5 Hz, 3H).

^{13}C-NMR (100 MHz, $CDCl_3$): δ / ppm = 139.9, 133.9, 130.7, 129.1, 127.9, 125.9, 125.5, 125.4, 124.2, 123.4, 72.5, 46.7, 22.9, 20.4, 11.8, 11.2.

IR (Diamond-ATR, neat): $\tilde{\nu}$ / cm^{-1} = 3414, 3335, 3048, 2958, 2931, 2872, 1596, 1510, 1458, 1394, 1378, 1344, 1308, 1305, 1260, 1227, 1166, 1123, 1107, 1079, 1036, 1021, 999, 954, 944, 912, 863, 858, 799, 776, 747, 731.

MS (EI, 70 eV): *m/z* (%) = 158 (12), 157 (100), 129 (79), 128 (35).

HRMS (EI-orbitrap): *m/z:* [M] calc. for [$C_{16}H_{20}O$]: 228.1514; found 228.1508.

(3,5-Di-tert-butylphenyl)(4-(trifluoromethyl)phenyl)methanone (6bc)

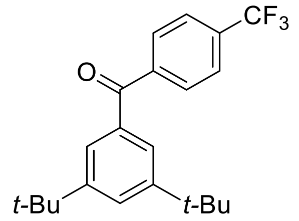

According to **TP2**, solutions of 1-bromo-3,5-di-*tert*-butylbenzene (**3b**, 0.2 M, 1.0 equiv) in THF, 3-(chloromethyl)heptane (**2**, 0.2 M, 2.0 equiv) in *n*-hexane were prepared. The solution of **2** was pumped through the activated sodium packed-bed reactor (see **TP1**) by pump A (flow rate: 2.0 mL/min) into the precooling loop (V_{pre2} = 0.35 mL) cooled to –40 °C. The solution of **3b** was pumped by pump B (flow rate: 1.0 mL/min) through a precooling loop (V_{pre3} = 2.00 mL) cooled to –40 °C. The precooled solutions were mixed with an overall flow rate of 3.0 mL/min in a T-shaped mixer. The combined stream passed through a tube reactor and a metal needle (V_{R2} = 0.065 mL, t_{R2} = 1.3 s), subsequently upon reaching the steady state, it was injected into a flask charged with *N*-methoxy-*N*-methyl-4-(trifluoromethyl)benzamide (**5c**, 70 mg, 0.3 mmol, 1.5 equiv) in THF (1.0 mL) at –20 °C for 60 s. The reaction mixture was stirred at –20 °C for 10 min and allowed to warm to 25 °C before sat. *aq.* NH_4Cl solution was added for quenching the reaction mixture. The aqueous layer was extracted three times with EtOAc (3×30 mL) and the combined organic layers were dried over anhydrous $MgSO_4$ and filtrated. After removal of the solvent, flash column chromatographical purification (silica gel, isohexane:EtOAc = 99.5:0.5 → 99:1) afforded the title compound **6bc** as colorless crystals (53 mg, 0.15 mmol, 73% yield).

^{1}H-NMR (400 MHz, $CDCl_3$): δ / ppm = 7.91 (d, *J* = 8.1 Hz, 2H), 7.76 (d, *J* = 8.2 Hz, 2H), 7.70 (t, *J* = 1.8 Hz, 1H), 7.63 (d, *J* = 1.8 Hz, 2H), 1.35 (s, 18H).

^{13}C-NMR (100 MHz, $CDCl_3$): δ / ppm = 196.4, 151.4 (2C), 141.3, 136.4, 133.7 (q, *J* = 32.8 Hz), 130.4 (2C), 127.4, 125.4 (q, *J* = 3.8 Hz, 2C), 124.6 (2C), 123.9 (q, *J* = 271.8 Hz), 35.2 (2C), 31.5 (6C).

IR (Diamond-ATR, neat): $\tilde{\nu}$ / cm^{-1} = 2964, 2907, 2870, 1653, 1595, 1476, 1466, 1450, 1404, 1397, 1364, 1322, 1314, 1281, 1246, 1161, 1139, 1118, 1107, 1065, 1015, 988, 963, 899, 893, 857, 839, 776, 764, 753, 726, 705, 679.

MS (EI, 70 eV): *m/z* (%) = 348 (23), 347 (100), 173 (47), 145 (16).

HRMS (EI-orbitrap): *m/z:* [M] calc. for [$C_{22}H_{25}F_3O$]: 362.1858; found 362.1853.

m.p. (°C): 86.3 – 89.9.

N-((3,5-Di-tert-butylphenyl)(phenyl)methyl)aniline (6bd)

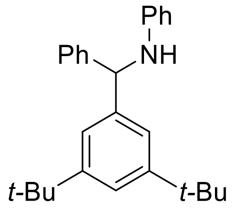

According to **TP2**, solutions of 1-bromo-3,5-di-*tert*-butylbenzene (**3b**, 0.2 M, 1.0 equiv) in THF, 3-(chloromethyl)heptane (**2**, 0.2 M, 2.0 equiv) in *n*-hexane were prepared. The solution of **2** was pumped through the activated sodium packed-bed reactor (see **TP1**) by pump A (flow rate: 2.0 mL/min) into the precooling loop (V_{pre2} = 0.35 mL) cooled to –40 °C. The solution of **3b** was pumped by pump B (flow rate: 1.0 mL/min) through a precooling loop (V_{pre3} = 2.00 mL) cooled to –40 °C. The precooled solutions were mixed with an overall flow rate of 3.0 mL/min in a T-shaped mixer. The combined stream passed through a tube reactor and a metal needle (V_{R2} = 0.065 mL, t_{R2} = 1.3 s), subsequently upon reaching the steady state, it was injected into a flask charged with *N*,1-diphenylmethanimine (**5d**, 54 mg, 0.3 mmol, 1.5 equiv) in THF (1.0 mL) at –20 °C for 60 s. The reaction mixture was stirred at –20 °C for 10 min and allowed to warm to 25 °C before sat. *aq.* NH_4Cl solution was added for quenching the reaction mixture. The aqueous layer was extracted three times with EtOAc (3×30 mL) and the combined organic layers were dried over anhydrous $MgSO_4$ and filtrated. After removal of the solvent, flash column chromatographical purification (silica gel, isohexane:EtOAc = 99.5:0.5 → 99:1) afforded the title compound **6bd** as a colorless solid (48 mg, 0.13 mmol, 65% yield).

^{1}H-NMR (400 MHz, $CDCl_3$): δ / ppm = 7.43 (d, *J* = 7.3 Hz, 2H), 7.38 – 7.31 (m, 3H), 7.29 – 7.23 (m, 1H), 7.20 – 7.09 (m, 4H), 6.70 (t, *J* = 7.3 Hz, 1H), 6.58 (d, *J* = 7.8 Hz, 2H), 5.50 (s, 1H), 4.29 (s, 1H), 1.30 (s, 18H).

^{13}C-NMR (100 MHz, $CDCl_3$): δ / ppm = 151.2 (2C), 147.7, 143.1, 142.5, 129.2 (2C), 128.7 (2C), 127.3 (2C), 127.1, 122.1 (2C), 121.5, 117.5, 113.6 (2C), 63.8, 35.0 (2C), 31.6 (6C).

IR (Diamond-ATR, neat): $\tilde{\nu}$ / cm^{-1} = 3411, 2960, 2951, 2928, 2923, 2902, 2863, 1599, 1501, 1491, 1476, 1464, 1454, 1424, 1390, 1360, 1327, 1316, 1269, 1262, 1244, 1202, 1181, 1159, 1154, 1130, 1105, 1073, 1034, 1026, 990, 923, 896, 871, 863, 759, 748, 718, 700, 691.

MS (EI, 70 eV): *m/z* (%) = 280 (13), 279 (58), 263 (36), 191 (13), 182 (42), 180 (18), 179 (27), 178 (29), 174 (10), 167 (10), 165 (35), 152 (11), 129 (12), 128 (14), 115 (15), 104 (13), 93 (100), 92 (71), 91 (18), 77 (25), 65 (32), 57 (16), 41 (12).

HRMS (EI-orbitrap): *m/z:* [M] calc. for [$C_{27}H_{33}N$]: 371.2613; found 371.2611.

m.p. (°C): 115.1 – 122.7.

***N*-((4-Chlorophenyl)(phenyl)methyl)aniline (6cd)**

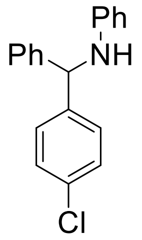

According to **TP2**, solutions of 1-bromo-4-chlorobenzene (**3c**, 0.2 M, 1.0 equiv) in THF, 3-(chloromethyl)heptane (**2**, 0.2 M, 2.0 equiv) in *n*-hexane were prepared. The solution of **2** was pumped through the activated sodium packed-bed reactor (see **TP1**) by pump A (flow rate: 2.0 mL/min) into the precooling loop (V_{pre2} = 0.35 mL) cooled to –40 °C. The solution of **3c** was pumped by pump B (flow rate: 1.0 mL/min) through a precooling loop (V_{pre3} = 2.00 mL) cooled to –40 °C. The precooled solutions were mixed with an overall flow rate of 3.0 mL/min in a T-shaped mixer. The combined stream passed through a tube reactor and a metal needle (V_{R2} = 0.065 mL, t_{R2} = 1.3 s), subsequently upon reaching the steady state, it was injected into a flask charged with *N*,1-diphenylmethanimine (**5d**, 54 mg, 0.3 mmol, 1.5 equiv) in THF (1.0 mL) at –20 °C for 60 s. The reaction mixture was stirred at –20 °C for 10 min and allowed to warm to 25 °C before sat. *aq.* NH_4Cl solution was added for quenching the reaction mixture. The aqueous layer was extracted three times with EtOAc (3×30 mL) and the combined organic layers were dried over anhydrous $MgSO_4$ and filtrated. After removal of the solvent, flash column chromatographical purification (silica gel, isohexane:EtOAc = 100:0 → 99:1) afforded the title compound **6cd** as a colorless oil (36 mg, 0.12 mmol, 62% yield).

^{1}H-NMR (400 MHz, $CDCl_3$): δ / ppm = 7.41 – 7.23 (m, 9H), 7.17 – 7.11 (m, 2H), 6.73 (tt, *J* = 7.3, 1.1 Hz, 1H), 6.59 – 6.49 (m, 2H), 5.48 (d, *J* = 2.3 Hz, 1H), 4.20 (s, 1H).

^{13}C-NMR (100 MHz, $CDCl_3$): δ / ppm = 147.2, 142.7, 141.4, 133.2, 129.3 (2C), 129.0 (4C), 128.8 (2C), 127.8, 127.6 (2C), 118.0, 113.6 (2C), 62.6.

IR (Diamond-ATR, neat): $\tilde{\nu}$ / cm^{-1} =3412, 3050, 3026, 1600, 1577, 1500, 1487, 1451, 1426, 1405, 1338, 1312, 1265, 1240, 1180, 1154, 1116, 1089, 1077, 1065, 1029, 1014, 1005, 992, 871, 845, 835, 819, 797, 749, 719, 698, 692, 673, 668.

MS (EI, 70 eV): *m/z* (%) = 203 (10), 201 (29), 166 (31), 166 (13), 165 (100), 164 (15), 163 (13), 92 (11), 77 (25), 65 (16).

HRMS (EI-orbitrap): *m/z:* [M] calc. for [$C_{19}H_{16}NCl$]: 293.0971; found 293.0966.

(4-Chlorophenyl)dicyclopropylmethanol (6ce)

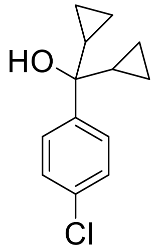

According to **TP2**, solutions of 1-bromo-4-chlorobenzene (**3c**, 0.2 M, 1.0 equiv) in THF, 3-(chloromethyl)heptane (**2**, 0.2 M, 2.0 equiv) in *n*-hexane were prepared. The solution of **2** was pumped through the activated sodium packed-bed reactor (see **TP1**) by pump A (flow rate: 2.0 mL/min) into the precooling loop (V_{pre2} = 0.35 mL) cooled to –40 °C. The solution of **3c** was pumped by pump B (flow rate: 1.0 mL/min) through a precooling loop (V_{pre3} = 2.00 mL) cooled to –40 °C. The precooled solutions were mixed with an overall flow rate of 3.0 mL/min in a T-shaped mixer. The combined stream passed through a tube reactor and a metal needle (V_{R2} = 0.065 mL, t_{R2} = 1.3 s), subsequently upon reaching the steady state, it was injected into a flask charged with dicyclopropylmethanone (**5e**, 33 mg, 0.3 mmol, 1.5 equiv) in THF (1.0 mL) at –20 °C for 60 s. The reaction mixture was stirred at –20 °C for 10 min and allowed to warm to 25 °C before sat. *aq.* NH_4Cl solution was added for quenching the reaction mixture. The aqueous layer was extracted three times with EtOAc (3×30 mL) and the combined organic layers were dried over anhydrous $MgSO_4$ and filtrated. After removal of the solvent, flash column chromatographical purification (silica gel, isohexane:EtOAc = 98:2 → 9:1) afforded the title compound **6ce** as a colorless oil (37 mg, 0.17 mmol, 83% yield).

^{1}H-NMR (400 MHz, $CDCl_3$): δ / ppm = 7.55 – 7.47 (m, 2H), 7.32 – 7.27 (m, 2H), 1.46 (s, 1H), 1.21 – 1.10 (m, 2H), 0.61 – 0.49 (m, 4H), 0.43 – 0.30 (m, 4H).

^{13}C-NMR (100 MHz, $CDCl_3$): δ / ppm = 145.9, 132.6, 128.0 (2C), 127.4 (2C), 73.7, 20.7 (2C), 2.2 (2C), 0.3 (2C).

IR (Diamond-ATR, neat): $\tilde{\nu}$ / cm^{-1} = 3591, 3581, 3470, 3415, 3085, 3007, 2926, 1594, 1488, 1461, 1425, 1399, 1376, 1313, 1193, 1156, 1091, 1051, 1025, 1013, 994, 967, 943, 912, 871, 851, 816, 779, 722, 714.

MS (EI, 70 eV): *m/z* (%) = 196 (29), 195 (10), 194 (85), 183 (31), 182 (11), 181 (86), 179 (11), 159 (55), 152 (19), 144 (18), 141 (17), 141 (28), 139 (100), 128 (15), 125 (19), 115 (17), 111 (29), 91 (12), 77 (11), 75 (13), 69 (50), 44 (31), 43 (10), 41 (38).

HRMS (EI-orbitrap): *m/z:* [M] calc. for [$C_{13}H_{15}ClO$]: 222.0811; found 222.0799.

Diphenyl(2-(trifluoromethyl)phenyl)methanol (6da)

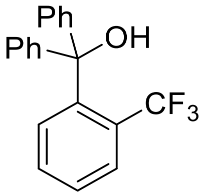

According to **TP2**, solutions of 1-bromo-2-(trifluoromethyl)benzene (**3d**, 0.2 M, 1.0 equiv) in THF, 3-(chloromethyl)heptane (**2**, 0.2 M, 2.0 equiv) in *n*-hexane were prepared. The solution of **2** was pumped through the activated sodium packed-bed reactor (see **TP1**) by pump A (flow rate: 2.0 mL/min) into the precooling loop (V_{pre2} = 0.35 mL) cooled to –40 °C. The solution of **3d** was pumped by pump B (flow rate: 1.0 mL/min) through a precooling loop (V_{pre3} = 2.00 mL) cooled to –40 °C. The precooled solutions were mixed with an overall flow rate of 3.0 mL/min in a T-shaped mixer. The combined stream passed through a tube reactor and a metal needle (V_{R2} = 0.065 mL, t_{R2} = 1.3 s), subsequently upon reaching the steady state, it was injected into a flask **charged** with benzophenone (**5a**, 55 mg, 0.3 mmol, 1.5 equiv) in THF (1.0 mL) at –20 °C for 60 s. The reaction mixture was stirred at –20 °C for 10 min and allowed to warm to 25 °C before sat. *aq.* NH_4Cl solution was added for quenching the reaction mixture. The aqueous layer was extracted three times with EtOAc (3×30 mL) and the combined organic layers were dried over anhydrous $MgSO_4$ and filtrated. After removal of the solvent, flash column chromatographical purification (silica gel, isohexane:EtOAc = 99:1 → 98:2) afforded the title compound **6da** as colorless crystals (59 mg, 0.18 mmol, 90% yield).

^{1}H-NMR (400 MHz, $CDCl_3$): δ / ppm = 7.83 (d, *J* = 7.9 Hz, 1H), 7.42 (t, *J* = 7.8 Hz, 1H), 7.37 – 7.27 (m, 7H), 7.18 – 7.10 (m, 4H), 6.86 (d, *J* = 7.8 Hz, 1H), 3.33 – 3.30 (m, 1H).
^{13}C-NMR (100 MHz, $CDCl_3$): δ / ppm = 147.5, 145.2 (q, *J* = 1.6 Hz), 132.5 (2C), 130.6, 129.4 (q, *J* =31.9 Hz), 128.6 (q, *J* = 6.7 Hz), 128.2 (4C), 128.1 (q, *J* = 22.3 Hz), 127.8 (4C), 127.8, 127.6 (2C), 124.8 (q, *J* = 274.5 Hz) 83.3.
IR (Diamond-ATR, neat): $\tilde{\nu}$ / cm^{-1} = 3638, 3055, 1657, 1598, 1584, 1577, 1490, 1446, 1440, 1317, 1303, 1274, 1213, 1187, 1173, 1156, 1142, 1124, 1102, 1088, 1081, 1061, 1035, 1020, 1001, 986, 976, 960, 941, 919, 907, 894, 876, 872, 853, 841, 833, 809, 782, 763, 751, 697, 659, 654.
MS (EI, 70 eV): *m/z* (%) = 328 (16), 231 (52), 212 (15), 211 (100), 184 (15), 183 (98), 183 (44), 173 (13), 165 (13), 155 (56), 154 (44), 145 (19), 105 (74), 77 (32).
HRMS (EI-orbitrap): *m/z:* [M] calc. for [$C_{20}H_{15}F_3O$]: 328.1075; found 328.1067.
m.p. (°C): 117.7 – 118. 8.

2-(2-(Trifluoromethyl)phenyl)adamantan-2-ol (6df)

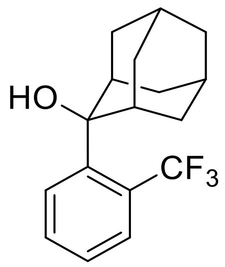

According to **TP2**, solutions of 1-bromo-2-(trifluoromethyl)benzene (**3d**, 0.2 M, 1.0 equiv) in THF, 3-(chloromethyl)heptane (**2**, 0.2 M, 2.0 equiv) in *n*-hexane were prepared. The solution of **2** was pumped through the activated sodium packed-bed reactor (see **TP1**) by pump A (flow rate: 2.0 mL/min) into the precooling loop (V_{pre2} = 0.35 mL) cooled to –40 °C. The solution of **3d** was pumped by pump B (flow rate: 1.0 mL/min) through a precooling loop (V_{pre3} = 2.00 mL) cooled to –40 °C. The precooled solutions were mixed with an overall flow rate of 3.0 mL/min in a T-shaped mixer. The combined stream passed through a tube reactor and a metal needle (V_{R2} = 0.065 mL, t_{R2} = 1.3 s), subsequently upon reaching the steady state, it was injected into a flask charged with adamantanone (**5f**, 45 mg, 0.3 mmol, 1.5 equiv) in THF (1.0 mL) at –20 °C for 60 s. The reaction mixture was stirred at –20 °C for 10 min and allowed to warm to 25 °C before sat. *aq.* NH_4Cl solution was added for quenching the reaction mixture. The aqueous layer was extracted three times with EtOAc (3×30 mL) and the combined organic layers were dried over anhydrous $MgSO_4$ and filtrated. After removal of the solvent, flash column chromatographical purification (silica gel, isohexane:EtOAc = 99:1 → 98:2) afforded the title compound **6df** as colorless crystals (45 mg, 0.15 mmol, 76% yield).

^{1}H-NMR (400 MHz, CDCl3): δ / ppm = 7.77 (dd, *J* = 8.0, 3.4 Hz, 2H), 7.54 (t, *J* = 7.7 Hz, 1H), 7.39 (t, *J* = 7.6 Hz, 1H), 2.71 (s, 2H), 2.45 (d, *J* = 12.3 Hz, 2H), 2.29 (q, *J* = 4.1 Hz, 1H), 1.87 (s, 1H), 1.82 – 1.60 (m, 9H).

^{13}C-NMR (100 MHz, $CDCl_3$): δ / ppm = 143.8 (q, *J* = 1.5 Hz), 131.9 (q, *J* = 1.5 Hz), 129.4, 129.1 (q, *J* = 7.4 Hz), 127.63 (q, *J* = 29.1 Hz), 127.5, 125.5 (q, *J* = 274 Hz), 37.6, 35.7 (q, *J* = 2.6 Hz), 35.0 (2C), 33.4 (2C), 27.2 (2C), 26.4 (2C).

IR (Diamond-ATR, neat): $\tilde{\nu}$ / cm^{-1} = 3425, 2913, 2876, 2855, 1451, 1443, 1362, 1297, 1284, 1266, 1251, 1185, 1173, 1154, 1124, 1111, 1103, 1097, 1085, 1062, 1051, 1044, 1040, 1030, 1008, 996, 970, 963, 956, 935, 910, 774, 766, 757, 692, 674, 652.

MS (EI, 70 eV): *m/z* (%) = 278 (28), 276 (17), 256 (11), 200 (14), 173 (100), 155 (27), 151 (11), 145 (19), 133 (13), 131 (22), 123 (10), 93 (15), 91 (11), 81 (25), 80 (14), 79 (20).

HRMS (EI-orbitrap): *m/z:* [M] calc. for [$C_{17}H_{19}F_3O$]: 296.1388; found 296.1381.

m.p. (°C): 67.8 – 69.3.

(2-Methoxyphenyl)(4-(trifluoromethyl)phenyl)methanone (6ec)

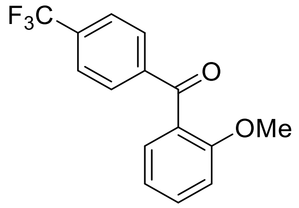

According to **TP2**, solutions of 1-bromo-2-methoxybenzene (**3e**, 0.2 M, 1.0 equiv) in THF, 3-(chloromethyl)heptane (**2**, 0.2 M, 2.0 equiv) in *n*-hexane were prepared. The solution of **2** was pumped through the activated sodium packed-bed reactor (see **TP1**) by pump A (flow rate: 2.0 mL/min) into the precooling loop (V_{pre2} = 0.35 mL) cooled to –40 °C. The solution of **3e** was pumped by pump B (flow rate: 1.0 mL/min) through a precooling loop (V_{pre3} = 2.00 mL) cooled to –40 °C. The precooled solutions were mixed with an overall flow rate of 3.0 mL/min in a T-shaped mixer. The combined stream passed through a tube reactor and a metal needle (V_{R2} = 0.065 mL, t_{R2} = 1.3 s), subsequently upon reaching the steady state, it was injected into a flask charged with N-methoxy-N-methyl-4-(trifluoromethyl)benzamide (**5c**, 70 mg, 0.3 mmol, 1.5 equiv) in THF (1.0 mL) at –20 °C for 60 s. The reaction mixture was stirred at –20 °C for 10 min and allowed to warm to 25 °C before sat. *aq.* NH_4Cl solution was added for quenching the reaction mixture. The aqueous layer was extracted three times with EtOAc (3×30 mL) and the combined organic layers were dried over anhydrous $MgSO_4$ and filtrated. After removal of the solvent, flash column chromatographical purification (silica gel, isohexane:EtOAc = 98:2) afforded the title compound **6ec** as colorless crystals (40 mg, 0.14 mmol, 71% yield).

^{1}H-NMR (400 MHz, $CDCl_3$): δ / ppm = 7.89 (d, *J* = 8.1 Hz, 2H), 7.69 (d, *J* = 8.2 Hz, 2H), 7.57 – 7.47 (m, 1H), 7.42 (dd, *J* = 7.5, 1.7 Hz, 1H), 7.07 (td, *J* = 7.5, 0.8 Hz, 1H), 7.00 (d, *J* = 8.4 Hz, 1H), 3.71 (s, 3H).
^{13}C-NMR (100 MHz, $CDCl_3$): δ / ppm = 195.6, 157.7, 141.0, 134.1 (q, *J* = 32.4 Hz), 132.9, 130.1, 130.0 (2C), 128.0, 125.4 (q, *J* = 3.8 Hz, 2C), 123.9 (q, *J* = 272.5 Hz), 120.9, 111.6, 55.7.
IR (Diamond-ATR, neat): $\tilde{\nu}$ / cm^{-1} = 2923, 2849, 2839, 1673, 1600, 1584, 1510, 1486, 1466, 1454, 1432, 1410, 1325, 1314, 1293, 1262, 1245, 1159, 1149, 1130, 1109, 1063, 1047, 1022, 1017, 983, 942, 925, 862, 772, 754, 709, 701.
MS (EI, 70 eV): *m/z* (%) = 280 (18), 263 (25), 262 (22), 235 (14), 211 (28), 173 (18), 145 (32), 135 (100), 121 (13), 79 (10), 77 (14).
HRMS (EI-orbitrap): *m/z:* [M] calc. for [$C_{15}H_{11}F_3O_2$]: 280.0711; found 280.0706.
m.p. (°C): 77.7 – 83.1.

(2,6-Dichlorophenyl)(2-methoxyphenyl)methanol (6eg)

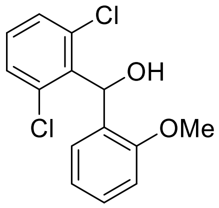

According to **TP2**, solutions of 1-bromo-2-methoxybenzene (**3e**, 0.2 M, 1.0 equiv) in THF, 3-(chloromethyl)heptane (**2**, 0.2 M, 2.0 equiv) in *n*-hexane were prepared. The solution of **2** was pumped through the activated sodium packed-bed reactor (see **TP1**) by pump A (flow rate: 2.0 mL/min) into the precooling loop (V_{pre2} = 0.35 mL) cooled to –40 °C. The solution of **3e** was pumped by pump B (flow rate: 1.0 mL/min) through a precooling loop (V_{pre3} = 2.00 mL) cooled to –40 °C. The precooled solutions were mixed with an overall flow rate of 3.0 mL/min in a T-shaped mixer. The combined stream passed through a tube reactor and a metal needle (V_{R2} = 0.065 mL, t_{R2} = 1.3 s), subsequently upon reaching the steady state, it was injected into a flask charged with 2,6-dichlorobenzaldehyde (**5g**, 70 mg, 0.4 mmol, 2.0 equiv) in THF (1.0 mL) at –20 °C for 60 s. The reaction mixture was stirred at –20 °C for 10 min and allowed to warm to 25 °C before sat. *aq.* NH_4Cl solution was added for quenching the reaction mixture. The aqueous layer was extracted three times with EtOAc (3×30 mL) and the combined organic layers were dried over anhydrous $MgSO_4$ and filtrated. After removal of the solvent, flash column chromatographical purification (silica gel, isohexane:EtOAc = 98:2) afforded the title compound **6eg** as colorless crystals (45 mg, 0.16 mmol, 79% yield).

^{1}H-NMR (400 MHz, $CDCl_3$): δ / ppm = 7.33 – 3.29 (m, 2H), 7.28 – 7.24 (m, 2H), 7.16 (dd, *J* = 8.5, 7.6 Hz, 1H), 6.94 (t, *J* = 7.5 Hz, 1H), 6.88 (d, *J* = 7.9 Hz, 1H), 6.77 (d, *J* = 7.4 Hz, 1H), 3.78 (s, 3H), 3.47 (d, *J* = 7.5 Hz, 1H).

^{13}C-NMR (100 MHz, $CDCl_3$): δ / ppm = 157.0, 137.0, 135.7, 129.4 (2C), 129.1, 128.9, 128.8 (2C), 127.9, 120.2, 110.8, 69.9, 55.5.

IR (Diamond-ATR, neat): $\tilde{\nu}$ / cm^{-1} = 3568, 3478, 2935, 2833, 1600, 1586, 1578, 1562, 1488, 1463, 1456, 1434, 1389, 1308, 1285, 1251, 1236, 1221, 1200, 1181, 1170, 1159, 1149, 1116, 1084, 1075, 1047, 1032, 1026, 1006, 964, 938, 929, 870, 864, 856, 851, 823, 815, 791, 775, 751, 730, 663, 657.

MS (EI, 70 eV): *m/z* (%) = 284 (10), 282 (14), 281 (29), 265 (10), 225 (20), 215 (14), 209 (11), 208 (13), 207 (100), 191 (21), 175 (24), 173 (37), 165 (14), 158 (11), 152 (11), 137 (16), 135 (20), 109 (32), 108 (16), 107 (16), 105 (17), 44 (19).

HRMS (EI-orbitrap): *m/z:* [M] calc. for [$C_{14}H_{12}Cl_2O_2$]: 282.0214; found 282.0210.

m.p. (°C): 133.1 – 137.8.

(4-Methoxy-3,5-dimethylphenyl)(thiophen-2-yl)methanone (6fh)

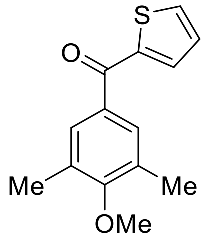

According to **TP2**, solutions of 5-bromo-2-methoxy-1,3-dimethylbenzene (**3f**, 0.2 M, 1.0 equiv) in THF, 3-(chloromethyl)heptane (**2**, 0.2 M, 2.0 equiv) in *n*-hexane were prepared. The solution of **2** was pumped through the activated sodium packed-bed reactor (see **TP1**) by pump A (flow rate: 2.0 mL/min) into the precooling loop (V_{pre2} = 0.35 mL) cooled to –40 °C. The solution of **3f** was pumped by pump B (flow rate: 1.0 mL/min) through a precooling loop (V_{pre3} = 2.00 mL) cooled to –40 °C. The precooled solutions were mixed with an overall flow rate of 3.0 mL/min in a T-shaped mixer. The combined stream passed through a tube reactor and a metal needle (V_{R2} = 0.065 mL, t_{R2} = 1.3 s), subsequently upon reaching the steady state, it was injected into a flask charged with *N*-methoxy-*N*-methylthiophene-2-carboxamide (**5h**, 51 mg, 0.3 mmol, 1.5 equiv) in THF (1.0 mL) at –20 °C for 60 s. The reaction mixture was stirred at –20 °C for 10 min and allowed to warm to 25 °C before sat. *aq.* NH_4Cl solution was added for quenching the reaction mixture. The aqueous layer was extracted three times with EtOAc (3×30 mL) and the combined organic layers were dried over anhydrous $MgSO_4$ and filtrated. After removal of the solvent, flash column chromatographical purification (silica gel, isohexane:EtOAc = 98:2) afforded the title compound **6fh** as a colorless oil (33 mg, 0.13 mmol, 67% yield).

^{1}H-NMR (400 MHz, $CDCl_3$): δ / ppm = 7.70 (dd, *J* = 4.9, 1.0 Hz, 1H), 7.65 (dd, *J* = 3.7, 1.0 Hz, 1H), 7.56 (s, 2H), 7.16 (dd, *J* = 4.9, 3.8 Hz, 1H), 3.79 (s, 3H), 2.35 (s, 6H).

^{13}C-NMR (100 MHz, $CDCl_3$): δ / ppm = 187.8, 160.8, 144.0, 134.6, 133.9, 133.8, 131.3, 130.4 (2C), 128.0 (2C), 59.9, 16.4 (2C).

IR (Diamond-ATR, neat): $\tilde{\nu}$ / cm^{-1} = 2924, 2858, 2827, 1629, 1593, 1513, 1480, 1459, 1451, 1434, 1410, 1379, 1352, 1317, 1244, 1212, 1172, 1116, 1081, 1055, 1004, 960, 950, 896, 862, 825, 794, 770, 764, 743, 719, 698, 664.

MS (EI, 70 eV): *m/z* (%) = 281 (27), 247 (13), 246 (82), 231 (42), 225 (31), 215 (36), 209 (10), 209 (16), 208 (15), 207 (100), 203 (17), 191 (20), 164 (10), 163 (100), 111 (31), 105 (10), 91 (13), 73 (10), 44 (14).

HRMS (EI-orbitrap): *m/z:* [M] calc. for [$C_{14}H_{14}O_2S$]: 246.0715; found 246.0709.

4'-Methoxy-3',5'-dimethyl-1,2,3,4-tetrahydro-1,1'-biphenyl (6fi)

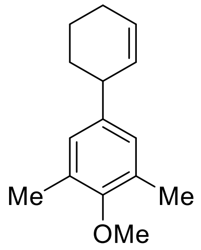

According to **TP2**, solutions of 5-bromo-2-methoxy-1,3-dimethylbenzene (**3f**, 0.2 M, 1.0 equiv) in THF, 3-(chloromethyl)heptane (**2**, 0.2 M, 2.0 equiv) in *n*-hexane were prepared. The solution of **2** was pumped through the activated sodium packed-bed reactor (see **TP1**) by pump A (flow rate: 2.0 mL/min) into the precooling loop (V_{pre2} = 0.35 mL) cooled to –40 °C. The solution of **3f** was pumped by pump B (flow rate: 1.0 mL/min) through a precooling loop (V_{pre3} = 2.00 mL) cooled to –40 °C. The precooled solutions were mixed with an overall flow rate of 3.0 mL/min in a T-shaped mixer. The combined stream passed through a tube reactor and a metal needle (V_{R2} = 0.065 mL, t_{R2} = 1.3 s), subsequently upon reaching the steady state, it was injected into a flask charged with 3-bromocyclohex-1-ene (**5i**, 48 mg, 0.3 mmol, 1.5 equiv) and CuCN·2LiCl (1 M in THF, 0.1 mL, 0.1 mmol, 0.5 equiv) in THF (1.0 mL) at –20 °C for 60 s. The reaction mixture was stirred at –20 °C for 10 min and allowed to warm to 25 °C before sat. *aq.* NH_4Cl solution was added for quenching the reaction mixture. The aqueous layer was extracted three times with EtOAc (3×30 mL) and the combined organic layers were dried over anhydrous $MgSO_4$ and filtrated. After removal of the solvent, flash column chromatographical purification (silica gel, isohexane:EtOAc = 100:0 → 98:2) afforded the title compound **6fi** as a colorless oil (36 mg, 0.17 mmol, 83% yield).

^{1}H-NMR (400 MHz, $CDCl_3$): δ / ppm = 6.85 (s, 2H), 5.92 – 5.81 (m, 1H), 5.72 – 5.65 (m, 1H), 3.71 (s, 3H), 3.35 – 3.23 (m, 1H), 2.27 (s, 6H), 2.12 –2.04 (m, 2H), 2.01 – 1.94 (m, 1H), 1.79 – 1.69 (m, 1H), 1.67 – 1.47 (m, 2H).

^{13}C-NMR (100 MHz, $CDCl_3$): δ / ppm = 155.1, 141.9, 130.6 (2C), 130.5, 128.1, 128.0 (2C), 59.7, 41.2, 32.7, 25.0, 21.3, 16.1 (2C).

IR (Diamond-ATR, neat): $\tilde{\nu}$ / cm^{-1} = 2928, 2924, 2853, 1716, 1651, 1598, 1482, 1455, 1375, 1306, 1220, 1215, 1154, 1147, 1010, 865, 862.

MS (EI, 70 eV): *m/z* (%) = 216 (79), 215 (28), 213 (27), 202 (25), 201 (69), 185 (25), 178 (85), 174 (23), 173 (58), 163 (100), 162 (21), 159 (32), 157 (21), 149 (38), 141 (21), 136 (33), 135 (69), 129 (25), 128 (32), 119 (19), 115 (36), 105 (53), 103 (19), 91 (61), 81 (35), 79 (34), 77 (40), 57 (24), 42 (20), 41 (68).

HRMS (EI-orbitrap): *m/z:* [M] calc. for [$C_{15}H_{20}O$]: 216.1514.; found 216.1503.

Cyclohexyl(4-methoxy-3,5-dimethylphenyl)(phenyl)methanol (6fj)

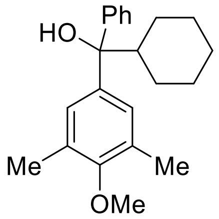

According to **TP2**, solutions of 5-bromo-2-methoxy-1,3-dimethylbenzene (**3f**, 0.2 M, 1.0 equiv) in THF, 3-(chloromethyl)heptane (**2**, 0.2 M, 2.0 equiv) in *n*-hexane were prepared. The solution of **2** was pumped through the activated sodium packed-bed reactor (see **TP1**) by pump A (flow rate: 2.0 mL/min) into the precooling loop (V_{pre2} = 0.35 mL) cooled to –40 °C. The solution of **3f** was pumped by pump B (flow rate: 1.0 mL/min) through a precooling loop (V_{pre3} = 2.00 mL) cooled to –40 °C. The precooled solutions were mixed with an overall flow rate of 3.0 mL/min in a T-shaped mixer. The combined stream passed through a tube reactor and a metal needle (V_{R2} = 0.065 mL, t_{R2} = 1.3 s), subsequently upon reaching the steady state, it was injected into a flask charged with cyclohexyl(phenyl)methanone (**5j**, 56 mg, 0.3 mmol, 1.5 equiv) in THF (1.0 mL) at –20 °C for 60 s. The reaction mixture was stirred at –20 °C for 10 min and allowed to warm to 25 °C before sat. *aq.* NH_4Cl solution was added for quenching the reaction mixture. The aqueous layer was extracted three times with EtOAc (3×30 mL) and the combined organic layers were dried over anhydrous $MgSO_4$ and filtrated. After removal of the solvent, flash column chromatographical purification (silica gel, isohexane:EtOAc = 98:2 → 97:3) afforded the title compound **6fj** as a colorless oil (62 mg, 0.19 mmol, 95% yield).

^{1}H-NMR (400 MHz, $CDCl_3$): δ / ppm = 7.51 7.47 (m, 2H), 7.30 (t, *J* = 7.7 Hz, 2H), 7.21 – 7.14 (m, 1H), 7.11 (s, 2H), 3.67 (s, 3H), 2.45 – 2.34 (m, 1H), 2.25 (s, 6H), 2.06 (s, 1H), 1.81 1.61 (m, 4H), 1.51 (d, *J* = 12.3 Hz, 1H), 1.38 – 1.24 (m, 2H), 1.19 – 1.01 (m, 3H).

^{13}C-NMR (100 MHz, $CDCl_3$): δ / ppm = 155.4, 146.8, 141.6, 130.3 (2C), 128.1 (2C), 126.3, 126.2 (2C), 125.8 (2C), 80.2, 59.7, 45.8, 27.3 (2C), 26.8 (2C), 26.7, 16.6 (2C).

IR (Diamond-ATR, neat): $\tilde{\nu}$ / cm^{-1} = 3486, 2928, 2850, 2824, 1598, 1484, 1445, 1416, 1373, 1353, 1334, 1306, 1300, 1276, 1221, 1190, 1157, 1146, 1127, 1080, 1069, 1012, 977, 943, 909, 896, 868, 852, 825, 803, 765, 757, 728, 699, 676, 665, 656.

MS (EI, 70 eV): *m/z* (%) = 242 (17), 241 (32), 241 (100), 241 (70), 105 (69).

HRMS (EI-orbitrap): *m/z:* [M] calc. for [$C_{22}H_{28}O_2$]: 324.2089; found 324.2087.

(2-(Dimethylamino)phenyl)(phenyl)(pyridin-4-yl)methanol (6gk)

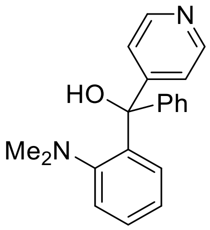

According to **TP2**, solutions of 2-bromo-*N,N*-dimethylaniline (**3g**, 0.2 M, 1.0 equiv) in THF, 3-(chloromethyl)heptane (**2**, 0.2 M, 2.0 equiv) in *n*-hexane were prepared. The solution of **2** was pumped through the activated sodium packed-bed reactor (see **TP1**) by pump A (flow rate: 2.0 mL/min) into the precooling loop (V_{pre2} = 0.35 mL) cooled to –40 °C. The solution of **3g** was pumped by pump B (flow rate: 1.0 mL/min) through a precooling loop (V_{pre3} = 2.00 mL) cooled to –40 °C. The precooled solutions were mixed with an overall flow rate of 3.0 mL/min in a T-shaped mixer. The combined stream passed through a tube reactor and a metal needle (V_{R2} = 0.065 mL, t_{R2} = 1.3 s), subsequently upon reaching the steady state, it was injected into a flask charged with phenyl(pyridin-4-yl)methanone (**5k**, 55 mg, 0.3 mmol, 1.5 equiv) in THF (1.0 mL) at –20 °C for 60 s. The reaction mixture was stirred at –20 °C for 10 min and allowed to warm to 25 °C before sat. *aq.* NH_4Cl solution was added for quenching the reaction mixture. The aqueous layer was extracted three times with EtOAc (3×30 mL) and the combined organic layers were dried over anhydrous $MgSO_4$ and filtrated. After removal of the solvent, flash column chromatographical purification (silica gel, isohexane:EtOAc = 99:1 → 85:15) afforded the title compound **6gk** as colorless crystals (52 mg, 0.17 mmol, 85% yield).

^{1}H-NMR (400 MHz, $CDCl_3$): δ / ppm = 10.03 (s, 1H), 8.58 – 8.46 (m, 2H), 7.36 (dd, *J* = 8.1, 1.4 Hz, 1H), 7.32 – 7.18 (m, 8H), 7.05 (td, *J* = 7.5, 1.5 Hz, 1H), 6.65 (dd, *J* = 7.8, 1.6 Hz, 1H), 2.36 (s, 6H).

^{13}C-NMR (100 MHz, $CDCl_3$): δ / ppm = 156.5, 152.1, 149.6 (2C), 146.2, 141.3, 130.3, 128.9, 128.1 (2C), 128.1 (2C), 127.5, 125.6, 123.9, 123.3 (2C), 82.4, 45.8 (2C).

IR (Diamond-ATR, neat): $\tilde{\nu}$ / cm^{-1} = 3058, 3021, 2982, 2947, 2863, 2832, 2789, 1734, 1591, 1577, 1553, 1483, 1459, 1446, 1432, 1405, 1373, 1323, 1283, 1266, 1242, 1220, 1198, 1174, 1147, 1098, 1068, 1055, 1037, 1001, 993, 939, 931, 922, 903, 816, 766, 755, 733, 700, 667.

MS (EI, 70 eV): *m/z* (%) = 304 (17), 227 (53), 226 (85), 212 (16), 211 (100), 210 (30), 209 (30), 208 (58), 195 (24), 194 (18), 193 (38), 184 (17), 167 (27), 165 (16), 152 (16), 136 (66), 120 (55), 118 (20), 106 (22), 96 (19), 91 (97), 78 (23), 77 (48).

HRMS (EI-orbitrap): *m/z:* [M] calc. for [$C_{20}H_{20}N_2O$]: 304.1576; found 304.1567.

m.p. (°C): 109.8 – 111.5.

***N,N*-Dimethyl-2-(phenyl(phenylamino)methyl)aniline (6gd)**

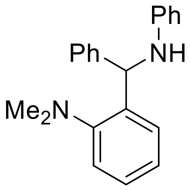

According to **TP2**, solutions of 2-bromo-*N,N*-dimethylaniline (**3g**, 0.2 M, 1.0 equiv) in THF, 3-(chloromethyl)heptane (**2**, 0.2 M, 2.0 equiv) in *n*-hexane were prepared. The solution of **2** was pumped through the activated sodium packed-bed reactor (see **TP1**) by pump A (flow rate: 2.0 mL/min) into the precooling loop (V_{pre2} = 0.35 mL) cooled to –40 °C. The solution of **3g** was pumped by pump B (flow rate: 1.0 mL/min) through a precooling loop (V_{pre3} = 2.00 mL) cooled to –40 °C. The precooled solutions were mixed with an overall flow rate of 3.0 mL/min in a T-shaped mixer. The combined stream passed through a tube reactor and a metal needle (V_{R2} = 0.065 mL, t_{R2} = 1.3 s), subsequently upon reaching the steady state, it was injected into a flask charged with *N*,1-diphenylmethanimine (**5d**, 54 mg, 0.3 mmol, 1.5 equiv) in THF (1.0 mL) at –20 °C for 60 s. The reaction mixture was stirred at –20 °C for 10 min and allowed to warm to 25 °C before sat. *aq.* NH_4Cl solution was added for quenching the reaction mixture. The aqueous layer was extracted three times with EtOAc (3×30 mL) and the combined organic layers were dried over anhydrous $MgSO_4$ and filtrated. After removal of the solvent, flash column chromatographical purification (silica gel, isohexane:EtOAc = 99:1 → 98:2) afforded the title compound **6gd** as a colorless crystals (48 mg, 0.16 mmol, 79% yield).

^{1}H-NMR (400 MHz, $CDCl_3$): δ / ppm = 7.30 – 7.25 (m, 2H), 7.22 – 7.08 (m, 6H), 7.04 – 6.98 (m, 2H), 6.98 – 6.93 (m, 1H), 6.56 (tt, *J* = 7.3, 1.1 Hz, 1H), 6.51 – 6.44 (m, 2H), 6.08 (s, 1H), 4.33 (s, 1H), 2.53 (s, 6H).
^{13}C-NMR (100 MHz, $CDCl_3$): δ / ppm = 152.5, 147.7, 143.6, 138.4, 129.2 (2C), 128.9, 128.5 (2C), 128.2, 127.6 (2C), 126.9, 124.5, 120.8, 117.3, 113.3 (2C), 56.3, 45.7 (2C).
IR (Diamond-ATR, neat): $\tilde{\nu}$ / cm^{-1} = 3414, 3022, 2973, 2940, 2926, 2855, 2825, 2785, 1600, 1583, 1504, 1488, 1477, 1459, 1447, 1429, 1406, 1351, 1313, 1299, 1266, 1237, 1197, 1183, 1178, 1164, 1153, 1119, 1107, 1098, 1083, 1078, 1064, 1045, 1027, 991, 985, 944, 888, 866, 847, 814, 770, 757, 749, 742, 730, 692.
MS (EI, 70 eV): *m/z* (%) = 302 (42), 287 (13), 211 (21), 210 (100), 209 (39), 208 (28), 194 (26), 180 (10), 165 (16), 132 (13), 118 (15), 91 (44), 77 (15), 44 (12).
HRMS (EI-orbitrap): *m/z:* [M] calc. for [$C_{21}H_{22}N_2$]: 302.1783; found 302.1775.
m.p. (°C): 128.7 – 129.8.

***N,N*-Dimethyl-2-(pyridin-2-ylthio)aniline (6gl)**

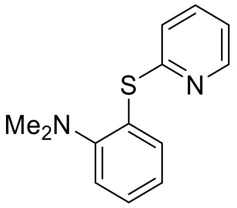

According to **TP2**, solutions of 2-bromo-*N,N*-dimethylaniline (**3g**, 0.2 M, 1.0 equiv) in THF, 3-(chloromethyl)heptane (**2**, 0.2 M, 2.0 equiv) in *n*-hexane were prepared. The solution of **2** was pumped through the activated sodium packed-bed reactor (see **TP1**) by pump A (flow rate: 2.0 mL/min) into the precooling loop (V_{pre2} = 0.35 mL) cooled to –40 °C. The solution of **3g** was pumped by pump B (flow rate: 1.0 mL/min) through a precooling loop (V_{pre3} = 2.00 mL) cooled to –40 °C. The precooled solutions were mixed with an overall flow rate of 3.0 mL/min in a T-shaped mixer. The combined stream passed through a tube reactor and a metal needle (V_{R2} = 0.065 mL, t_{R2} = 1.3 s), subsequently upon reaching the steady state, it was injected into a flask charged with aldrithiol (**5l**, 66 mg, 0.3 mmol, 1.5 equiv) in THF (1.0 mL) at –20 °C for 60 s. The reaction mixture was stirred at –20 °C for 10 min and allowed to warm to 25 °C before sat. *aq.* NH_4Cl solution was added for quenching the reaction mixture. The aqueous layer was extracted three times with EtOAc (3×30 mL) and the combined organic layers were dried over anhydrous $MgSO_4$ and filtrated. After removal of the solvent, flash column chromatographical purification (silica gel, isohexane:EtOAc = 9:1) afforded the title compound **6gl** as a colorless oil (41 mg, 0.18 mmol, 89% yield).

^{1}H-NMR (400 MHz, $CDCl_3$): δ / ppm = 8.45 – 8.42 (m, 1H), 7.50 – 7.40 (m, 2H), 7.36 – 7.30 (m, 1H), 7.13 (dd, *J* = 8.1, 1.4 Hz, 1H), 7.03 – 6.96 (m, 2H), 6.86 (dt, *J* = 8.2, 1.0 Hz, 1H), 2.80 (s, 6H).

^{13}C-NMR (100 MHz, $CDCl_3$): δ / ppm = 161.3, 155.3, 149.6, 136.7, 136.6, 130.0, 125.6, 123.3, 122.1, 120.0, 119.8, 44.6 (2C).

IR (Diamond-ATR, neat): $\tilde{\nu}$ / cm^{-1} = 3052, 3041, 2936, 2827, 2779, 1582, 1572, 1558, 1478, 1445, 1427, 1415, 1316, 1278, 1266, 1188, 1158, 1143, 1117, 1095, 1085, 1057, 1041, 984, 944, 874, 754, 738, 721, 670.

MS (EI, 70 eV): *m/z* (%) = 197 (25), 186 (22), 150 (49), 136 (29), 109 (17), 93 (100), 91 (18), 80 (13).

HRMS (EI-orbitrap): *m/z:* [M] calc. for [$C_{13}H_{14}N_2S$]: 230.0878; found 230.0874.

Diphenyl(pyridin-2-yl)methanol (9aa)

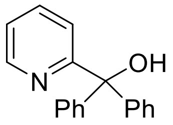

According to **TP2**, solutions of 2-bromopyridine (**7a**, 0.2 M, 1.0 equiv) in THF, 3-(chloromethyl)heptane (**2**, 0.2 M, 2.0 equiv) in *n*-hexane were prepared. The solution of **2** was pumped through the activated sodium packed-bed reactor (see **TP1**) by pump A (flow rate: 2.0 mL/min) into the precooling loop (V_{pre2} = 0.35 mL) cooled to –40 °C. The solution of **7a** was pumped by pump B (flow rate: 1.0 mL/min) through a precooling loop (V_{pre3} = 2.00 mL) cooled to –40 °C. The precooled solutions were mixed with an overall flow rate of 3.0 mL/min in a T-shaped mixer. The combined stream passed through a tube reactor and a metal needle (V_{R2} = 0.065 mL, t_{R2} = 1.3 s), subsequently upon reaching the steady state, it was injected into a flask charged with benzophenone (**5a**, 55 mg, 0.3 mmol, 1.5 equiv) in THF (1.0 mL) at –20 °C for 60 s. The reaction mixture was stirred at –20 °C for 10 min and allowed to warm to 25 °C before sat. *aq.* NH_4Cl solution was added for quenching the reaction mixture. The aqueous layer was extracted three times with EtOAc (3×30 mL) and the combined organic layers were dried over anhydrous $MgSO_4$ and filtrated. After removal of the solvent, flash column chromatographical purification (silica gel, isohexane:EtOAc = 95:5 → 9:1) afforded the title compound **9aa** as colorless crystals (45 mg, 0.17 mmol, 86% yield).

^{1}H-NMR (400 MHz, $CDCl_3$): δ / ppm = 8.60 (dt, *J* = 4.8, 1.4 Hz, 1H), 7.64 (td, *J* = 7.7, 1.8 Hz, 1H), 7.35 – 7.21 (m, 11H), 7.12 (dt, *J* = 8.0, 1.1 Hz, 1H), 6.30 (s, 1H).

^{13}C-NMR (100 MHz, $CDCl_3$): δ / ppm = 163.3, 147.9, 146.2 (2C), 136.6, 128.3 (4C), 128.1 (4C), 127.5 (2C), 123.1, 122.5, 81.0.

IR (Diamond-ATR, neat): $\tilde{\nu}$ / cm^{-1} = 3341, 3334, 3070, 3054, 3029, 3017, 2923, 2896, 2852, 1591, 1571, 1487, 1466, 1445, 1437, 1379, 1294, 1255, 1210, 1201, 1178, 1167, 1155, 1103, 1088, 1077, 1038, 1026, 998, 987, 971, 940, 930, 913, 896, 852, 784, 766, 761, 756, 715, 697, 655.

MS (EI, 70 eV): *m/z* (%) = 262 (11), 261 (54), 260 (24), 244 (15), 243 (100), 242 (24), 241 (34), 240 (11), 207 (23), 184 (56), 167 (14), 165 (17), 156 (10), 120 (13), 106 (17), 105 (30), 78 (17), 77 (16).

HRMS (EI-orbitrap): *m/z:* [M] calc. for [$C_{18}H_{15}NO$]: 261.1154; found 261.1150.

m.p. (°C): 104.6 – 108.2.

(4-Chlorophenyl)(cyclopropyl)(pyridin-2-yl)methanol (9am)

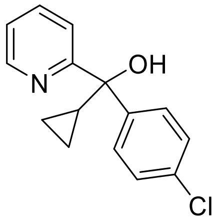

According to **TP2**, solutions of 2-bromopyridine (**7a**, 0.2 M, 1.0 equiv) in THF, 3-(chloromethyl)heptane (**2**, 0.2 M, 2.0 equiv) in *n*-hexane were prepared. The solution of **2** was pumped through the activated sodium packed-bed reactor (see **TP1**) by pump A (flow rate: 2.0 mL/min) into the precooling loop (V_{pre2} = 0.35 mL) cooled to –40 °C. The solution of **7a** was pumped by pump B (flow rate: 1.0 mL/min) through a precooling loop (V_{pre3} = 2.00 mL) cooled to –40 °C. The precooled solutions were mixed with an overall flow rate of 3.0 mL/min in a T-shaped mixer. The combined stream passed through a tube reactor and a metal needle (V_{R2} = 0.065 mL, t_{R2} = 1.3 s), subsequently upon reaching the steady state, it was injected into a flask charged with (4-chlorophenyl)(cyclopropyl)methanone (**5m**, 54 mg, 0.3 mmol, 1.5 equiv) in THF (1.0 mL) at –20 °C for 60 s. The reaction mixture was stirred at –20 °C for 10 min and allowed to warm to 25 °C before sat. *aq.* NH_4Cl solution was added for quenching the reaction mixture. The aqueous layer was extracted three times with EtOAc (3×30 mL) and the combined organic layers were dried over anhydrous $MgSO_4$ and filtrated. After removal of the solvent, flash column chromatographical purification (silica gel, isohexane:EtOAc = 95:5 → 9:1) afforded the title compound **9am** as a colorless oil (42 mg, 0.16 mmol, 81% yield).

^{1}H-NMR (400 MHz, CDCl$_3$): δ / ppm = 8.43 (d, *J* = 4.9 Hz, 1H), 7.59 (td, *J* = 7.7, 1.8 Hz, 1H), 7.46 – 7.36 (m, 2H), 7.23 – 7.16 (m, 3H), 7.16 – 7.12 (m, 1H), 5.70 (s, 1H), 1.60 – 1.47 (m, 1H), 0.60 – 0.49 (m, 2H), 0.44 – 0.36 (m, 1H), 0.34 – 0.26 (m, 1H).

^{13}C-NMR (100 MHz, CDCl$_3$): δ / ppm = 163.8, 147.1, 145.3, 137.1, 133.1, 128.7 (2C), 128.3 (2C), 122.4, 121.2, 75.0, 20.5, 1.9, 0.8.

IR (Diamond-ATR, neat): $\tilde{\nu}$ / cm^{-1} = 3433, 3340, 3085, 3007, 1591, 1570, 1488, 1468, 1432, 1397, 1355, 1302, 1294, 1210, 1192, 1151, 1090, 1047, 1014, 994, 965, 958, 884, 872, 824, 785, 771, 748, 733, 719, 683.

MS (EI, 70 eV): *m/z* (%) = 260 (12), 259 (12), 258 (37), 244 (20), 240 (22), 230 (28), 220 (13), 218 (40), 204 (12), 190 (10), 167 (13), 148 (14), 141 (21), 139 (10), 139 (64), 134 (63), 132 (19), 125 (11), 111 (12), 106 (45), 93 (20), 79 (37), 78 (100).

HRMS (EI-orbitrap): *m/z:* [M] calc. for [$C_{15}H_{14}ClNO$]: 259.0765; found 259.0768.

(5-Methylpyridin-2-yl)(4-(trifluoromethyl)phenyl)methanone (9bc)

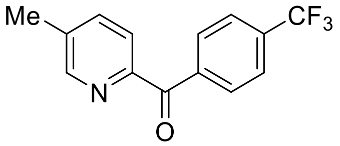

According to **TP2**, solutions of 2-bromo-5-methylpyridine (**7b**, 0.2 M, 1.0 equiv) in THF, 3-(chloromethyl)heptane (**2**, 0.2 M, 2.0 equiv) in *n*-hexane were prepared. The solution of **2** was pumped through the activated sodium packed-bed reactor (see **TP1**) by pump A (flow rate: 2.0 mL/min) into the precooling loop (V_{pre2} = 0.35 mL) cooled to –40 °C. The solution of **7b** was pumped by pump B (flow rate: 1.0 mL/min) through a precooling loop (V_{pre3} = 2.00 mL) cooled to –40 °C. The precooled solutions were mixed with an overall flow rate of 3.0 mL/min in a T-shaped mixer. The combined stream passed through a tube reactor and a metal needle (V_{R2} = 0.065 mL, t_{R2} = 1.3 s), subsequently upon reaching the steady state, it was injected into a flask charged with *N*-methoxy-*N*-methyl-4-(trifluoromethyl)benzamide (**5c**, 70 mg, 0.3 mmol, 1.5 equiv) in THF (1.0 mL) at –20 °C for 60 s. The reaction mixture was stirred at –20 °C for 10 min and allowed to warm to 25 °C before sat. *aq.* NH_4Cl solution was added for quenching the reaction mixture. The aqueous layer was extracted three times with EtOAc (3×30 mL) and the combined organic layers were dried over anhydrous $MgSO_4$ and filtrated. After removal of the solvent, flash column chromatographical purification (silica gel, isohexane:EtOAc = 95:5 → 9:1 → 7:3) afforded the title compound **9bc** as colorless crystals (44 mg, 0.17 mmol, 83% yield).

^{1}H-NMR (600 MHz, $CDCl_3$): δ / ppm = 8.56 – 8.52 (m, 1H), 8.17 (d, *J* = 8.1 Hz, 2H), 8.06 (d, *J* = 8.0 Hz, 1H), 7.76 – 7.72 (m, 3H), 2.47 (s, 3H).

^{13}C-NMR (150 MHz, $CDCl_3$): δ / ppm = 192.8, 151.8, 149.3, 139.8 (q, *J* = 1.3 Hz, 2C), 137.8, 137.5, 133.9 (q, *J* = 32.6 Hz), 131.3, 125.2 (q, *J* = 3.8 Hz, 2C), 124.7, 123.9 (q, *J* = 271.9 Hz), 18.9.

IR (Diamond-ATR, neat): $\tilde{\nu}$ / cm^{-1} = 2923, 2852, 1668, 1643, 1582, 1567, 1510, 1407, 1383, 1325, 1312, 1307, 1292, 1245, 1220, 1188, 1158, 1108, 1063, 1028, 1016, 975, 968, 935, 858, 849, 836, 814, 805, 779, 773, 746, 702, 683, 652.

MS (EI, 70 eV): *m/z* (%) = 265 (43), 264 (69), 238 (16), 237 (100), 236 (51), 207 (11), 196 (17), 173 (51), 170 (13), 146 (74), 92 (14), 65 (16), 57 (12), 45 (10), 44 (37), 43 (11).

HRMS (EI-orbitrap): *m/z:* [M] calc. for [$C_{14}H_{10}F_3NO$]: 265.0714; found 265.0709.

m.p. (°C): 61.4 – 64.5.

(2,6-Dichlorophenyl)(2,4-dimethoxypyrimidin-5-yl)methanol (9cg)

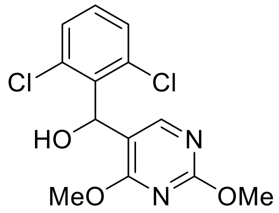

According to **TP2**, solutions of 5-bromo-2,4-dimethoxypyrimidine (**7c**, 0.2 M, 1.0 equiv) in THF, 3-(chloromethyl)heptane (**2**, 0.2 M, 2.0 equiv) in *n*-hexane were prepared. The solution of **2** was pumped through the activated sodium packed-bed reactor (see **TP1**) by pump A (flow rate: 2.0 mL/min) into the precooling loop (V_{pre2} = 0.35 mL) cooled to –40 °C. The solution of **7c** was pumped by pump B (flow rate: 1.0 mL/min) through a precooling loop (V_{pre3} = 2.00 mL) cooled to –40 °C. The precooled solutions were mixed with an overall flow rate of 3.0 mL/min in a T-shaped mixer. The combined stream passed through a tube reactor and a metal needle (V_{R2} = 0.065 mL, t_{R2} = 1.3 s), subsequently upon reaching the steady state, it was injected into a flask charged with 2,6-dichlorobenzaldehyde (**5g**, 70 mg, 0.4 mmol, 2.0 equiv) in THF (1.0 mL) at –20 °C for 60 s. The reaction mixture was stirred at –20 °C for 10 min and allowed to warm to 25 °C before sat. *aq.* NH_4Cl solution was added for quenching the reaction mixture. The aqueous layer was extracted three times with EtOAc (3×30 mL) and the combined organic layers were dried over anhydrous $MgSO_4$ and filtrated. After removal of the solvent, flash column chromatographical purification (silica gel, isohexane:EtOAc = 7:3 → 1:1) afforded the title compound **9cg** as colorless crystals (54 mg, 0.17 mmol, 86% yield).

According to **TP4**, sodium dispersion (30 wt% in toluene, particle size <0.1 mm, 1.05 mmol, 4.2 equiv) was added into a flame dried round bottom flask charged with a stirring bar, the toluene was removed *in vacuo* n-hexane (2.0 mL) was added. Neopentyl chloride was added (53 mg, 0.50 mmol, 2.0 equiv) at 0 °C. The mixture was stirred at 0 °C for 20 min before **7c** (55 mg, 0.25 mmol, 1.0 equiv) was added at 0 °C and the mixture was again stirred at this temperature for 30 min. **5g** (53 mg, 0.30 mmol, 1.2 equiv) was added at 0 °C the mixture was allowed to warm to 25 °C and stirred for 30 min before it was quenched with NH_4Cl. In our hands no product **9cg** was detected on GCMS.

^{1}H-NMR (400 MHz, $CDCl_3$): δ / ppm = 8.23 (s, 1H), 7.35 – 7.29 (m, 2H), 7.19 (dd, *J* = 8.6, 7.4 Hz, 1H), 6.63 – 6.55 (m, 1H), 3.98 (s, 3H), 3.93 (s, 3H), 3.31 (d, *J* = 8.1 Hz, 1H).

^{13}C-NMR (100 MHz, $CDCl_3$): δ / ppm = 168.4, 165.0, 156.8, 135.4, 135.3 (2C), 129.7, 129.5 (2C), 114.0, 67.4, 55.0, 54.1.

IR (Diamond-ATR, neat): $\tilde{\nu}$ / cm^{-1} = 3267, 3005, 2953, 1598, 1558, 1480, 1462, 1433, 1399, 1382, 1354, 1285, 1263, 1225, 1193, 1175, 1163, 1091, 1081, 1072, 1042, 1015, 833, 792, 787, 780, 764, 728, 690, 675, 655.

MS (EI, 70 eV): *m/z* (%) = 225 (16), 209 (10), 207 (44), 191 (13), 175 (18), 173 (28), 169 (100), 167 (16), 141 (43), 109 (12), 85 (20), 84 (11), 75 (18), 73 (19).

HRMS (EI-orbitrap): *m/z:* [M] calc. for [$C_{13}H_{12}Cl_2N_2O_3$]: 314.0225; found 314.0223.

m.p. (°C): 140.6-145.6.

1-(2,4-Dimethoxypyrimidin-5-yl)-1-phenylethan-1-ol (9cn)

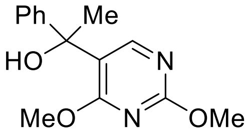

According to **TP2**, solutions of 5-bromo-2,4-dimethoxypyrimidine (**7c**, 0.2 M, 1.0 equiv) in THF, 3-(chloromethyl)heptane (**2**, 0.2 M, 2.0 equiv) in *n*-hexane were prepared. The solution of **2** was pumped through the activated sodium packed-bed reactor (see **TP1**) by pump A (flow rate: 2.0 mL/min) into the precooling loop (V_{pre2} = 0.35 mL) cooled to –40 °C. The solution of **7c** was pumped by pump B (flow rate: 1.0 mL/min) through a precooling loop (V_{pre3} = 2.00 mL) cooled to –40 °C. The precooled solutions were mixed with an overall flow rate of 3.0 mL/min in a T-shaped mixer. The combined stream passed through a tube reactor and a metal needle (V_{R2} = 0.065 mL, t_{R2} = 1.3 s), subsequently upon reaching the steady state, it was injected into a flask charged with acetophenone (**5n**, 36 mg, 0.3 mmol, 1.5 equiv) in THF (1.0 mL) at –20 °C for 60 s. The reaction mixture was stirred at –20 °C for 10 min and allowed to warm to 25 °C before sat. *aq.* NH_4Cl solution was added for quenching the reaction mixture. The aqueous layer was extracted three times with EtOAc (3×30 mL) and the combined organic layers were dried over anhydrous $MgSO_4$ and filtrated. After removal of the solvent, flash column chromatographical purification (silica gel, isohexane:EtOAc = 1:1 → 4:6) afforded the title compound **9cn** as a colorless oil (50 mg, 0.19 mmol, 96% yield).

^{1}H-NMR (400 MHz, $CDCl_3$): δ / ppm = 8.31 (s, 1H), 7.33 – 7.20 (m, 5H), 4.00 (s, 3H), 3.86 (s, 3H), 3.71 (s, 1H), 1.85 (s, 3H).

^{13}C-NMR (100 MHz, $CDCl_3$): δ / ppm = 168.4, 164.8, 155.4, 147.3, 128.3 (2C), 127.2, 125.0 (2C), 120.5, 74.0, 55.0, 54.3, 29.3.

IR (Diamond-ATR, neat): $\tilde{\nu}$ / cm^{-1} = 2981, 2954, 1591, 1560, 1467, 1455, 1381, 1320, 1288, 1277, 1237, 1221, 1197, 1119, 1103, 1085, 1059, 1037, 1026, 1011, 964, 938, 922, 908, 832, 801, 765, 739, 697.

MS (EI, 70 eV): *m/z* (%) = 246 (15). 245 (100), 242 (10). 183 (28), 167 (22), 105 (11), 77 (10), 43 (12).

HRMS (EI-orbitrap): *m/z:* [M] calc. for $[C_{14}H_{16}N_2O_3]$: 260.1161; found 260.1155.

2,4-Dimethoxy-5-(pyridin-2-ylthio)pyrimidine (9cc)

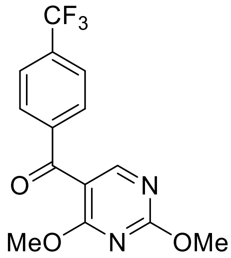

According to **TP2**, solutions of 5-bromo-2,4-dimethoxypyrimidine (**7c**, 0.2 M, 1.0 equiv) in THF, 3-(chloromethyl)heptane (**2**, 0.2 M, 2.0 equiv) in *n*-hexane were prepared. The solution of **2** was pumped through the activated sodium packed-bed reactor (see **TP1**) by pump A (flow rate: 2.0 mL/min) into the precooling loop (V_{pre2} = 0.35 mL) cooled to –40 °C. The solution of **7c** was pumped by pump B (flow rate: 1.0 mL/min) through a precooling loop (V_{pre3} = 2.00 mL) cooled to –40 °C. The precooled solutions were mixed with an overall flow rate of 3.0 mL/min in a T-shaped mixer. The combined stream passed through a tube reactor and a metal needle (V_{R2} = 0.065 mL, t_{R2} = 1.3 s), subsequently upon reaching the steady state, it was injected into a flask charged with *N*-methoxy-*N*-methyl-4-(trifluoromethyl)benzamide (**5c**, 70 mg, 0.3 mmol, 1.5 equiv) in THF (1.0 mL) at –20 °C for 60 s. The reaction mixture was stirred at –20 °C for 10 min and allowed to warm to 25 °C before sat. *aq.* NH_4Cl solution was added for quenching the reaction mixture. The aqueous layer was extracted three times with EtOAc (3×30 mL) and the combined organic layers were dried over anhydrous $MgSO_4$ and filtrated. After removal of the solvent, flash column chromatographical purification (silica gel, isohexane:EtOAc = 8:2 → 7:3) afforded the title compound **9cc** as colorless crystals (49 mg, 0.16 mmol, 78% yield).

^{1}H-NMR (400 MHz, $CDCl_3$): δ / ppm = 8.53 (s, 1H), 7.84 (d, *J* = 8.2 Hz, 2H), 7.72 (d, *J* = 8.2 Hz, 2H), 4.09 (s, 3H), 3.95 (s, 3H).

^{13}C-NMR (100 MHz, $CDCl_3$): δ / ppm = 191.3, 169.3, 166.8, 162.0 (2C), 140.8, 134.4 (q, *J* = 32.7 Hz), 129.7 (2C), 125.6 (q, *J* = 3.7 Hz), 121.0 (q, *J* = 273.1 Hz), 113.9, 55.7, 54.6.

IR (Diamond-ATR, neat): $\tilde{\nu}$ / cm^{-1} = 2961, 2923, 2851, 1661, 1585, 1555, 1510, 1471, 1462, 1398, 1389, 1320, 1310, 1294, 1266, 1251, 1242, 1189, 1162, 1148, 1127, 1102, 1061, 1005, 985, 960, 935, 918, 860, 841, 800, 780, 771, 767, 740, 713, 702, 666.

MS (EI, 70 eV): *m/z* (%) = 313 (11), 312 (88), 311 (22), 282 (25), 173 (35), 167 (100), 145 (49), 44 (14), 42 (26).

HRMS (EI-orbitrap): *m/z:* [M] calc. for [$C_{14}H_{11}F_3N_2O_3$]: 312.0722; found 312.0716.

m.p. (°C): 98.2 – 99.9.

2,4-Dimethoxy-5-(pyridin-2-ylthio)pyrimidine (9cl)

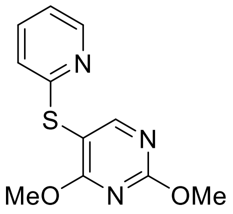

According to **TP2**, solutions of 5-bromo-2,4-dimethoxypyrimidine (**7c**, 0.2 M, 1.0 equiv) in THF, 3-(chloromethyl)heptane (**2**, 0.2 M, 2.0 equiv) in *n*-hexane were prepared. The solution of **2** was pumped through the activated sodium packed-bed reactor (see **TP1**) by pump A (flow rate: 2.0 mL/min) into the precooling loop (V_{pre2} = 0.35 mL) cooled to –40 °C. The solution of **7c** was pumped by pump B (flow rate: 1.0 mL/min) through a precooling loop (V_{pre3} = 2.00 mL) cooled to –40 °C. The precooled solutions were mixed with an overall flow rate of 3.0 mL/min in a T-shaped mixer. The combined stream passed through a tube reactor and a metal needle (V_{R2} = 0.065 mL, t_{R2} = 1.3 s), subsequently upon reaching the steady state, it was injected into a flask charged with aldrithiol (**5l**, 66 mg, 0.3 mmol, 1.5 equiv) in THF (1.0 mL) at –20 °C for 60 s. The reaction mixture was stirred at –20 °C for 10 min and allowed to warm to 25 °C before sat. *aq.* NH_4Cl solution was added for quenching the reaction mixture. The aqueous layer was extracted three times with EtOAc (3×30 mL) and the combined organic layers were dried over anhydrous $MgSO_4$ and filtrated. After removal of the solvent, flash column chromatographical purification (silica gel, isohexane:EtOAc = 1:1 → 4:6) afforded the title compound **9cl** as colorless crystals (45 mg, 0.18 mmol, 90% yield).

^{1}H-NMR (400 MHz, $CDCl_3$): δ / ppm = 8.44 (s, 1H), 8.40 – 8.33 (m, 1H), 7.49 (td, *J* = 7.8, 1.9 Hz, 1H), 7.04 – 6.98 (m, 1H), 6.95 (d, *J* = 8.1 Hz, 1H), 4.05 (s, 3H), 3.98 (s, 3H).

^{13}C-NMR (100 MHz, $CDCl_3$): δ / ppm = 171.1, 166.4, 165.3, 158.9, 149.8, 136.7, 120.7, 120.3, 104.9, 55.4, 54.9.

IR (Diamond-ATR, neat): $\tilde{\nu}$ / cm^{-1} = 2924, 1573, 1552, 1479, 1471, 1452, 1414, 1376, 1312, 1284, 1267, 1245, 1198, 1177, 1146, 1120, 1100, 1086, 1040, 1004, 989, 985, 956, 795, 755, 731, 719.

MS (EI, 70 eV): *m/z* (%) = 248 (52), 218 (100), 207 (27), 191 (17), 177 (37), 163 (11), 161 (15), 149 (14), 99 (14), 78 (46), 73 (11), 70 (10).

HRMS (EI-orbitrap): *m/z:* [M] calc. for [$C_{11}H_{11}N_3O_2S$]: 249.0572; found 249.0566.

m.p. (°C): 94.0 – 98.9.

Cyclohexyl(phenyl)(thiazol-2-yl)methanol (9dj)

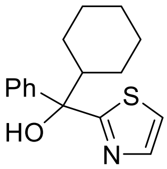

According to **TP2**, solutions of 2-bromothiazole (**7d**, 0.2 M, 1.0 equiv) in THF, 3-(chloromethyl)heptane (**2**, 0.2 M, 2.0 equiv) in *n*-hexane were prepared. The solution of **2** was pumped through the activated sodium packed-bed reactor (see **TP1**) by pump A (flow rate: 2.0 mL/min) into the precooling loop (V_{pre2} = 0.35 mL) cooled to –40 °C. The solution of **7d** was pumped by pump B (flow rate: 1.0 mL/min) through a precooling loop (V_{pre3} = 2.00 mL) cooled to –40 °C. The precooled solutions were mixed with an overall flow rate of 3.0 mL/min in a T-shaped mixer. The combined stream passed through a tube reactor and a metal needle (V_{R2} = 0.065 mL, t_{R2} = 1.3 s), subsequently upon reaching the steady state, it was injected into a flask charged with cyclohexyl(phenyl)methanone (**5j**, 56 mg, 0.3 mmol, 1.5 equiv) in THF (1.0 mL) at –20 °C for 60 s. The reaction mixture was stirred at –20 °C for 10 min and allowed to warm to 25 °C before sat. *aq.* NH_4Cl solution was added for quenching the reaction mixture. The aqueous layer was extracted three times with EtOAc (3×30 mL) and the combined organic layers were dried over anhydrous $MgSO_4$ and filtrated. After removal of the solvent, flash column chromatographical purification (silica gel, isohexane:EtOAc = 9:1) afforded the title compound **9dj** as a white solid (36 mg, 0.13 mmol, 66% yield).

^{1}H-NMR (400 MHz, $CDCl_3$): δ / ppm = 7.74 – 7.67 (m, 3H), 7.34 (dd, *J* = 8.5, 7.0 Hz, 2H), 7.26 – 7.20 (m, 2H), 3.66 (s, 1H), 2.49 (tt, *J* = 11.6, 3.2 Hz, 1H), 1.80 – 1.62 (m, 3H), 1.45 – 1.37 (m, 2H), 1.35 – 1.04 (m, 5H).

^{13}C-NMR (100 MHz, $CDCl_3$): δ / ppm = 178.0, 143.9, 141.8, 128.4 (2C), 127.2, 125.6 (2C), 119.4, 81.5, 48.7, 27.1, 26.7, 26.6, 26.5, 26.4.

IR (Diamond-ATR, neat): $\tilde{\nu}$ / cm^{-1} = 2928, 2852, 1501, 1493, 1446, 1431, 1178, 1167, 1153, 1100, 1081, 1071, 1057, 1034, 994, 966, 841, 770, 760, 717, 696, 623.

MS (EI, 70 eV): *m/z* (%) = 199 (100), 105 (18).

HRMS (EI-orbitrap): *m/z:* [M] calc. for [$C_{16}H_{19}NOS$]: 273.1187; found 273.1182.

m.p. (°C): 105.8 – 108.8.

(*E*)-2-(Thiazol-2-yl)-4-(2,6,6-trimethylcyclohex-2-en-1-yl)but-3-en-2-ol (9do)

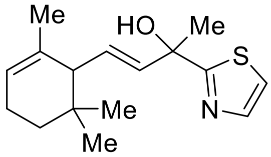

According to **TP2**, solutions of 2-bromothiazole (**7d**, 0.2 M, 1.0 equiv) in THF, 3-(chloromethyl)heptane (**2**, 0.2 M, 2.0 equiv) in *n*-hexane were prepared. The solution of **2** was pumped through the activated sodium packed-bed reactor (see **TP1**) by pump A (flow rate: 2.0 mL/min) into the precooling loop (V_{pre2} = 0.35 mL) cooled to –40 °C. The solution of **7d** was pumped by pump B (flow rate: 1.0 mL/min) through a precooling loop (V_{pre3} = 2.00 mL) cooled to –40 °C. The precooled solutions were mixed with an overall flow rate of 3.0 mL/min in a T-shaped mixer. The combined stream passed through a tube reactor and a metal needle (V_{R2} = 0.065 mL, t_{R2} = 1.3 s), subsequently upon reaching the steady state, it was injected into a flask charged with a racemic mixture of α-ionone (**5o**, 58 mg, 0.3 mmol, 1.5 equiv) in THF (1.0 mL) at –20 °C for 60 s. The reaction mixture was stirred at –20 °C for 10 min and allowed to warm to 25 °C before sat. *aq.* NH_4Cl solution was added for quenching the reaction mixture. The aqueous layer was extracted three times with EtOAc (3×30 mL) and the combined organic layers were dried over anhydrous $MgSO_4$ and filtrated. After removal of the solvent, flash column chromatographical purification (silica gel, isohexane:EtOAc = 9:1) afforded the title compound **7do** as a colorless oil (28 mg, 0.10 mmol, 50% yield, *dr* : 1:1).

Mixture of the two diastereoisomers:

^{1}H-NMR (400 MHz, CDCl$_3$): δ / ppm = 7.70 (d, *J* = 3.3 Hz, 1H), 7.27 (s, 1H), 5.86 (s, 1H), 5.82 (s, 1H), 5.63 (d, *J* = 9.4, 1H), 5.59 (d, *J* = 9.4, 1H), 5.42 – 5.37 (m, 1H), 3.17 (s, 1H), 2.14 (d, *J* = 9.4 Hz, 1H), 2.04 – 1.93 (m, 2H), 1.76 (s, 3H), 1.57 (q, *J* = 1.9 Hz, 3H), 1.53 (q, *J* = 2.0 Hz, 3H), 1.45 – 1.35 (m, 2H), 1.20 – 1.12 (m, 2H), 0.88 (s, 3H), 0.87 (s, 3H), 0.81 (s, 3H), 0.78 (s, 3H).

^{13}C-NMR (100 MHz, CDCl$_3$): δ / ppm = 177.9, 177.8, 142.3, 136.5, 136.4, 133.8, 133.8, 131.0, 131.0, 121.5, 121.4, 119.4, 74.9, 74.9, 53.8, 32.4, 32.3, 31.7, 31.6, 29.9, 29.8, 27.7, 27.6, 27.1, 27.1, 23.2, 23.1.

IR (Diamond-ATR, neat): $\tilde{\nu}$ / cm^{-1} = 3413, 3378, 3346, 3343, 3337, 3333, 2962, 2929, 2869, 1698, 1694, 1689, 1683, 1651, 1499, 1448, 1417, 1386, 1367, 1265, 1237, 1181, 1133, 1056, 1037, 977, 907, 732, 702.

MS (EI, 70 eV): *m/z* (%) = 203 (12), 202 (100), 188 (48), 187 (18), 186 (11), 178 (26), 176 (12), 174 (12), 173 (18), 162 (11), 150 (54), 141 (12), 138 (12), 137 (83), 136 (93), 129 (20), 128 (14), 121 (19), 115 (11), 112 (12), 111 (33), 93 (18), 91 (37), 86 (33), 79 (18), 77 (21).

HRMS (EI-orbitrap): *m/z:* [M – H_2O] calc. for [$C_{16}H_{21}NS$]: 259.1395; found 259.1390.

Benzo[*b*]thiophen-2-yl(4-chlorophenyl)(cyclopropyl)methanol (12am)

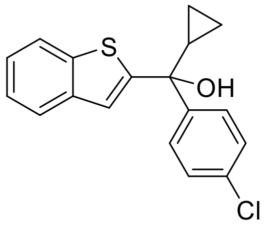

According to **TP3**, solutions of benzothiophene (**10a**, 0.2 M, 1.0 equiv) in THF and 3-(chloromethyl)heptane (**2**, 0.2 M, 2.0 equiv) in *n*-hexane were prepared. The solution of **2** was pumped through the activated sodium packed-bed reactor (see **TP1**) by pump A (flow rate: 2.0 mL/min) into the precooling loop (V_{pre2} = 0.35 mL) cooled to –40 °C. The solution of **10a** was pumped by pump B (flow rate: 1.0 mL/min) through a precooling loop (V_{pre3} = 2.00 mL) cooled to –40 °C. The precooled solutions were mixed with an overall flow rate of 3.0 mL/min in a T-shaped mixer. The combined stream passed through a tube reactor and a metal needle (V_{R2} = 0.065 mL, t_{R2} = 1.3 s). Subsequently upon reaching the steady state, it was injected into a flask charged with (4-chlorophenyl)(cyclopropyl)methanone (**5m**, 54 mg, 0.3 mmol, 1.5 equiv) in THF (1.0 mL) cooled to –20 °C for 60 s. The reaction mixture was stirred at –20 °C for 10 min and allowed to warm to 25 °C before sat. *aq.* NH_4Cl solution was added for quenching the reaction mixture. The aqueous layer was extracted three times with EtOAc (3×30 mL) and the combined organic layers were dried over anhydrous $MgSO_4$ and filtrated. After removal of the solvent, flash column chromatographical purification (silica gel, isohexane:EtOAc = 99:1 → 9:1) afforded the title compound **12am** as a slightly brownish oil (55 mg, 0.18 mmol, 87% yield).

^{1}H-NMR (400 MHz, $CDCl_3$): δ / ppm = 7.78 (d, *J* = 7.7 Hz, 1H), 7.72 (d, *J* = 7.3 Hz, 1H), 7.50 (d, *J* = 8.6 Hz, 2H), 7.37 – 7.27 (m, 4H), 7.24 (s, 1H), 2.20 (s, 1H), 1.73 (tt, *J* = 8.1, 5.5 Hz, 1H), 0.79 – 0.63 (m, 2H), 0.65 – 0.51 (m, 2H).

^{13}C-NMR (100 MHz, $CDCl_3$): δ / ppm = 152.8, 144.3, 139.9, 139.3, 133.5, 128.2 (2C), 127.9 (2C), 124.6, 124.5, 123.8, 122.5, 121.8, 75.6, 22.7, 2.7, 1.8.

IR (Diamond-ATR, neat): $\tilde{\nu}$ / cm^{-1} = 3570, 3560, 3556, 3515, 3463, 3448, 3419, 3057, 3007, 2923, 2919, 1708, 1703, 1592, 1573, 1487, 1469, 1457, 1434, 1397, 1376, 1365, 1329, 1305, 1249, 1168, 1150, 1128, 1101, 1091, 1066, 1052, 1025, 1013, 963, 947, 936, 926, 878, 859, 829, 791, 744, 726, 712.

MS (EI, 70 eV): *m/z* (%) = 314 (11), 288 (32), 287 (15), 286 (89), 275 (13), 273 (35), 245 (13), 210 (11), 161 (32), 160 (11), 147 (55), 141 (33), 139 (11), 139 (100), 134 (11), 89 (11).

HRMS (EI-orbitrap): *m/z:* [M] calc. for [$C_{18}H_{15}ClOS$]: 314.0532; found 314.0527.

Benzo[*b*]thiophen-2-yl(4-(trifluoromethyl)phenyl)methanone (12ac)

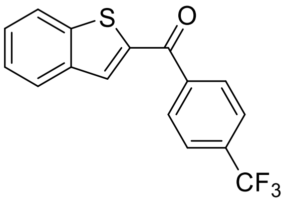

According to **TP3**, solutions of benzothiophene (**10a**, 0.2 M, 1.0 equiv) in THF and 3-(chloromethyl)heptane (**2**, 0.2 M, 2.0 equiv) in *n*-hexane were prepared. The solution of **2** was pumped through the activated sodium packed-bed reactor (see **TP1**) by pump A (flow rate: 2.0 mL/min) into the precooling loop (V_{pre2} = 0.35 mL) cooled to –40 °C. The solution of **10a** was pumped by pump B (flow rate: 1.0 mL/min) through a precooling loop (V_{pre3} = 2.00 mL) cooled to –40 °C. The precooled solutions were mixed with an overall flow rate of 3.0 mL/min in a T-shaped mixer. The combined stream passed through a tube reactor and a metal needle (V_{R2} = 0.065 mL, t_{R2} = 1.3 s). Subsequently upon reaching the steady state, it was injected into a flask charged with *N*-methoxy-*N*-methyl-4-(trifluoromethyl)benzamide (**5c**, 70 mg, 0.3 mmol, 1.5 equiv) in THF (1.0 mL) cooled to –20 °C for 60 s. The reaction mixture was stirred at –20 °C for 10 min and allowed to warm to 25 °C before sat. *aq.* NH_4Cl solution was added for quenching the reaction mixture. The aqueous layer was extracted three times with EtOAc (3×30 mL) and the combined organic layers were dried over anhydrous $MgSO_4$ and filtrated. After removal of the solvent, flash column chromatographical purification (silica gel, isohexane:EtOAc = 99.5:0.5 → 99:1) afforded the title compound **12ac** as colorless crystals (52 mg, 0.17 mmol, 85% yield).

^{1}H-NMR (400 MHz, $CDCl_3$): δ / ppm = 8.01 (d, *J* = 8.0 Hz, 2H), 7.91 (dd, *J* = 13.4, 8.1 Hz, 2H), 7.86 – 7.76 (m, 3H), 7.52 (ddd, *J* = 8.2, 7.1, 1.3 Hz, 1H), 7.44 (t, *J* = 7.0 Hz, 1H).

^{13}C-NMR (100 MHz, $CDCl_3$): δ / ppm = 188.7, 143.1, 142.5, 141.0, 139.1, 134.0 (q, *J* = 32.8 Hz), 133.0, 129.6 (2C), 128.0, 126.4, 125.7 (q, *J* = 3.8 Hz, 2C), 125.4, 123.8 (q, *J* = 272.7 Hz), 123.1.

IR (Diamond-ATR, neat): $\tilde{\nu}$ / cm^{-1} = 2923, 2919, 1629, 1614, 1592, 1577, 1574, 1555, 1510, 1503, 1494, 1458, 1454, 1428, 1405, 1323, 1310, 1289, 1246, 1190, 1181, 1158, 1134, 1130, 1107, 1062, 1015, 974, 944, 913, 882, 867, 855, 844, 837, 767, 745, 728, 725, 708, 700, 691, 678.

MS (EI, 70 eV): *m/z* (%) = 306 (53), 173 (12), 162 (10), 161 (100), 145 (21), 133 (14), 89 (16).

HRMS (EI-orbitrap): *m/z:* [M] calc. for [$C_{16}H_9F_3OS$]: 306.0326; found 306.0320.

m.p. (°C): 164.8 – 172.6.

Benzo[*b*]thiophen-2-yl(2,6-dichlorophenyl)methanol (12ag)

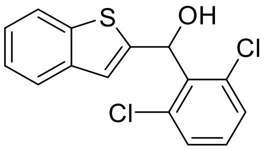

According to **TP3**, solutions of benzothiophene (**10a**, 0.2 M, 1.0 equiv) in THF and 3-(chloromethyl)heptane (**2**, 0.2 M, 2.0 equiv) in *n*-hexane were prepared. The solution of **2** was pumped through the activated sodium packed-bed reactor (see **TP1**) by pump A (flow rate: 2.0 mL/min) into the precooling loop (V_{pre2} = 0.35 mL) cooled to –40 °C. The solution of **10a** was pumped by pump B (flow rate: 1.0 mL/min) through a precooling loop (V_{pre3} = 2.00 mL) cooled to –40 °C. The precooled solutions were mixed with an overall flow rate of 3.0 mL/min in a T-shaped mixer. The combined stream passed through a tube reactor and a metal needle (V_{R2} = 0.065 mL, t_{R2} = 1.3 s). Subsequently upon reaching the steady state, it was injected into a flask charged with 2,6-dichlorobenzaldehyde (**5g**, 70 mg, 0.4 mmol, 2.0 equiv) in THF (1.0 mL) cooled to –20 °C for 60 s. The reaction mixture was stirred at –20 °C for 10 min and allowed to warm to 25 °C before sat. *aq.* NH_4Cl solution was added for quenching the reaction mixture. The aqueous layer was extracted three times with EtOAc (3×30 mL) and the combined organic layers were dried over anhydrous $MgSO_4$ and filtrated. After removal of the solvent, flash column chromatographical purification (silica gel, isohexane:EtOAc = 99:1 → 95:5) afforded the title compound **12ag** as a slightly brownish oil (45 mg, 0.15 mmol, 73% yield).

^{1}H-NMR (400 MHz, $CDCl_3$): δ / ppm = 7.83 (d, *J* = 6.9 Hz, 1H), 7.68 (dd, *J* = 6.8, 2.2 Hz, 1H), 7.41 (d, *J* = 8.0 Hz, 2H), 7.38 – 7.24 (m, 3H), 6.92 (s, 1H), 6.85 (dd, *J* = 11.3, 1.7 Hz, 1H), 3.92 (d, *J* = 11.3 Hz, 1H).

^{13}C-NMR (100 MHz, $CDCl_3$): δ / ppm = 146.6, 139.9, 139.8, 136.6, 135.1 (2C), 130.1, 129.6 (2C), 124.5, 124.3, 123.6, 122.5, 120.6, 70.6.

IR (Diamond-ATR, neat): $\tilde{\nu}$ / cm^{-1} = 3549, 3415, 3358, 3056, 2923, 1579, 1561, 1457, 1434, 1397, 1328, 1302, 1249, 1237, 1201, 1178, 1149, 1132, 1107, 1087, 1072, 1019, 1008, 972, 936, 906, 858, 840, 820, 776, 755, 743, 725, 704, 678.

MS (EI, 70 eV): *m/z* (%) = 310 (12), 308 (18), 255 (11), 221 (10), 175 (15), 173 (23), 135 (100), 134 (36), 91 (12).

HRMS (EI-orbitrap): *m/z:* [M] calc. for [$C_{15}H_{10}Cl_2OS$]: 307.9829; found 307.9823.

2-((1-Butyl-1*H*-imidazol-2-yl)thio)pyridine (12bl)

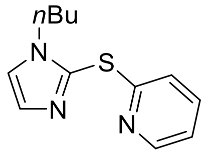

According to **TP3**, solutions of 1-butyl-1*H*-imidazole (**10b**, 0.2 M, 1.0 equiv) in THF and 3-(chloromethyl)heptane (**2**, 0.2 M, 2.0 equiv) in *n*-hexane were prepared. The solution of **2** was pumped through the activated sodium packed-bed reactor (see **TP1**) by pump A (flow rate: 2.0 mL/min) into the precooling loop (V_{pre2} = 0.35 mL) cooled to –40 °C. The solution of **10b** was pumped by pump B (flow rate: 1.0 mL/min) through a precooling loop (V_{pre3} = 2.00 mL) cooled to –40 °C. The precooled solutions were mixed with an overall flow rate of 3.0 mL/min in a T-shaped mixer. The combined stream passed through a tube reactor and a metal needle (V_{R2} = 0.065 mL, t_{R2} = 1.3 s). Subsequently upon reaching the steady state, it was injected into a flask charged with aldrithiol (**5l**, 66 mg, 0.3 mmol, 1.5 equiv) in THF (1.0 mL) cooled to –20 °C for 60 s. The reaction mixture was stirred at –20 °C for 10 min and allowed to warm to 25 °C before sat. *aq.* NH_4Cl solution was added for quenching the reaction mixture. The aqueous layer was extracted three times with EtOAc (3×30 mL) and the combined organic layers were dried over anhydrous $MgSO_4$ and filtrated. After removal of the solvent, flash column chromatographical purification (silica gel, isohexane:EtOAc = 50:50 → 20:80) afforded the title compound **12bl** as a yellow oil (37 mg, 0.16 mmol, 79% yield).

^{1}H-NMR (400 MHz, $CDCl_3$): δ / ppm = 8.39 (m, 1H), 7.48 (m, 1.9 Hz, 1H), 7.28 (d, *J* = 1.3 Hz, 1H), 7.17 (d, *J* = 1.3 Hz, 1H), 7.02 (m, 1H), 6.83 (dt, *J* = 8.0, 1.1 Hz, 1H), 4.04 (t, *J* = 7.3 Hz, 2H), 1.72 – 1.60 (m, 2H), 1.29 – 1.23 (m, 2H), 0.86 (t, *J* = 7.4 Hz, 3H).

^{13}C-NMR (100 MHz, $CDCl_3$): δ / ppm = 159.5, 149.8, 137.2, 135.6, 131.0, 123.0, 121.2, 120.6, 47.2, 33.1, 19.8, 13.7.

IR (Diamond-ATR, neat): $\tilde{\nu}$ / cm^{-1} = 3103, 3043, 2956, 2930, 2871, 1572, 1560, 1501, 1494, 1447, 1428, 1416, 1392, 1378, 1341, 1273, 1148, 1116, 1085, 1065, 1044, 985, 968, 914, 756, 720, 691.

MS (EI, 70 eV): *m/z* (%) = 233 (10), 205 (10), 204 (100), 200 (10), 191 (24), 190 (12), 176 (46), 158 (23), 155 (64), 134 (17), 133 (11), 123 (47), 122 (22), 119 (46), 118 (11), 113 (10), 111 (34), 96 (11), 84 (11), 81 (17), 78 (80).

HRMS (EI-orbitrap): *m/z:* [M] calc. for [$C_{12}H_{15}N_3S$]: 233.0987; found: 233.0988.

***N*-((1-Butyl-1*H*-imidazol-2-yl)(phenyl)methyl)aniline (12bd)**

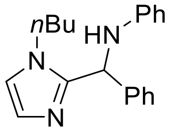

According to **TP3**, solutions of 1-butyl-1*H*-imidazole (**10b**, 0.2 M, 1.0 equiv) in THF and 3-(chloromethyl)heptane (**2**, 0.2 M, 2.0 equiv) in *n*-hexane were prepared. The solution of **2** was pumped through the activated sodium packed-bed reactor (see **TP1**) by pump A (flow rate: 2.0 mL/min) into the precooling loop (V_{pre2} = 0.35 mL) cooled to –40 °C. The solution of **10b** was pumped by pump B (flow rate: 1.0 mL/min) through a precooling loop (V_{pre3} = 2.00 mL) cooled to –40 °C. The precooled solutions were mixed with an overall flow rate of 3.0 mL/min in a T-shaped mixer. The combined stream passed through a tube reactor and a metal needle (V_{R2} = 0.065 mL, t_{R2} = 1.3 s). Subsequently upon reaching the steady state, it was injected into a flask charged with *N*,1-diphenylmethanimine (**5d**, 54 mg, 0.3 mmol, 1.5 equiv) in THF (1.0 mL) cooled to –20 °C for 60 s. The reaction mixture was stirred at –20 °C for 10 min and allowed to warm to 25 °C before sat. *aq.* NH_4Cl solution was added for quenching the reaction mixture. The aqueous layer was extracted three times with EtOAc (3×30 mL) and the combined organic layers were dried over anhydrous $MgSO_4$ and filtrated. After removal of the solvent, flash column chromatographical purification (silica gel, isohexane:EtOAc = 8:2 → 7:3) afforded the title compound **12bd** as a colorless oil (42 mg, 0.14 mmol, 69% yield).

^{1}H-NMR (400 MHz, $CDCl_3$): δ / ppm = 7.45 (d, *J* = 7.5 Hz, 2H), 7.36 (t, *J* = 7.6 Hz, 2H), 7.29 (d, *J* = 7.3 Hz, 1H), 7.21 – 7.12 (m, 2H), 7.07 (d, *J* = 1.3 Hz, 1H), 6.88 (s, 1H), 6.78 – 6.64 (m, 3H), 5.65 (s, 1H), 5.32 (s, 1H), 4.02 – 3.76 (m, 2H), 1.70 – 1.44 (m, 2H), 1.35 – 1.23 (m, 2H), 0.90 (t, *J* = 7.3 Hz, 3H).

^{13}C-NMR (100 MHz, $CDCl_3$): δ / ppm = 147.5, 146.7, 140.5, 129.2 (2C), 128.9 (2C), 127.8, 127.7, 127.6 (2C), 119.9, 117.9, 113.6 (2C), 55.2, 45.8, 32.8, 19.9, 13.7.

IR (Diamond-ATR, neat): $\tilde{\nu}$ / cm^{-1} = 3388, 3352, 3105, 3025, 2957, 2930, 2871, 1600, 1573, 1567, 1535, 1503, 1486, 1460, 1454, 1422, 1378, 1371, 1315, 1272, 1180, 1154, 1137, 1114, 1095, 1077, 1062, 1046, 1028, 992, 935, 869, 841, 827, 744, 690.

MS (EI, 70 eV): *m/z* (%) = 214 (15), 213 (100), 207 (34), 180 (17), 169 (11), 157 (85), 156 (18), 130 (17), 77 (21).

HRMS (EI-orbitrap): *m/z:* [M] calc. for [$C_{20}H_{23}N_3$]: 305.1892; found: 305.1886.

2-(1-butyl-1H-imidazol-2-yl)adamantan-2-ol (12bf)

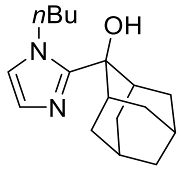

According to **TP3**, solutions of 1-butyl-1*H*-imidazole (**10b**, 0.2 M, 1.0 equiv) in THF and 3-(chloromethyl)heptane (**2**, 0.2 M, 2.0 equiv) in *n*-hexane were prepared. The solution of **2** was pumped through the activated sodium packed-bed reactor (see **TP1**) by pump A (flow rate: 2.0 mL/min) into the precooling loop (V_{pre2} = 0.35 mL) cooled to –40 °C. The solution of **10b** was pumped by pump B (flow rate: 1.0 mL/min) through a precooling loop (V_{pre3} = 2.00 mL) cooled to –40 °C. The precooled solutions were mixed with an overall flow rate of 3.0 mL/min in a T-shaped mixer. The combined stream passed through a tube reactor and a metal needle (V_{R2} = 0.065 mL, t_{R2} = 1.3 s). Subsequently upon reaching the steady state, it was injected into a flask charged with adamantanone (**5f**, 45 mg, 0.3 mmol, 1.5 equiv) in THF (1.0 mL) cooled to –20 °C for 60 s. The reaction mixture was stirred at –20 °C for 10 min and allowed to warm to 25 °C before sat. *aq.* NH_4Cl solution was added for quenching the reaction mixture. The aqueous layer was extracted three times with EtOAc (3×30 mL) and the combined organic layers were dried over anhydrous $MgSO_4$ and filtrated. After removal of the solvent, flash column chromatographical purification (silica gel, isohexane:EtOAc = 8:2 → 7:3) afforded the title compound **12bf** as a yellow oil (30 mg, 0.11 mmol, 55% yield).

^{1}H-NMR (400 MHz, $CDCl_3$): δ / ppm = 6.86 (m, 2H), 4.21 – 4.06 (m, 2H), 2.46 – 2.28 (m, 5H), 2.13 – 2.04 (m, 2H), 1.84 (s, 1H), 1.79 – 1.60 (m, 9H), 1.43 – 1.31 (m, 2H), 0.95 (t, *J* = 7.4 Hz, 3H).

^{13}C-NMR (100 MHz, $CDCl_3$): δ / ppm = 150.3, 126.1, 120.9, 75.3, 47.2, 37.9, 36.9 (2C), 35.1 (2C), 33.6, 32.9 (2C), 27.2, 27.0, 20.2, 13.9.

IR (Diamond-ATR, neat): $\tilde{\nu}$ / cm^{-1} = 3333, 3322, 2955, 2934, 2910, 2896, 2885, 2864, 2848, 1483, 1478, 1467, 1453, 1412, 1390, 1377, 1368, 1357, 1349, 1336, 1296, 1291, 1270, 1260, 1188, 1182, 1109, 1103, 1078, 1048, 1042, 1012, 996, 970, 939, 926, 907, 853, 753, 748, 731, 654.

MS (EI, 70 eV): *m/z* (%) = 275 (19), 274 (85), 273 (15), 257 (45), 246 (18), 245 (34), 232 (25), 231 (20), 227 (30), 217 (20), 203 (13), 191 (21), 179 (18), 151 (39), 151 (14), 123 (100), 123 (15), 96 (20), 95 (25), 91 (18), 82 (37), 79 (25), 69 (51), 68 (14), 55 (12), 40 (30).

HRMS (EI-orbitrap): *m/z:* [M] calc. for [$C_{17}H_{26}N_2O$]: 274.2045; found: 274.2040.

(4-Chlorophenyl)(cyclopropyl)(2,6-dimethoxyphenyl)methanol (12cm)

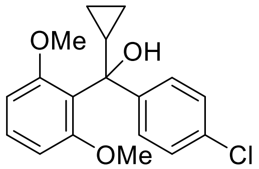

According to **TP3**, solutions of 1,3-dimethoxybenzene (**10c**, 0.2 M, 1.0 equiv) in THF and 3-(chloromethyl)heptane (**2**, 0.2 M, 2.0 equiv) in *n*-hexane were prepared. The solution of **2** was pumped through the activated sodium packed-bed reactor (see **TP1**) by pump A (flow rate: 2.0 mL/min) into the precooling loop (V_{pre2} = 0.35 mL) cooled to –40 °C. The solution of **10c** was pumped by pump B (flow rate: 1.0 mL/min) through a precooling loop (V_{pre3} = 2.00 mL) cooled to –40 °C. The precooled solutions were mixed with an overall flow rate of 3.0 mL/min in a T-shaped mixer. The combined stream passed through a tube reactor and a metal needle (V_{R2} = 0.065 mL, t_{R2} = 1.3 s). Subsequently upon reaching the steady state, it was injected into a flask charged with (4-chlorophenyl)(cyclopropyl)methanone (**5m**, 54 mg, 0.3 mmol, 1.5 equiv) in THF (1.0 mL) cooled to –20 °C for 60 s. The reaction mixture was stirred at –20 °C for 10 min and allowed to warm to 25 °C before sat. *aq.* NH_4Cl solution was added for quenching the reaction mixture. The aqueous layer was extracted three times with EtOAc (3×30 mL) and the combined organic layers were dried over anhydrous $MgSO_4$ and filtrated. After removal of the solvent, flash column chromatographical purification (silica gel, isohexane:EtOAc = 99:1 → 95:5) afforded the title compound **12cm** as colorless crystals (55 mg, 0.17 mmol, 86% yield).

^{1}H-NMR (400 MHz, $CDCl_3$): δ / ppm = 7.43 (d, *J* = 8.5 Hz, 2H), 7.25 – 7.17 (m, 3H), 6.61 (d, *J* = 8.3 Hz, 2H), 6.05 (s, 1H), 3.61 (s, 6H), 1.91 (tt, *J* = 8.3, 6.0 Hz, 1H), 0.80 – 0.70 (m, 1H), 0.61 – 0.49 (m, 2H), 0.48 – 0.36 (m, 1H).

^{13}C-NMR (100 MHz, $CDCl_3$): δ / ppm = 158.2 (2C), 148.7, 131.6, 128.5, 127.7 (2C), 127.5 (2C), 123.9, 106.7 (2C), 77.8, 56.4 (2C), 20.2, 2.2, 1.2.

IR (Diamond-ATR, neat): $\tilde{\nu}$ / cm^{-1} = 3460, 3007, 2982, 2950, 2918, 2870, 2847, 1592, 1584, 1567, 1490, 1470, 1454, 1442, 1432, 1412, 1397, 1376, 1356, 1278, 1245, 1204, 1189, 1171, 1139, 1098, 1093, 1085, 1048, 1020, 1012, 982, 962, 951, 897, 866, 843, 828, 775, 742, 731, 722, 704.

MS (EI, 70 eV): *m/z* (%) = 290 (25), 166 (10), 165 (100), 139 (18), 137 (11).

HRMS (EI-orbitrap): *m/z:* [M – C_2H_4] calc. for [$C_{16}H_{15}ClO_3$]: 290.0710; found: 290.0705.

m.p. (°C): 125.6 – 130.3.

Butyl(2,6-dimethoxyphenyl)sulfane (12cp)

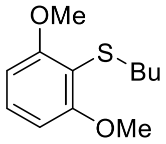

According to **TP3**, solutions of 1,3-dimethoxybenzene (**10c**, 0.2 M, 1.0 equiv) in THF and 3-(chloromethyl)heptane (**2**, 0.2 M, 2.0 equiv) in *n*-hexane were prepared. The solution of **2** was pumped through the activated sodium packed-bed reactor (see **TP1**) by pump A (flow rate: 2.0 mL/min) into the precooling loop (V_{pre2} = 0.35 mL) cooled to –40 °C. The solution of **10c** was pumped by pump B (flow rate: 1.0 mL/min) through a precooling loop (V_{pre3} = 2.00 mL) cooled to –40 °C. The precooled solutions were mixed with an overall flow rate of 3.0 mL/min in a T-shaped mixer. The combined stream passed through a tube reactor and a metal needle (V_{R2} = 0.065 mL, t_{R2} = 1.3 s). Subsequently upon reaching the steady state, it was injected into a flask charged with 1,2-dibutyldisulfane (**5p**, 54 mg, 0.3 mmol, 1.5 equiv) in THF (1.0 mL) cooled to –20 °C for 60 s. The reaction mixture was stirred at –20 °C for 10 min and allowed to warm to 25 °C before sat. *aq.* NH_4Cl solution was added for quenching the reaction mixture. The aqueous layer was extracted three times with EtOAc (3×30 mL) and the combined organic layers were dried over anhydrous $MgSO_4$ and filtrated. After removal of the solvent, flash column chromatographical purification (silica gel, isohexane:EtOAc = 99:1 → 95:5) afforded the title compound **12cp** as a slightly yellow oil (40 mg, 0.18 mmol, 88% yield).

^{1}H-NMR (400 MHz, $CDCl_3$): δ / ppm = 7.23 (t, *J* = 8.4 Hz, 1H), 6.56 (d, *J* = 8.4 Hz, 2H), 3.88 (s, 6H), 2.80 (dd, *J* = 7.9, 6.7 Hz, 2H), 1.51 – 1.43 (m, 2H), 1.43 – 1.32 (m, 2H), 0.86 (t, *J* = 7.2 Hz, 3H).

^{13}C-NMR (100 MHz, $CDCl_3$): δ / ppm = 161.2 (2C), 129.4, 110.6, 104.1 (2C), 56.3 (2C), 33.8, 31.8, 22.0, 13.8.

IR (Diamond-ATR, neat): $\tilde{\nu}$ / cm^{-1} = 2955, 2870, 2835, 1578, 1556, 1467, 1429, 1377, 1291, 1267, 1245, 1186, 1171, 1102, 1059, 1032, 915, 770, 755, 747, 715.

MS (EI, 70 eV): *m/z* (%) = 226 (37), 183 (10), 170 (100), 168 (30), 167 (13), 155 (21), 127 (16), 124 (11).

HRMS (EI-orbitrap): *m/z:* [M] calc. for [$C_{12}H_{18}O_2S$]: 226.1028; found 226.1020.

1,3-Dimethoxy-2-octylbenzene (12cq)

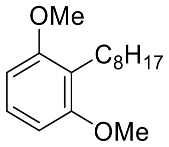

According to **TP3**, solutions of 1,3-dimethoxybenzene (**10c**, 0.2 M, 1.0 equiv) in THF and 3-(chloromethyl)heptane (**2**, 0.2 M, 2.0 equiv) in *n*-hexane were prepared. The solution of **2** was pumped through the activated sodium packed-bed reactor (see **TP1**) by pump A (flow rate: 2.0 mL/min) into the precooling loop (V_{pre2} = 0.35 mL) cooled to –40 °C. The solution of **10c** was pumped by pump B (flow rate: 1.0 mL/min) through a precooling loop (V_{pre3} = 2.00 mL) cooled to –40 °C. The precooled solutions were mixed with an overall flow rate of 3.0 mL/min in a T-shaped mixer. The combined stream passed through a tube reactor and a metal needle (V_{R2} = 0.065 mL, t_{R2} = 1.3 s). Subsequently upon reaching the steady state, it was injected into a flask charged with 1-iodooctane (**5q**, 72 mg, 0.3 mmol, 1.5 equiv) in THF (1.0 mL) cooled to –20 °C for 60 s. The reaction mixture was stirred at –20 °C for 10 min and allowed to warm to 25 °C before sat. *aq.* NH_4Cl solution was added for quenching the reaction mixture. The aqueous layer was extracted three times with EtOAc (3×30 mL) and the combined organic layers were dried over anhydrous $MgSO_4$ and filtrated. After removal of the solvent, flash column chromatographical purification (silica gel, isohexane:EtOAc = 100:0 → 95:5) afforded the title compound **12cq** as a colorless oil (23 mg, 0.09 mmol, 46% yield).

^{1}H-NMR (400 MHz, $CDCl_3$): δ / ppm = 7.11 (t, *J* = 8.3 Hz, 1H), 6.54 (d, *J* = 8.3 Hz, 2H), 3.81 (s, 6H), 2.67 – 2.57 (m, 2H), 1.51 – 1.42 (m, 2H), 1.35 – 1.24 (m, 10H), 0.91 – 0.85 (m, 3H).

^{13}C-NMR (100 MHz, $CDCl_3$): δ / ppm = 158.4 (2C), 126.5, 119.8, 103.8 (2C), 55.8 (2C), 32.1, 30.0, 29.7, 29.5, 29.4, 23.0, 22.9, 14.3.

IR (Diamond-ATR, neat): $\tilde{\nu}$ / cm^{-1} = 2953, 2923, 2853, 2834, 1593, 1573, 1566, 1562, 1472, 1455, 1434, 1377, 1328, 1275, 1254, 1187, 1171, 1156, 1127, 1090, 1043, 773, 722, 697, 686.

MS (EI, 70 eV): *m/z* (%) = 250 (12), 151 (100), 123 (13), 91 (13).

HRMS (EI-orbitrap): *m/z:* [M] calc. for [$C_{16}H_{26}O_2$]: 250.1933; found 250.1926.

NMR data

Naphthalen-1-yldiphenylmethanol (6aa)

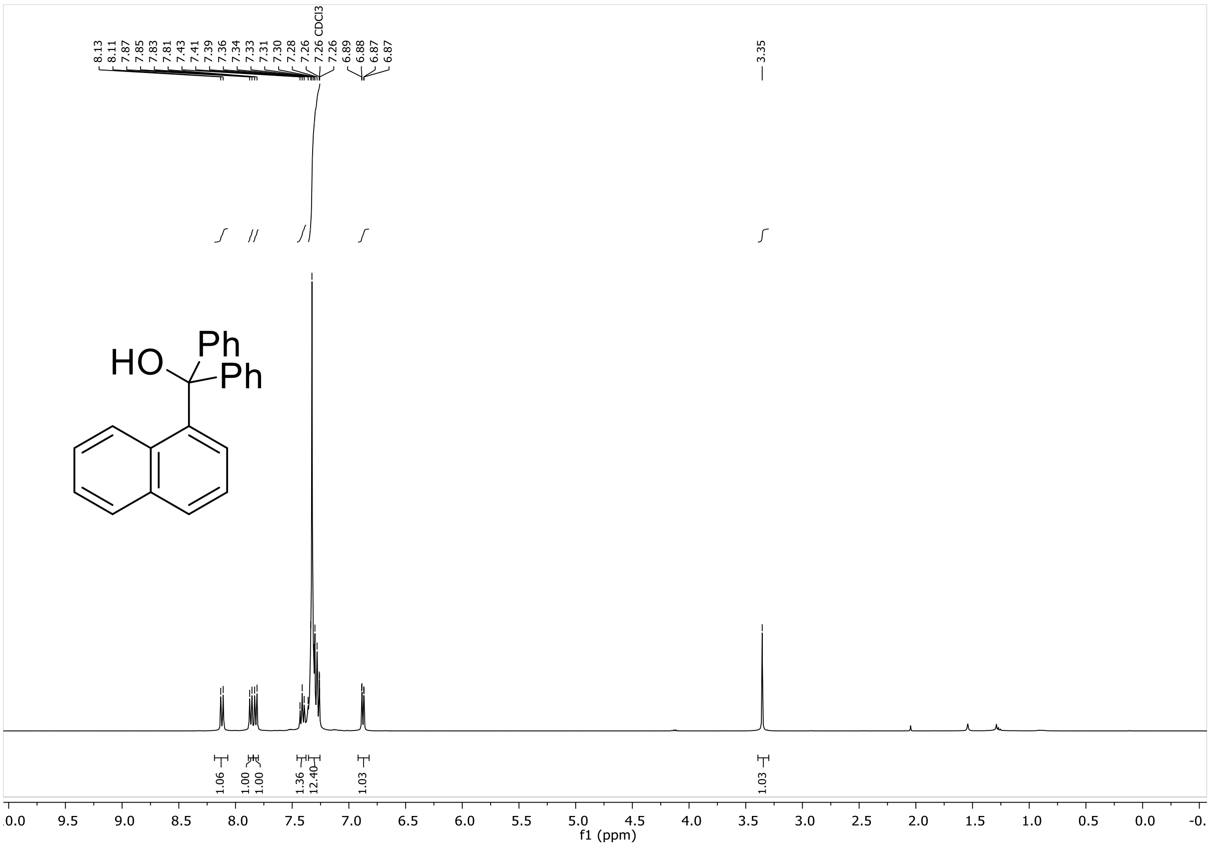

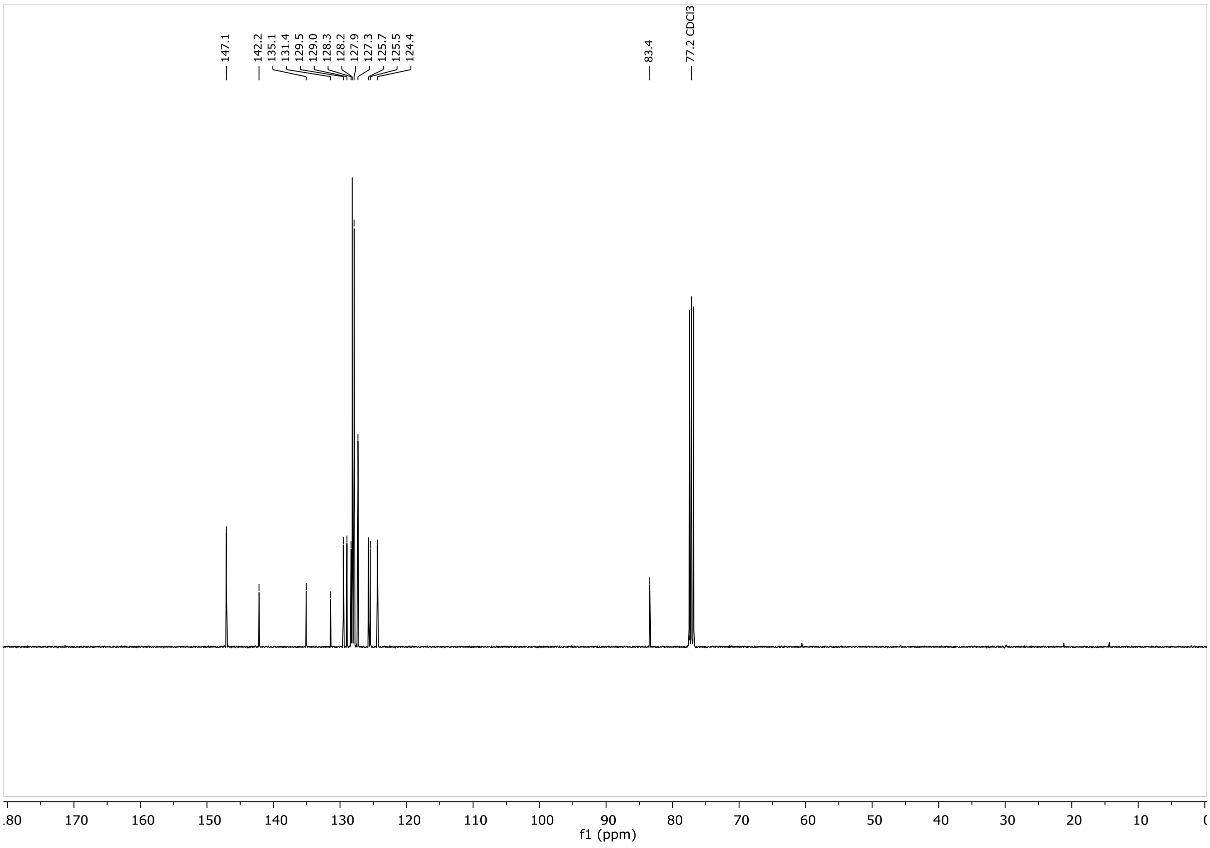

2-Ethyl-1-(naphthalen-1-yl)butan-1-ol (6ab)

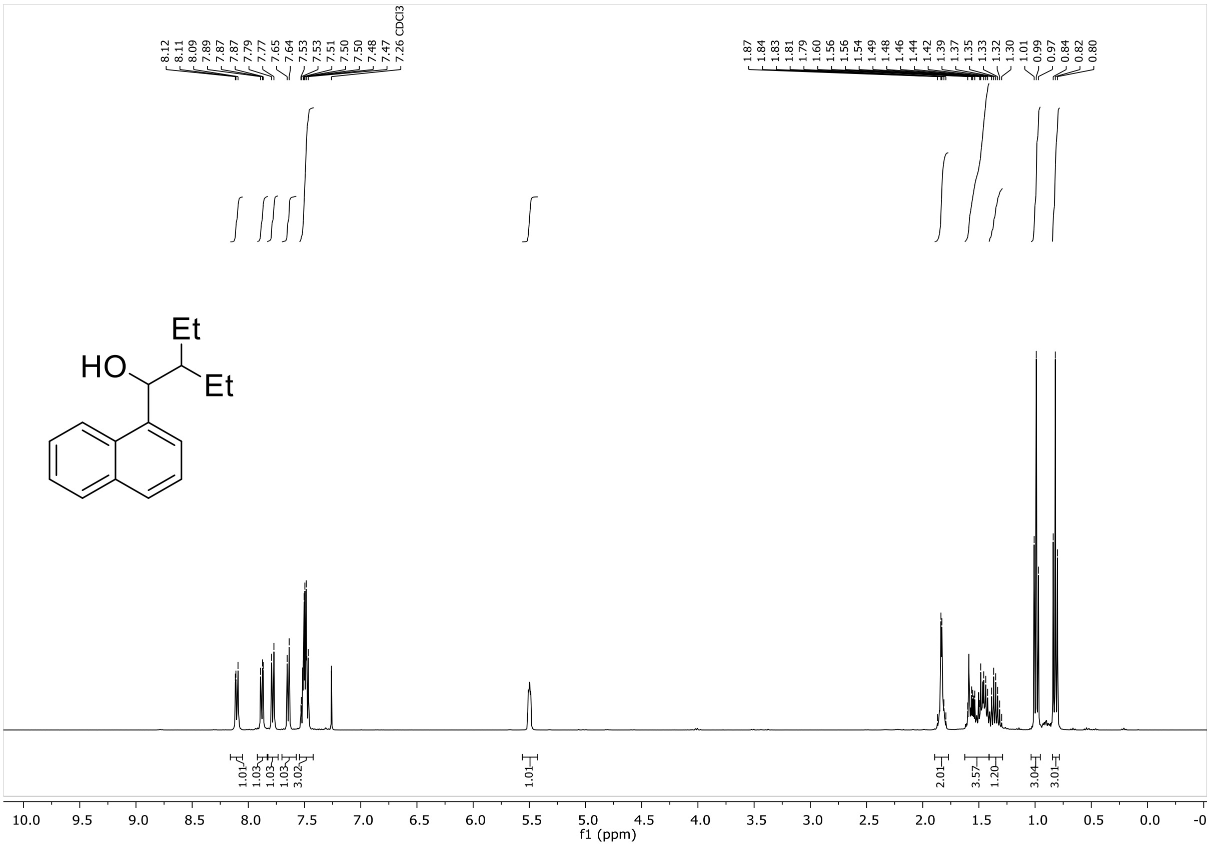

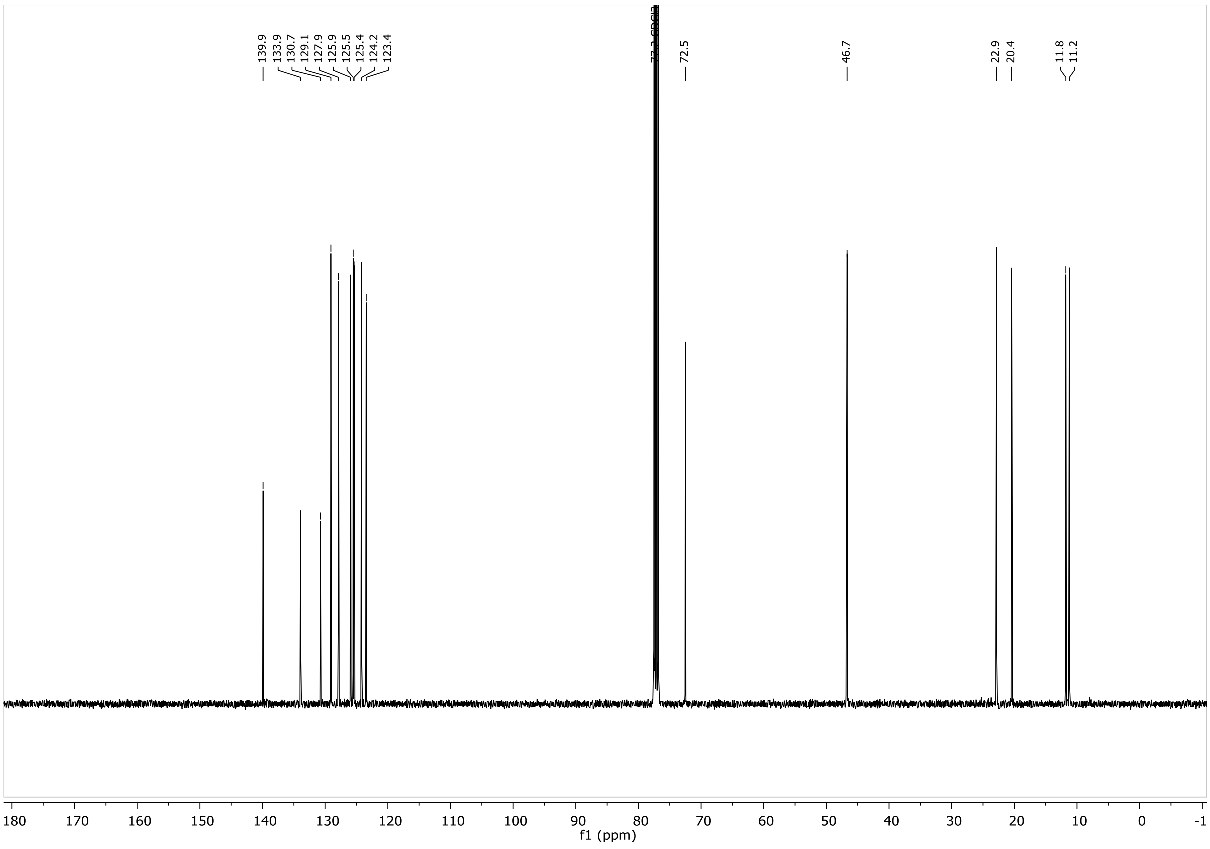

(3,5-Di-tert-butylphenyl)(4-(trifluoromethyl)phenyl)methanone (6bc)

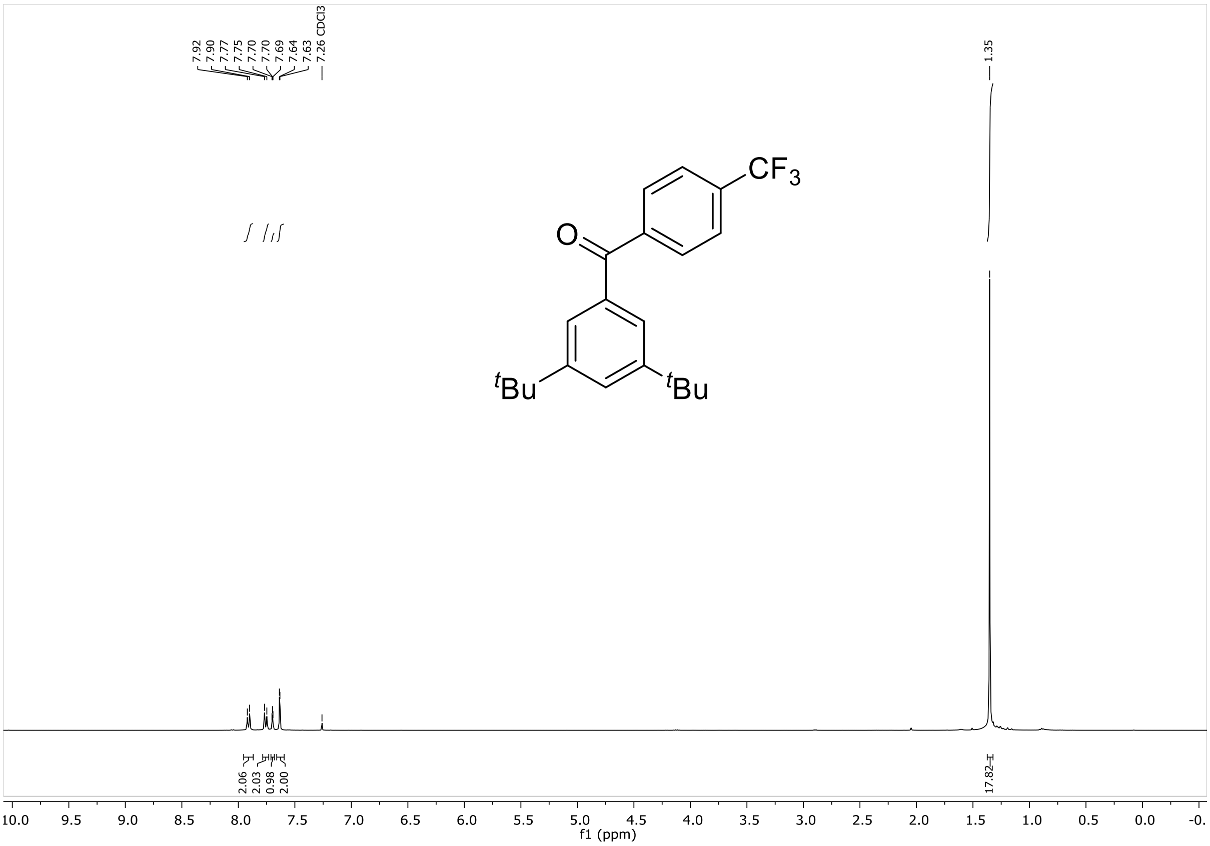

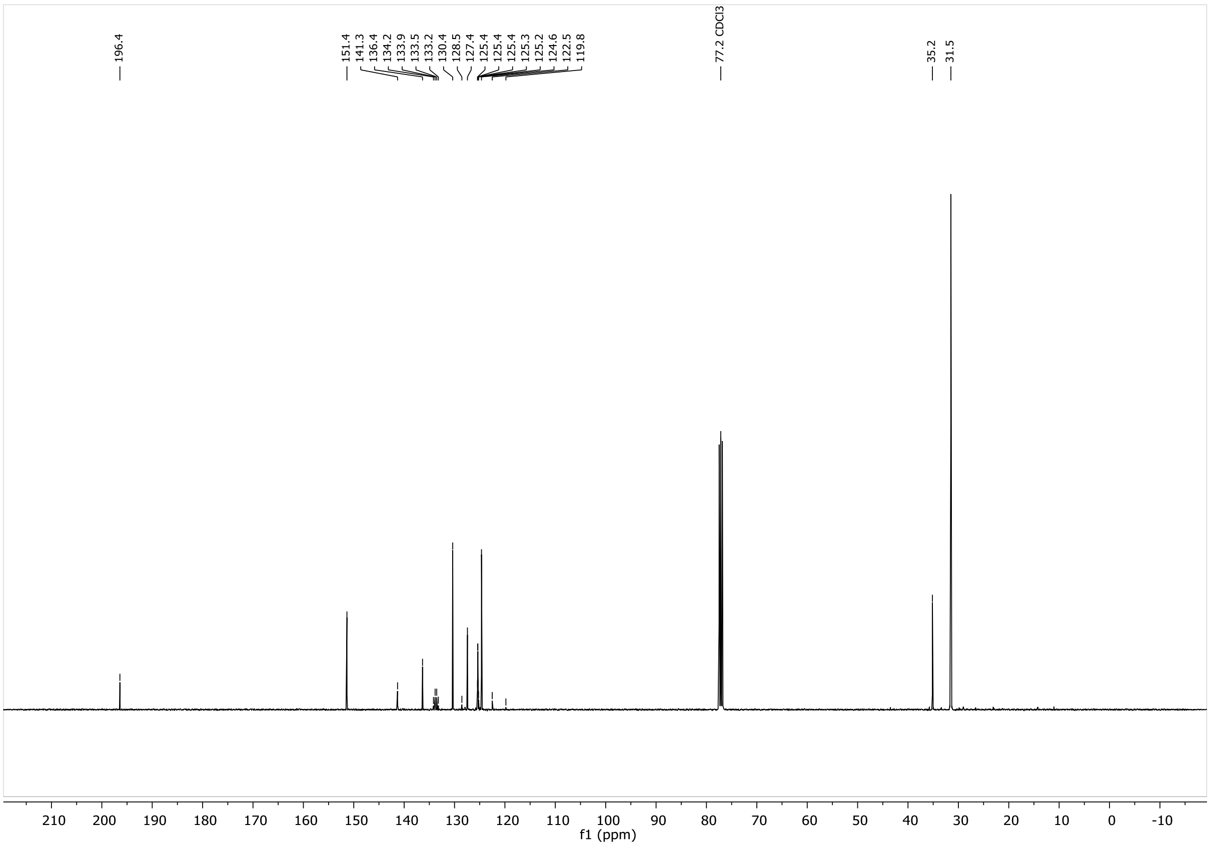

N-((3,5-Di-tert-butylphenyl)(phenyl)methyl)aniline (6bd)

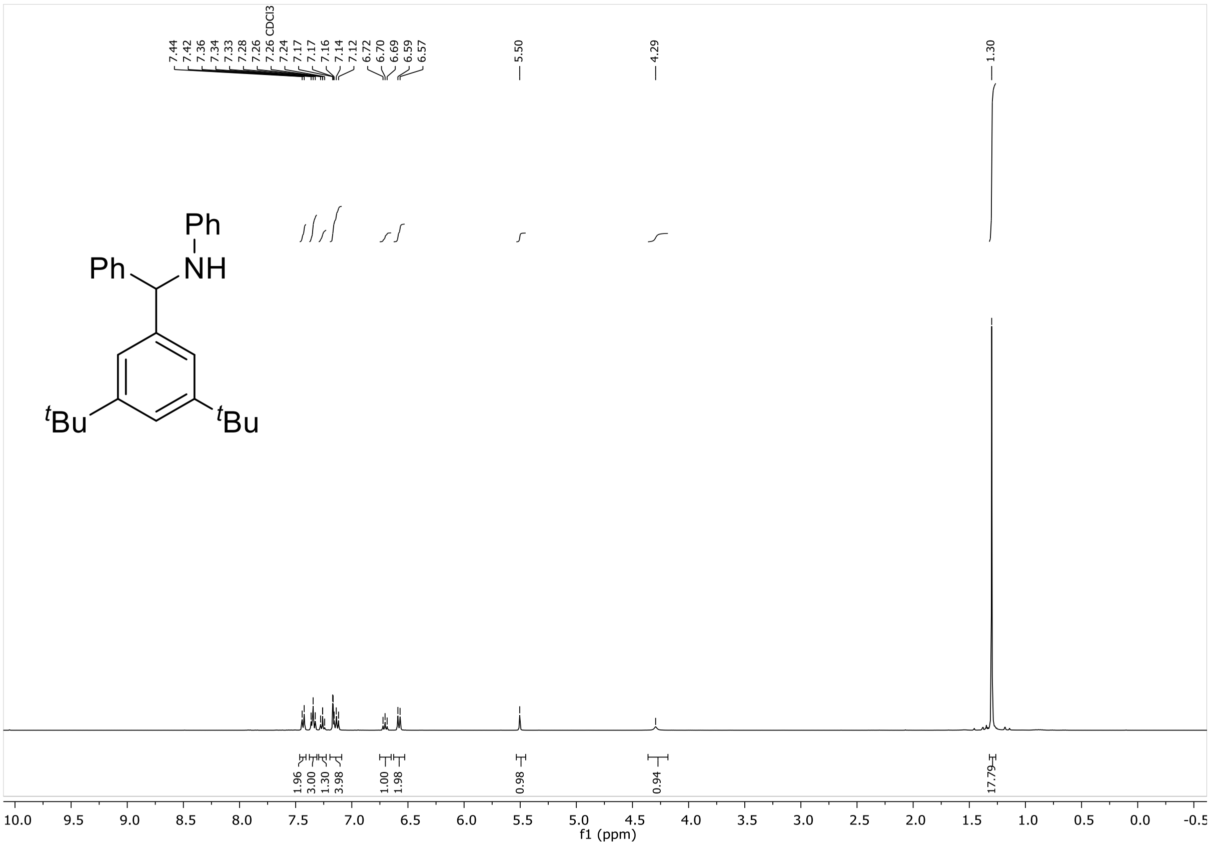

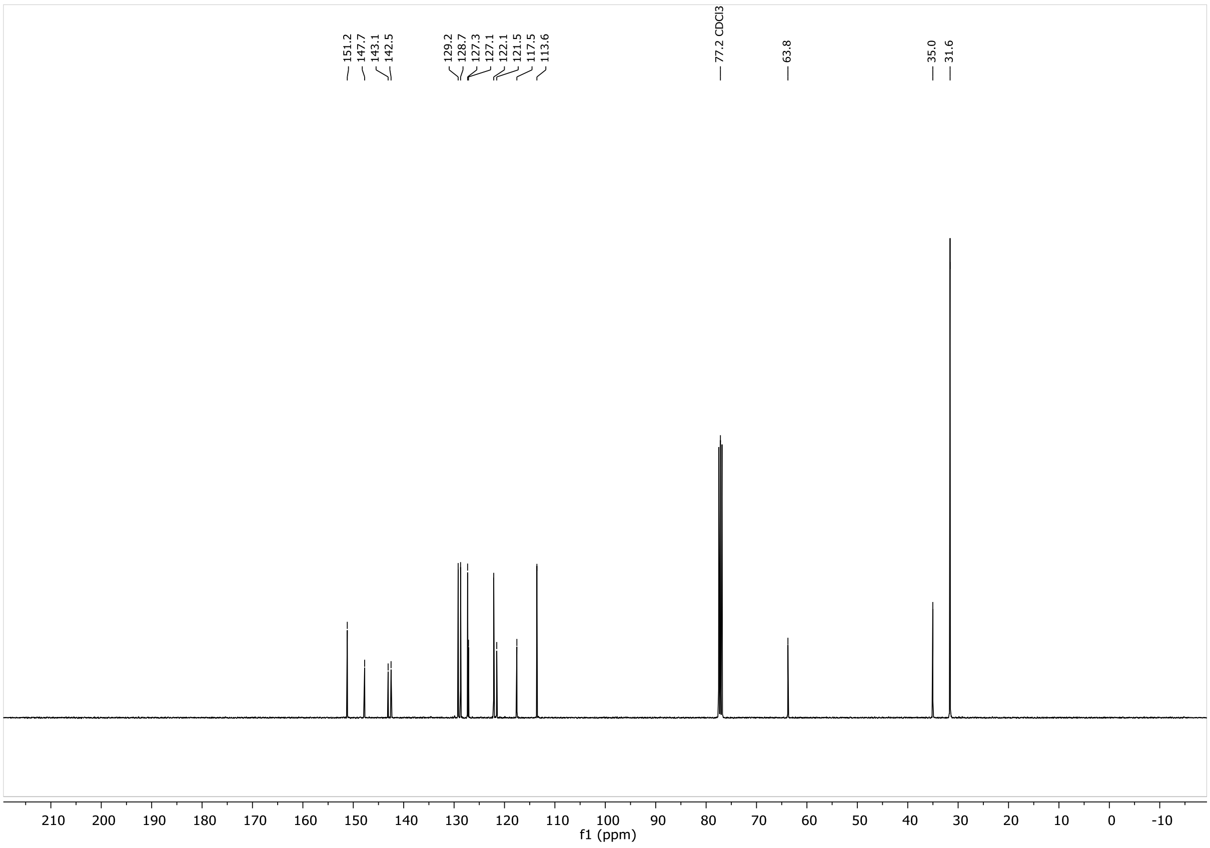

***N*-((4-Chlorophenyl)(phenyl)methyl)aniline (6cd)**

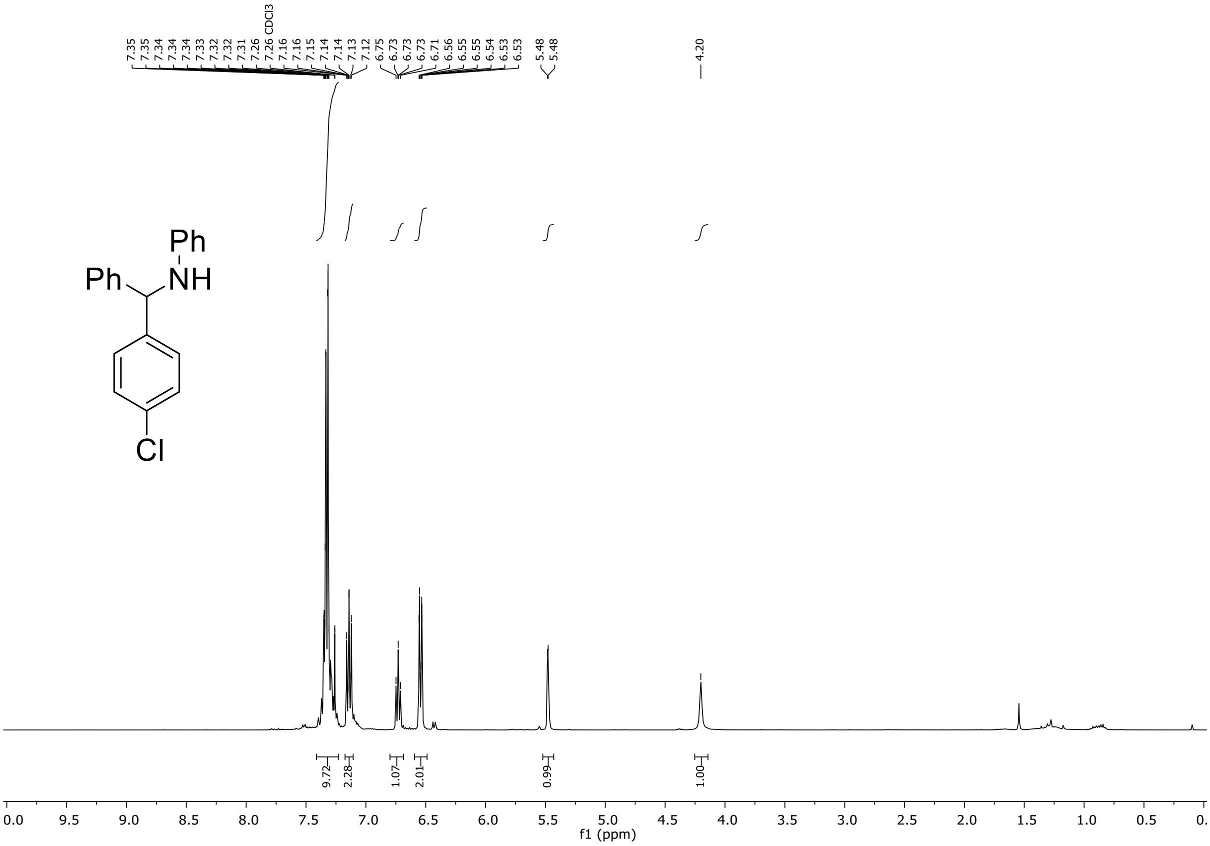

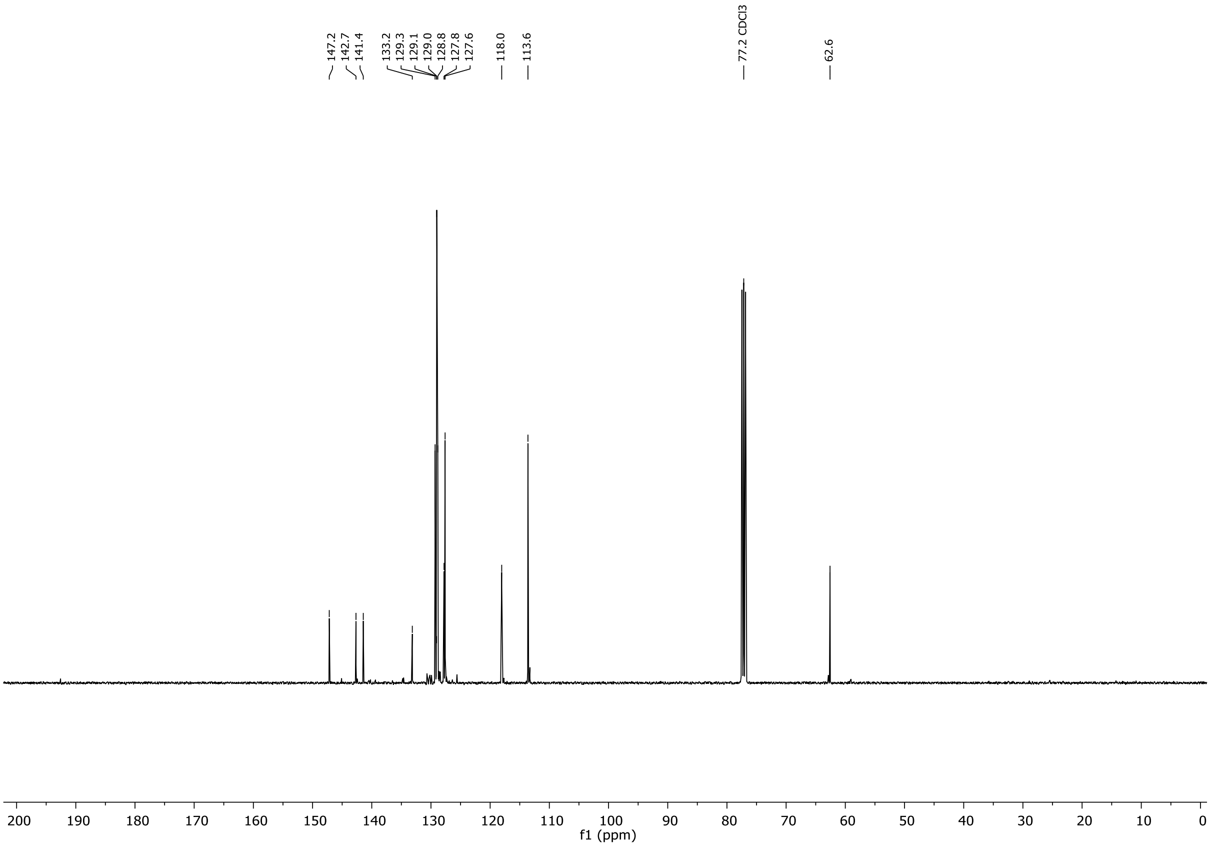

(4-Chlorophenyl)dicyclopropylmethanol (6ce)

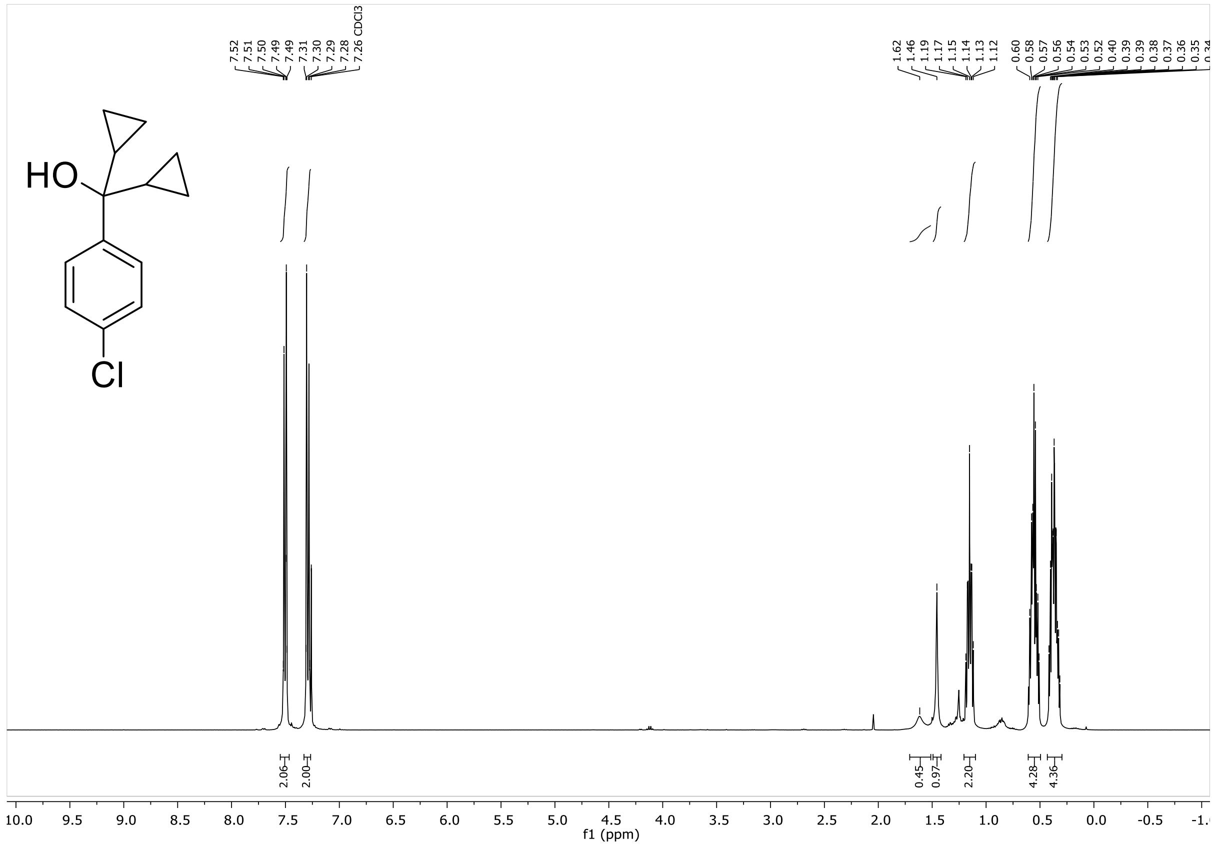

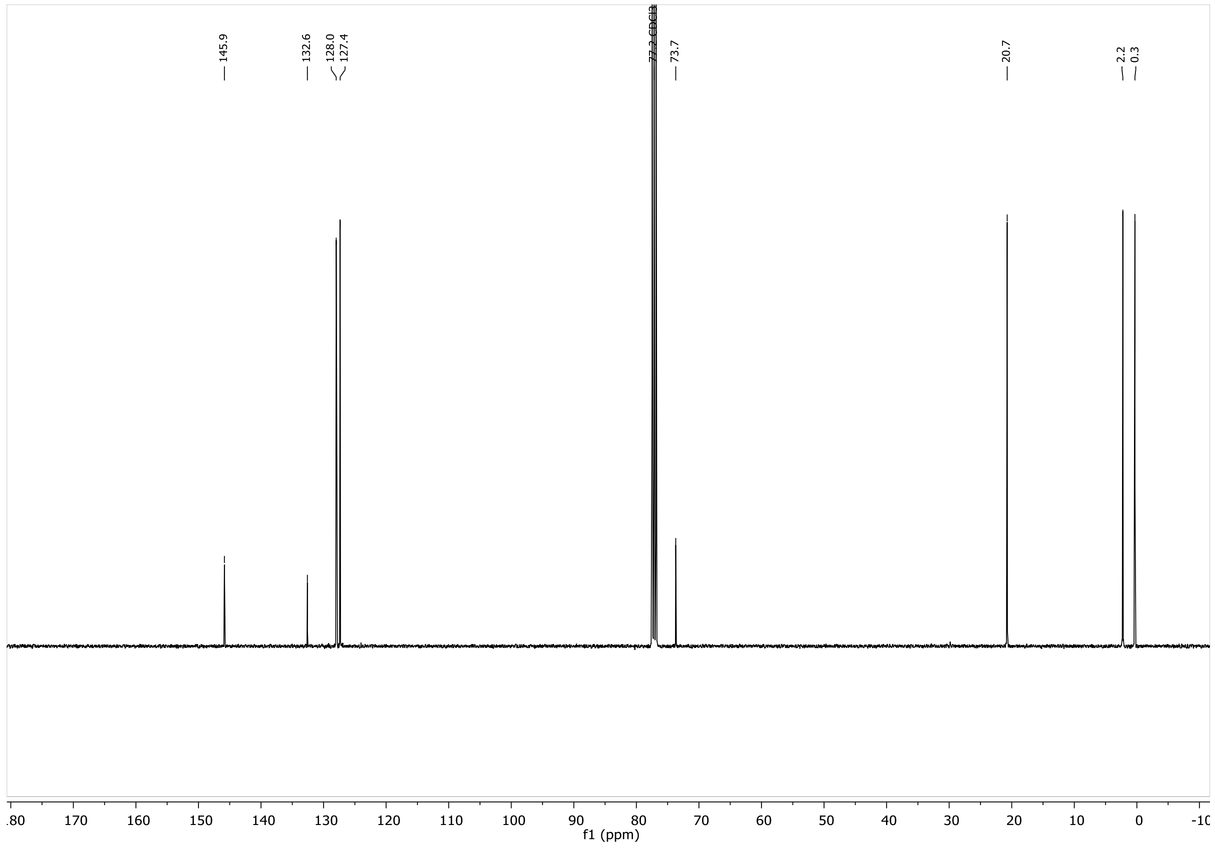

Diphenyl(2-(trifluoromethyl)phenyl)methanol (6da)

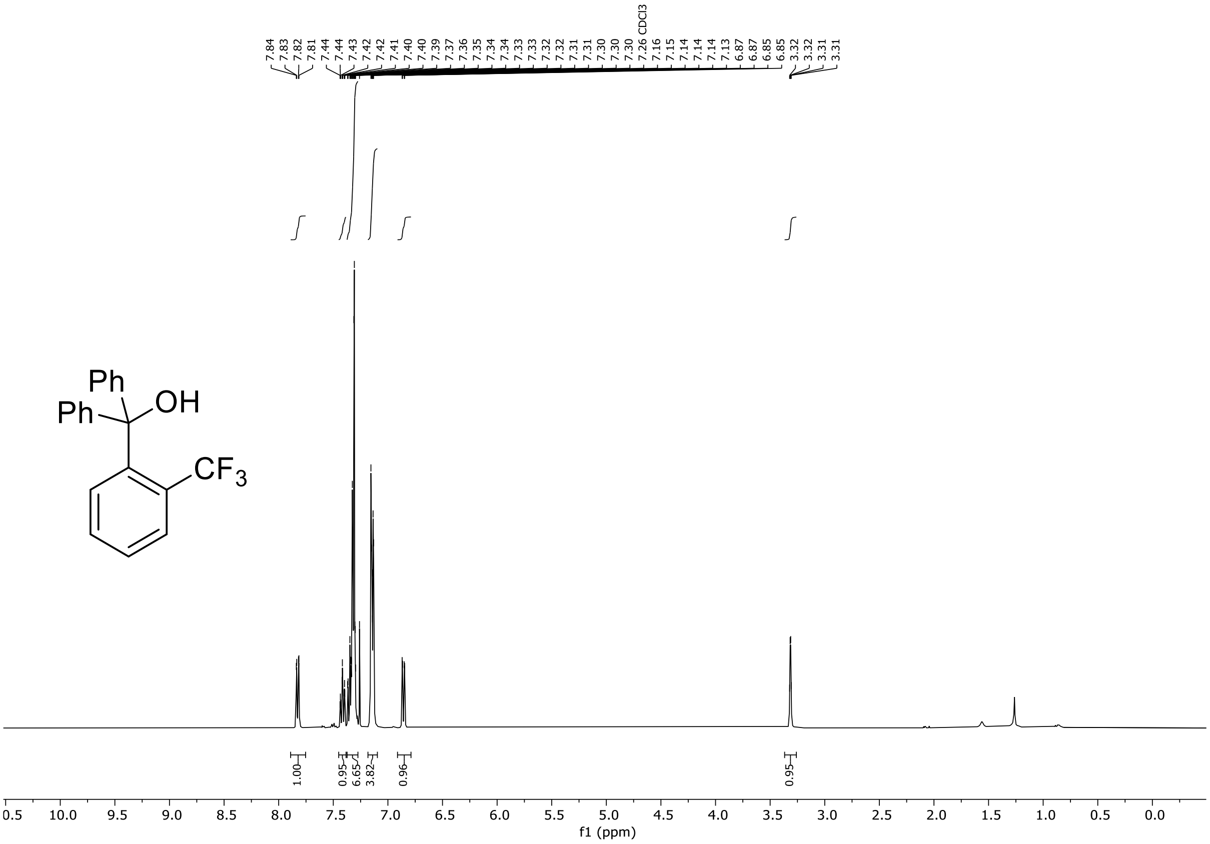

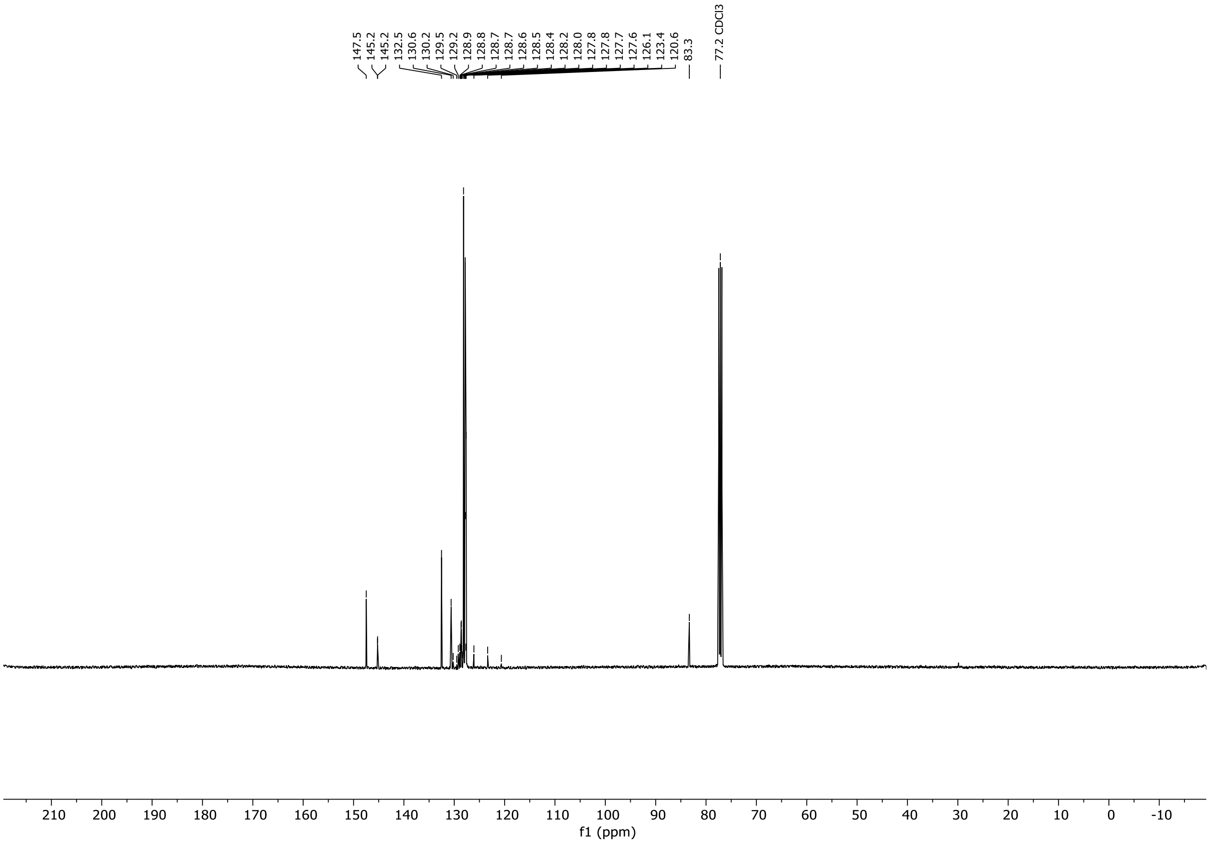

2-(2-(Trifluoromethyl)phenyl)adamantan-2-ol (6df)

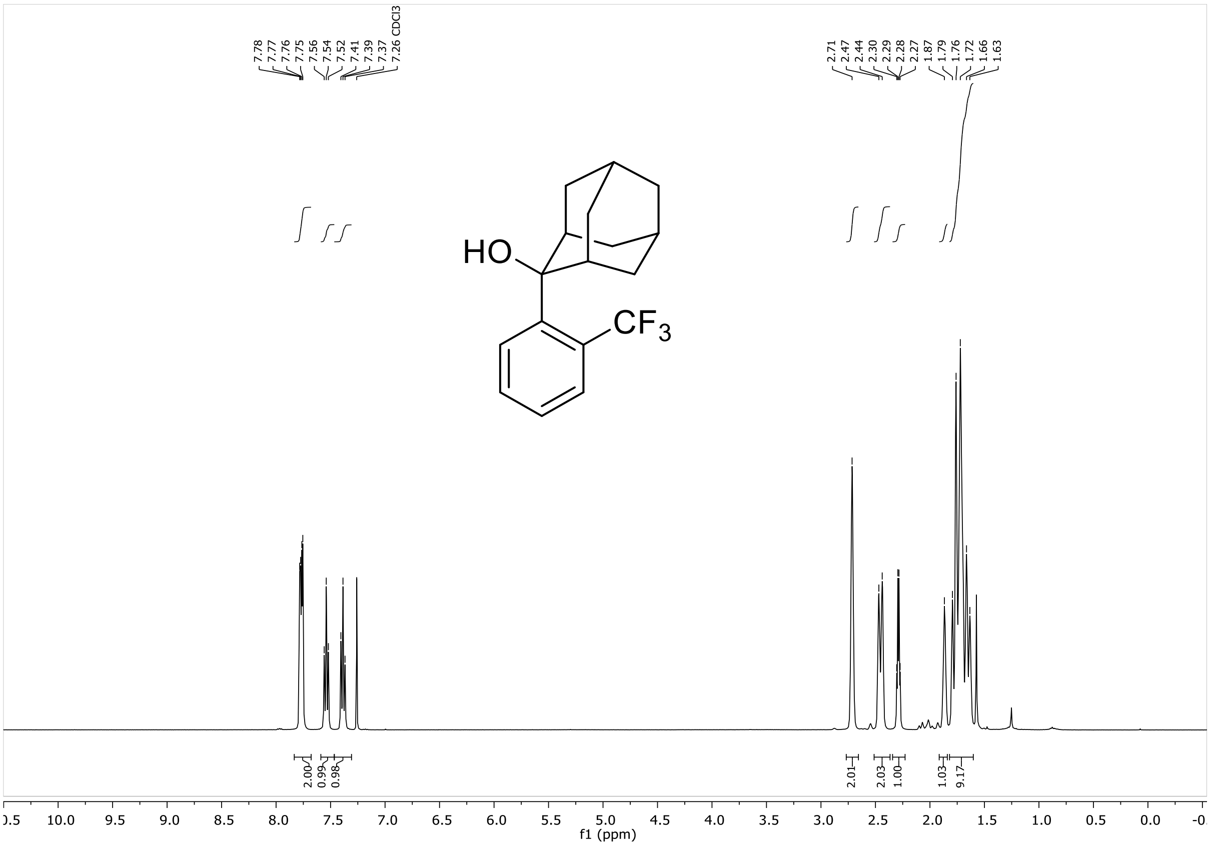

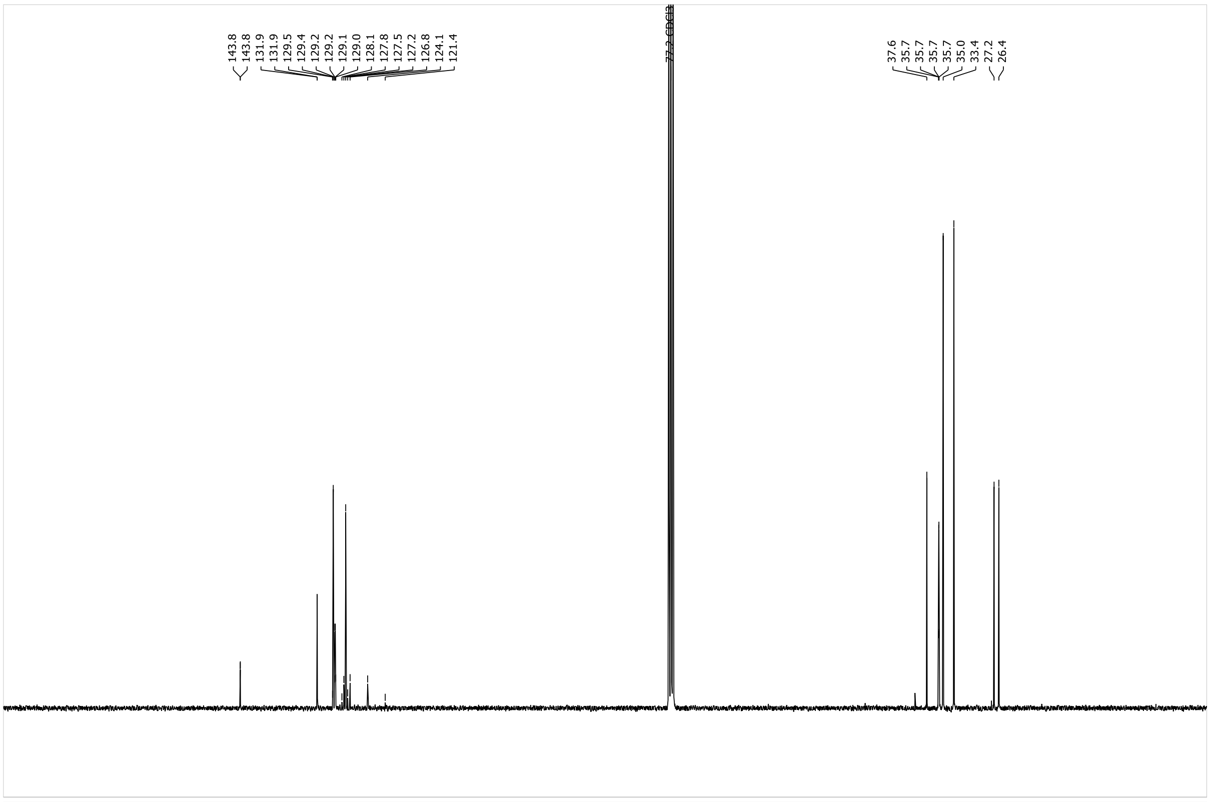

(2-Methoxyphenyl)(4-(trifluoromethyl)phenyl)methanone (6ec)

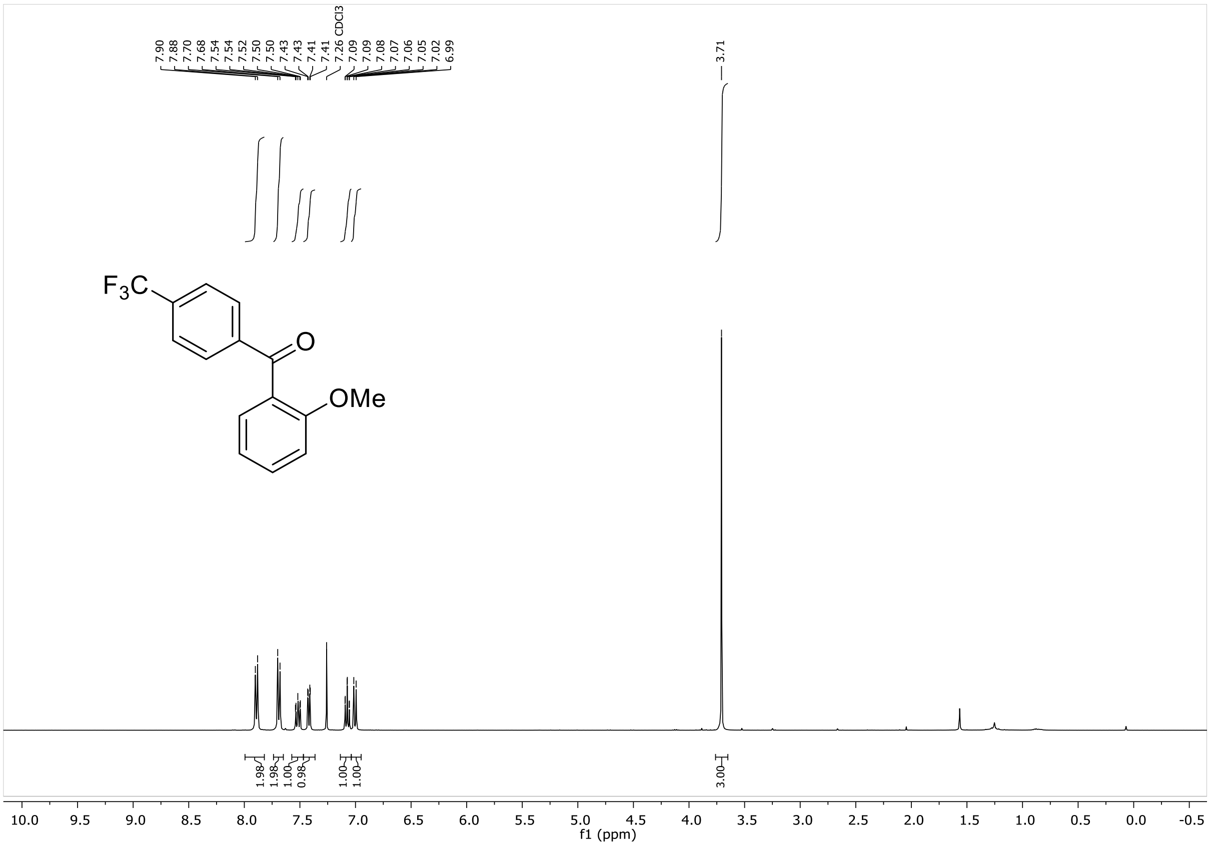

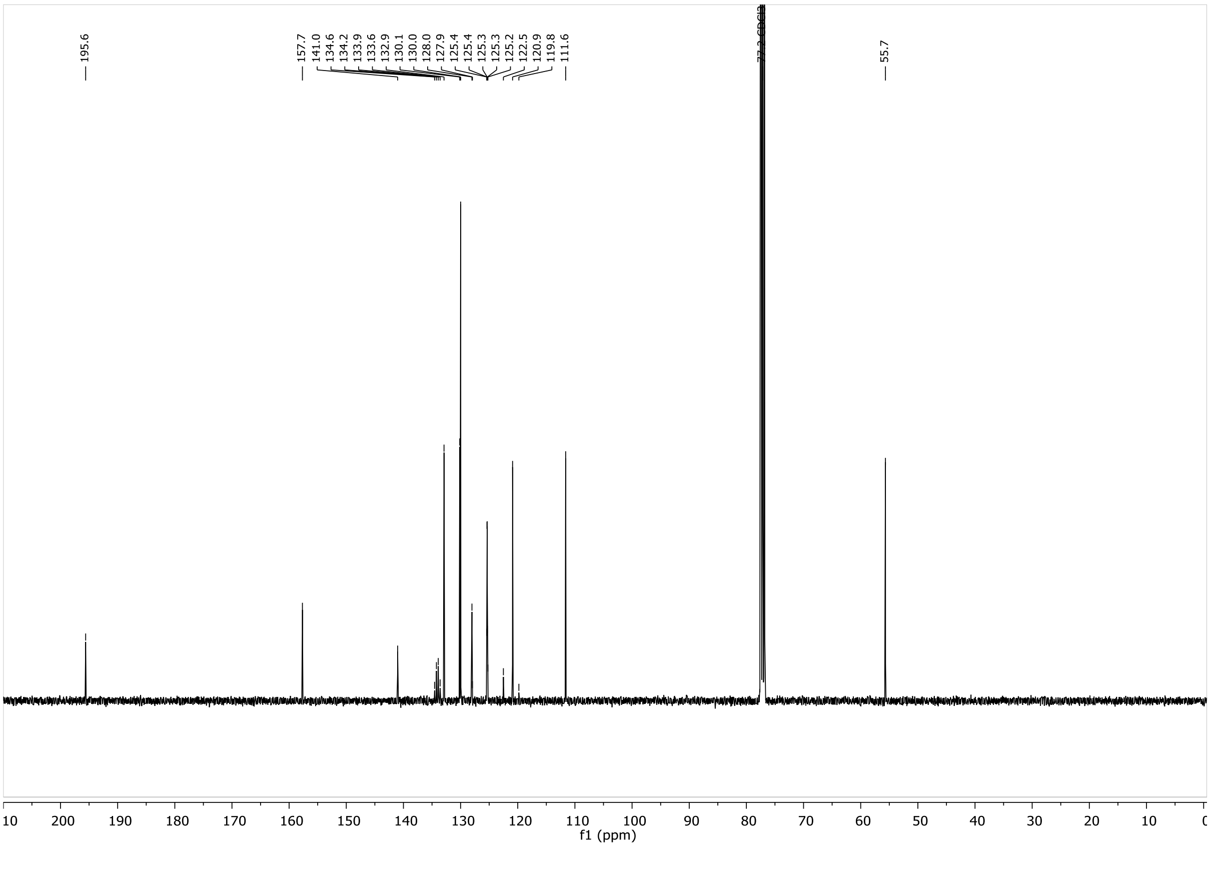

(2,6-Dichlorophenyl)(2-methoxyphenyl)methanol (6eg)

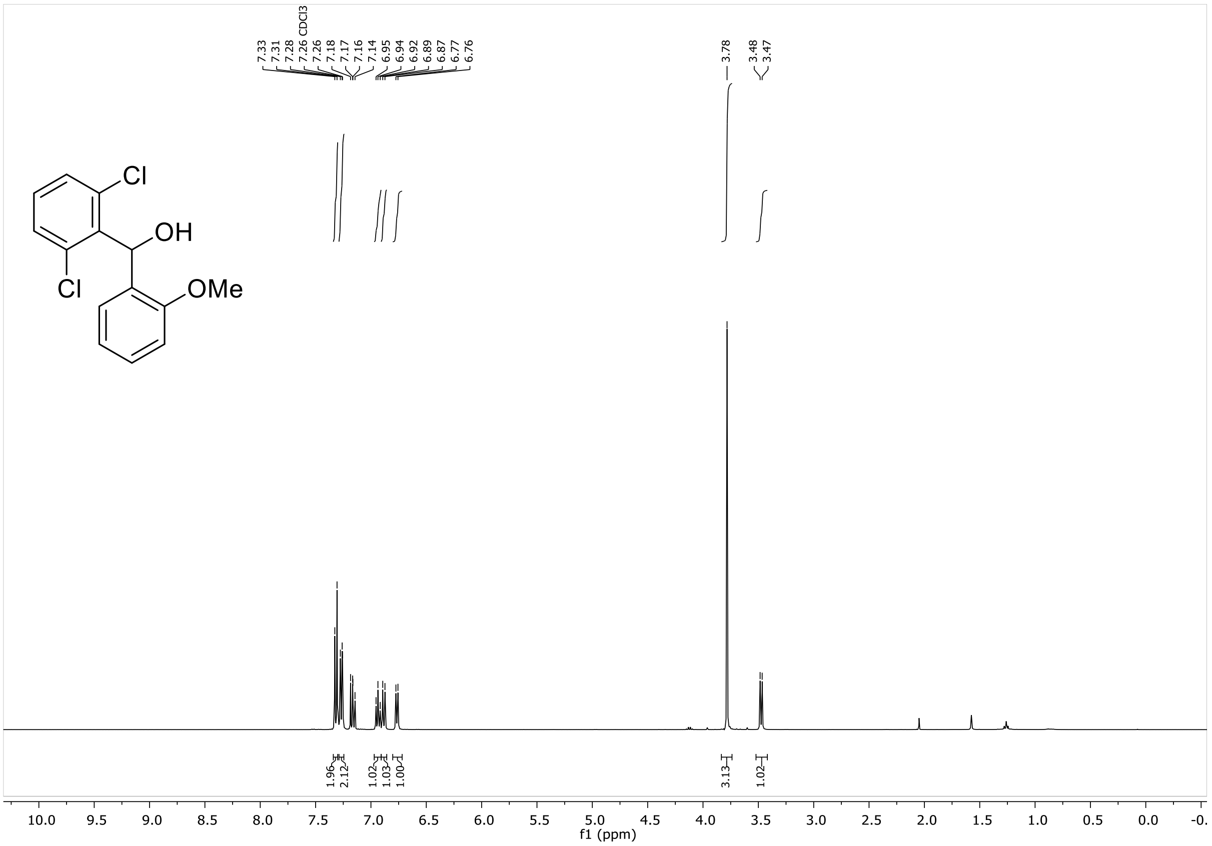

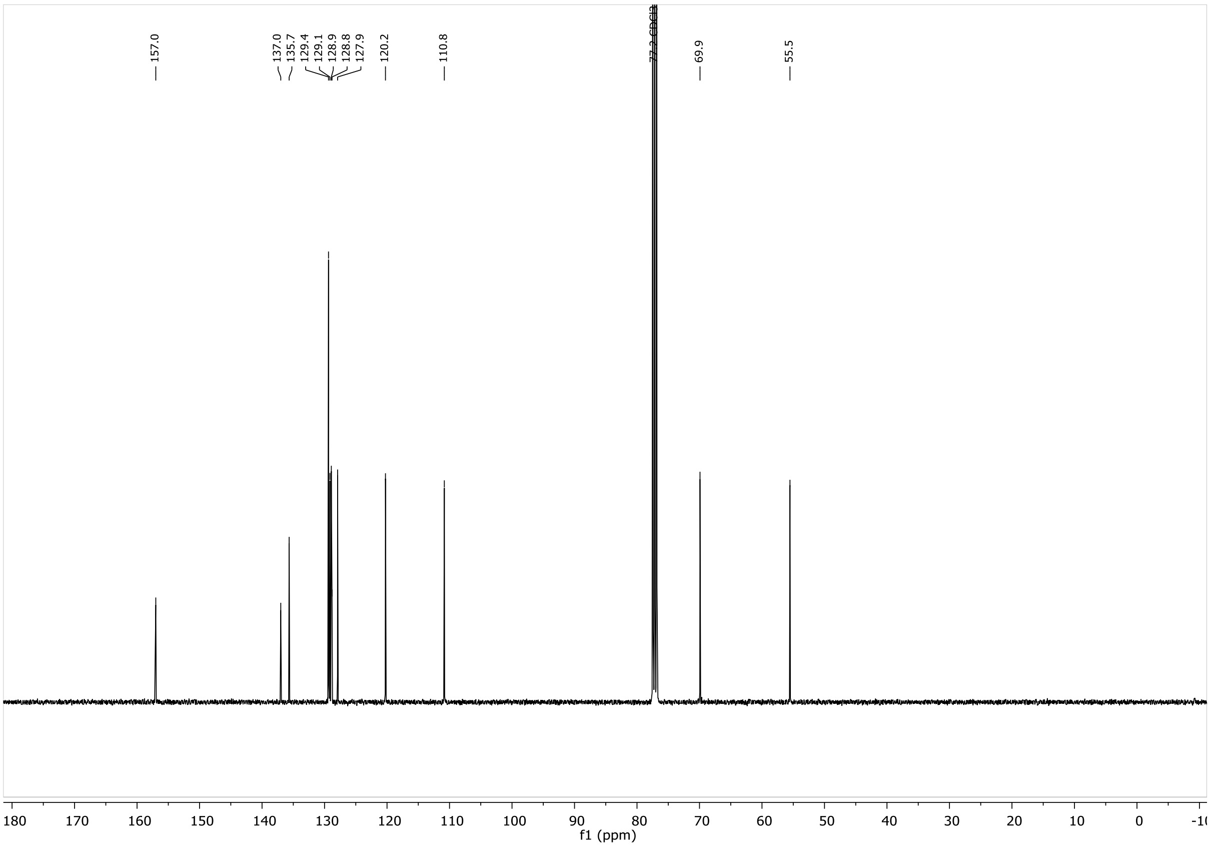

(4-Methoxy-3,5-dimethylphenyl)(thiophen-2-yl)methanone (6fh)

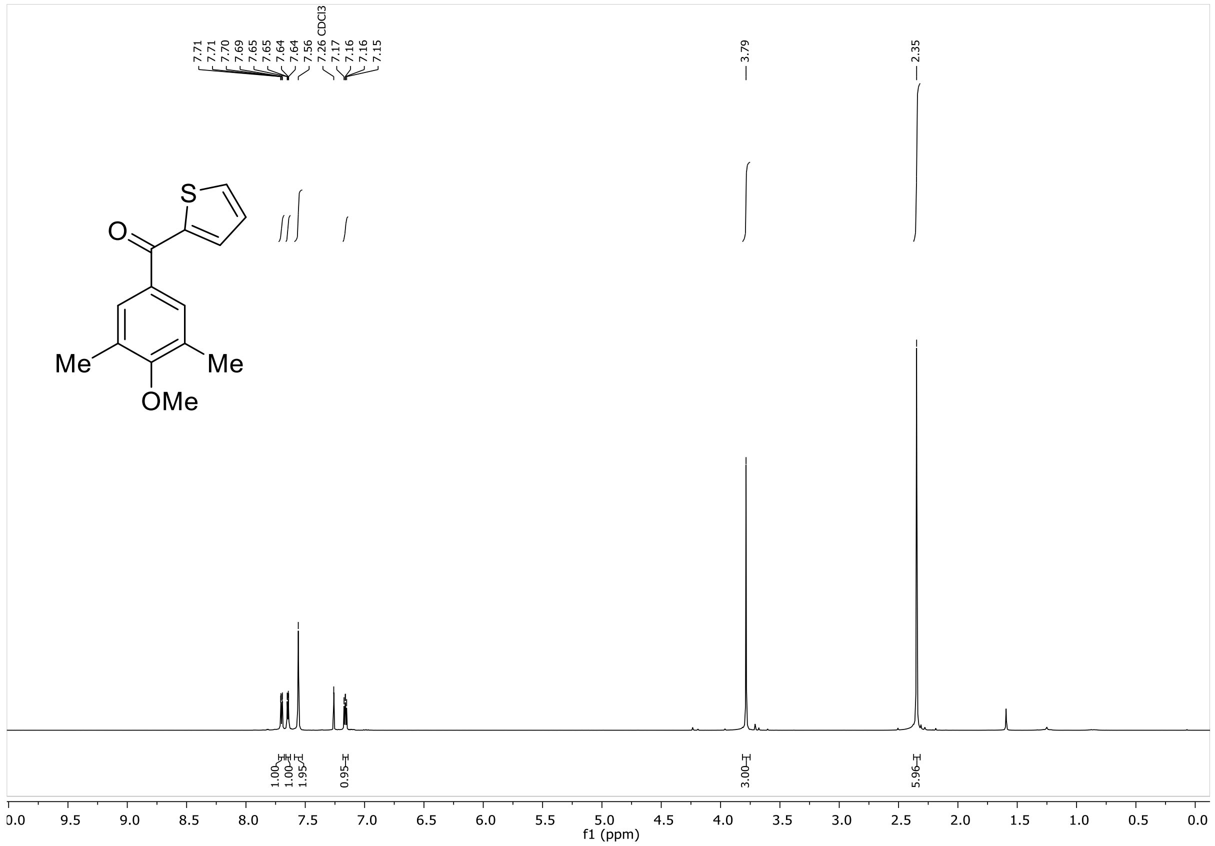

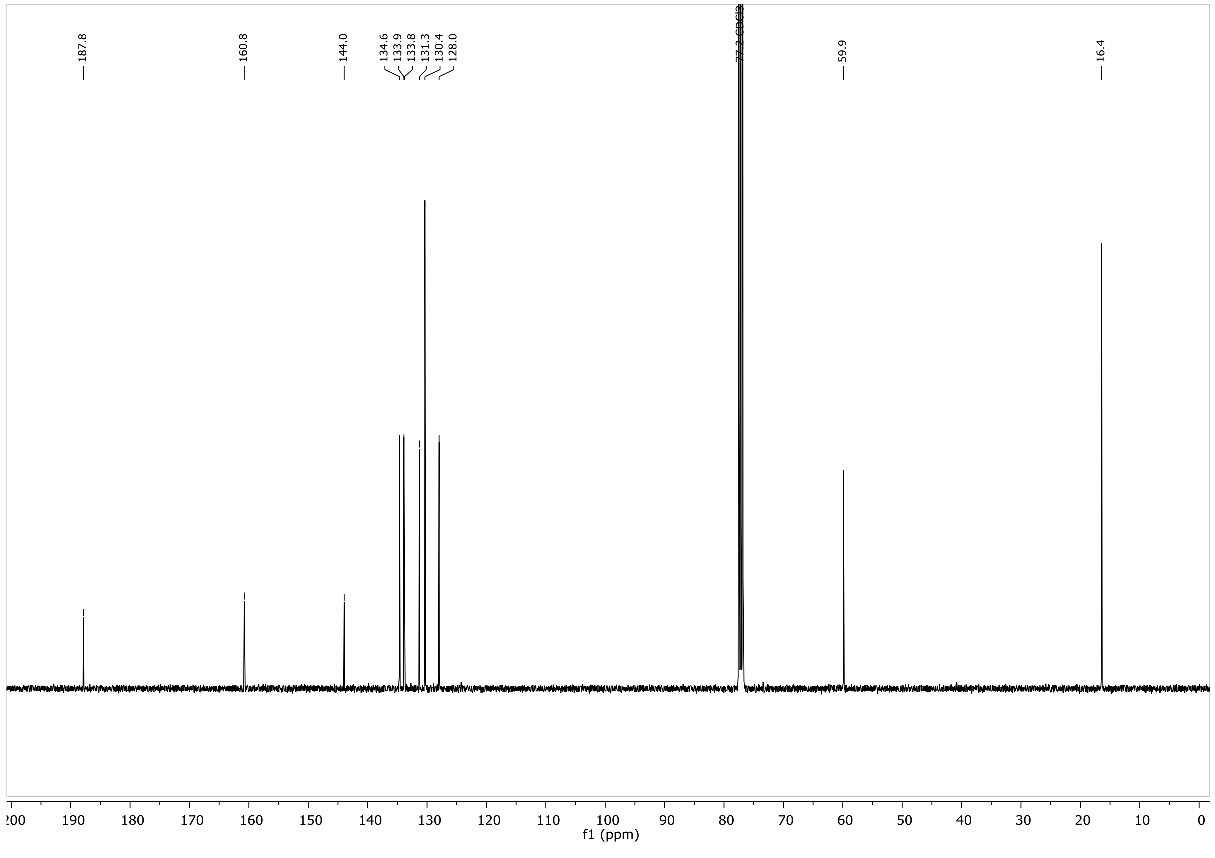

4'-Methoxy-3',5'-dimethyl-1,2,3,4-tetrahydro-1,1'-biphenyl (6fi)

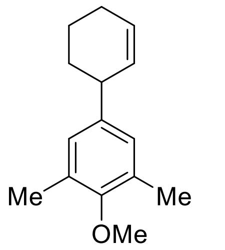

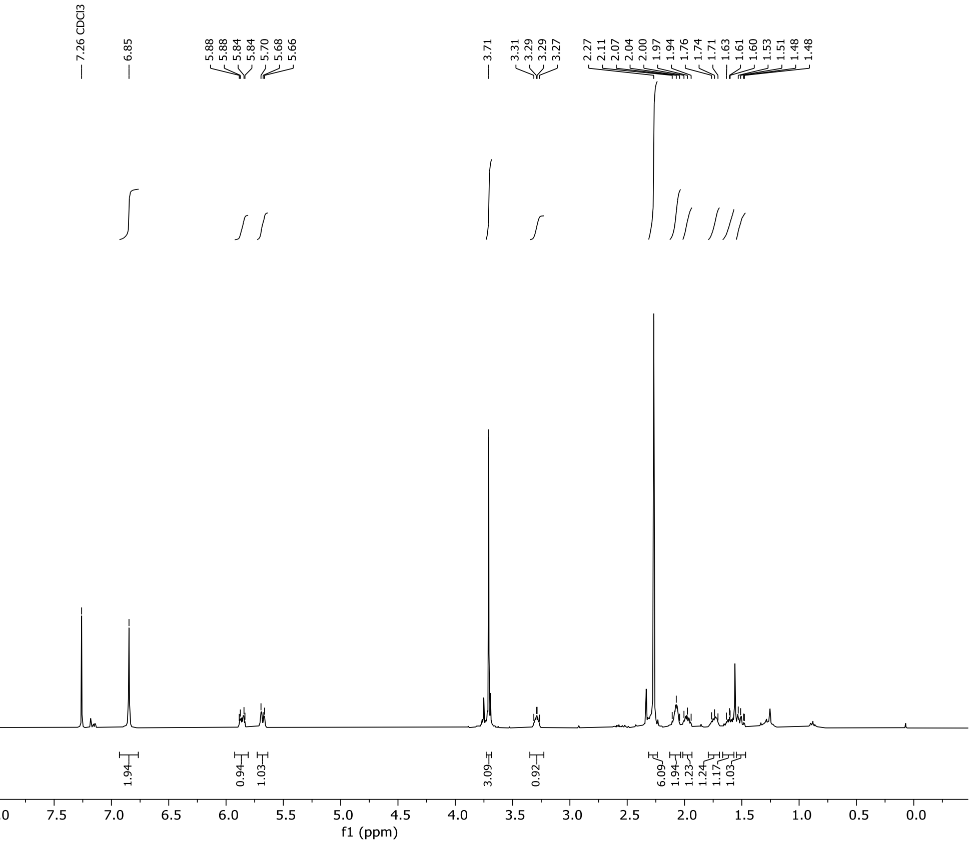

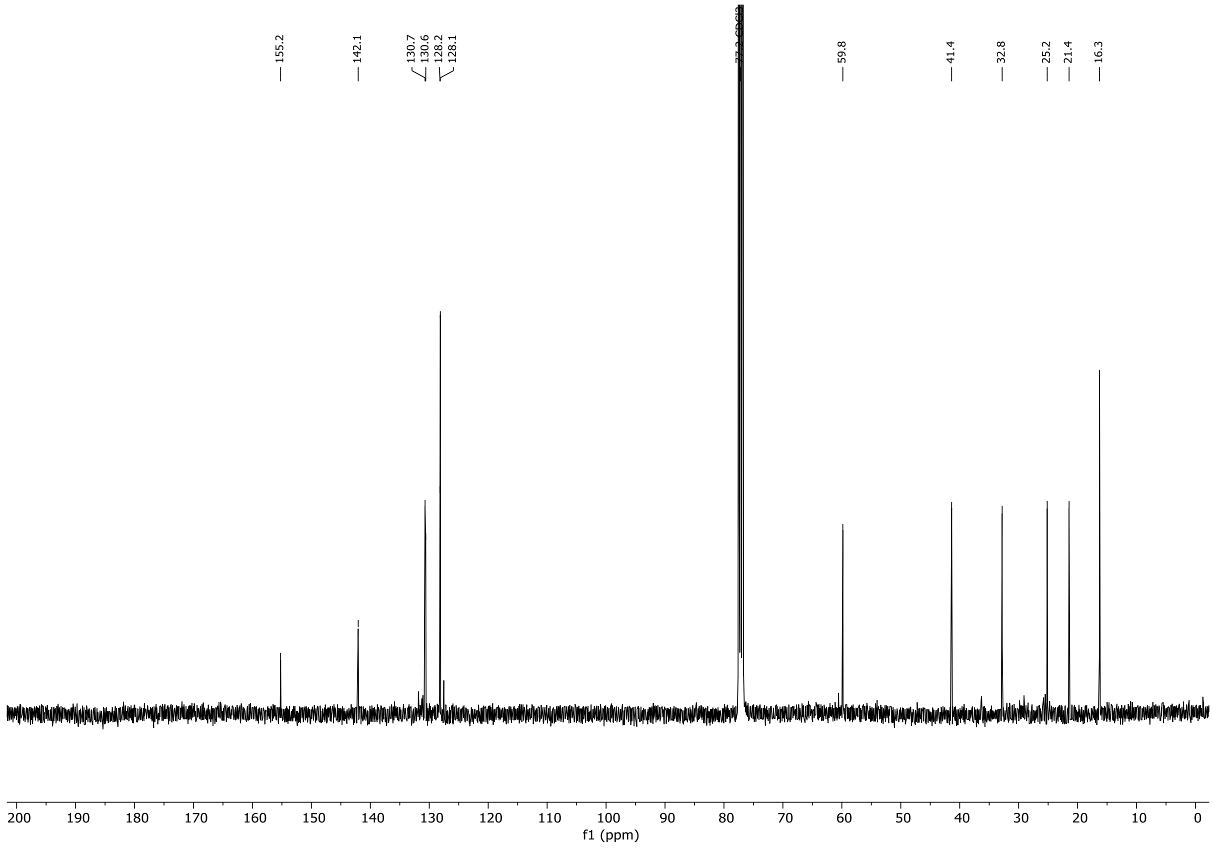

Cyclohexyl(4-methoxy-3,5-dimethylphenyl)(phenyl)methanol (6fj)

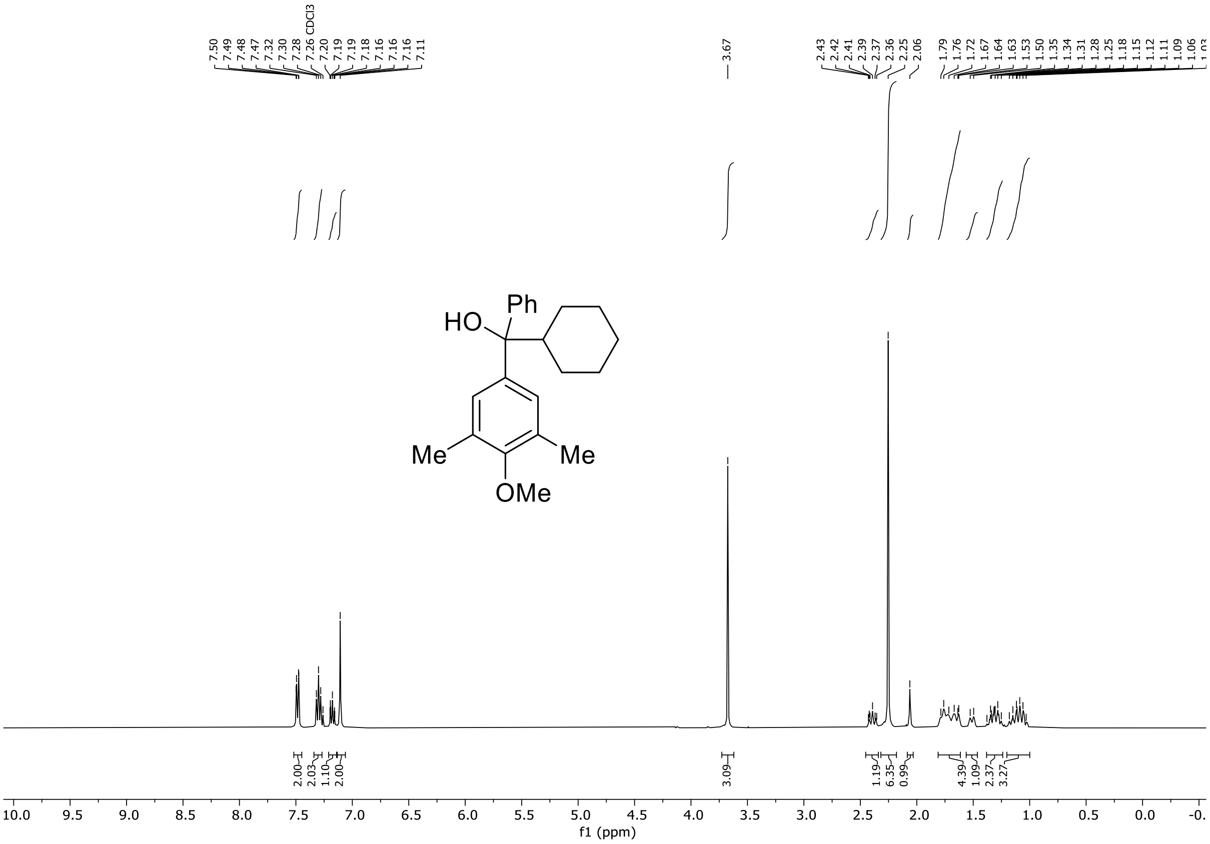

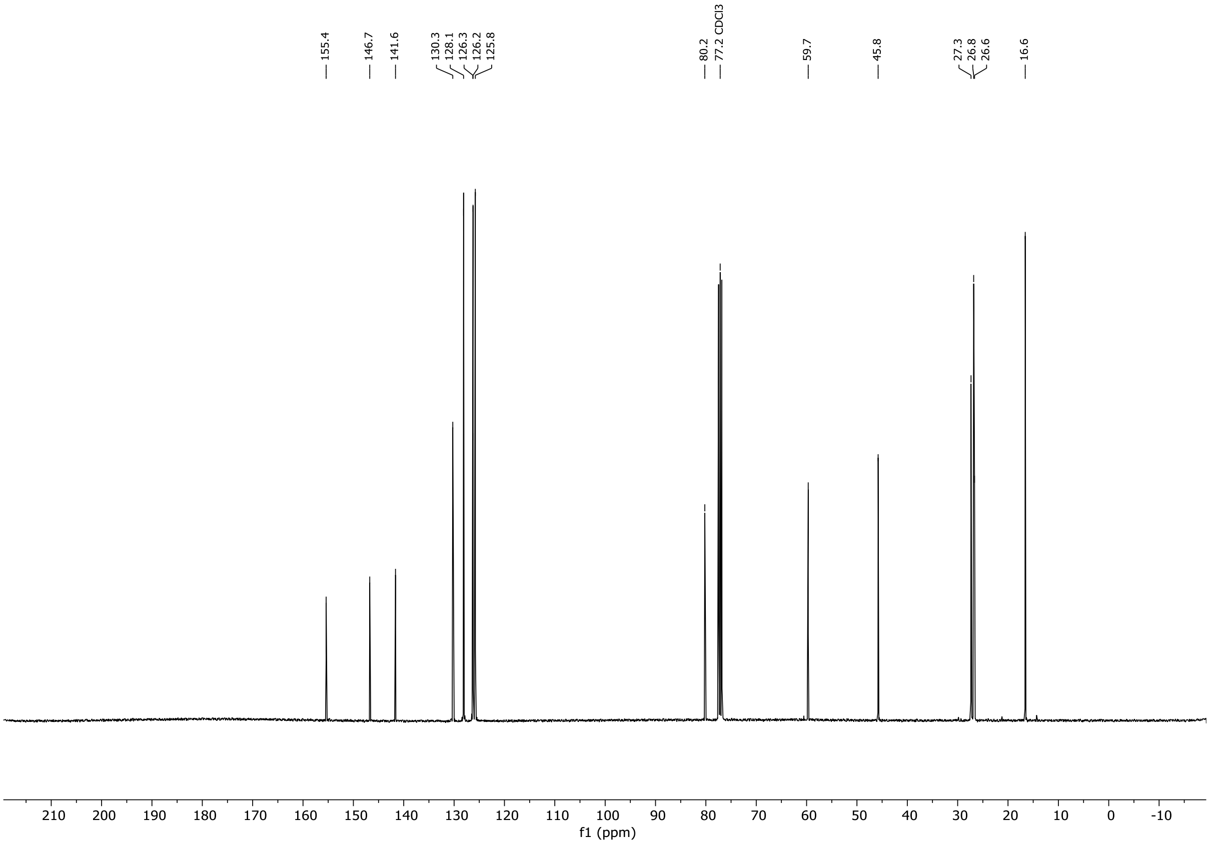

(2-(Dimethylamino)phenyl)(phenyl)(pyridin-4-yl)methanol (6gk)

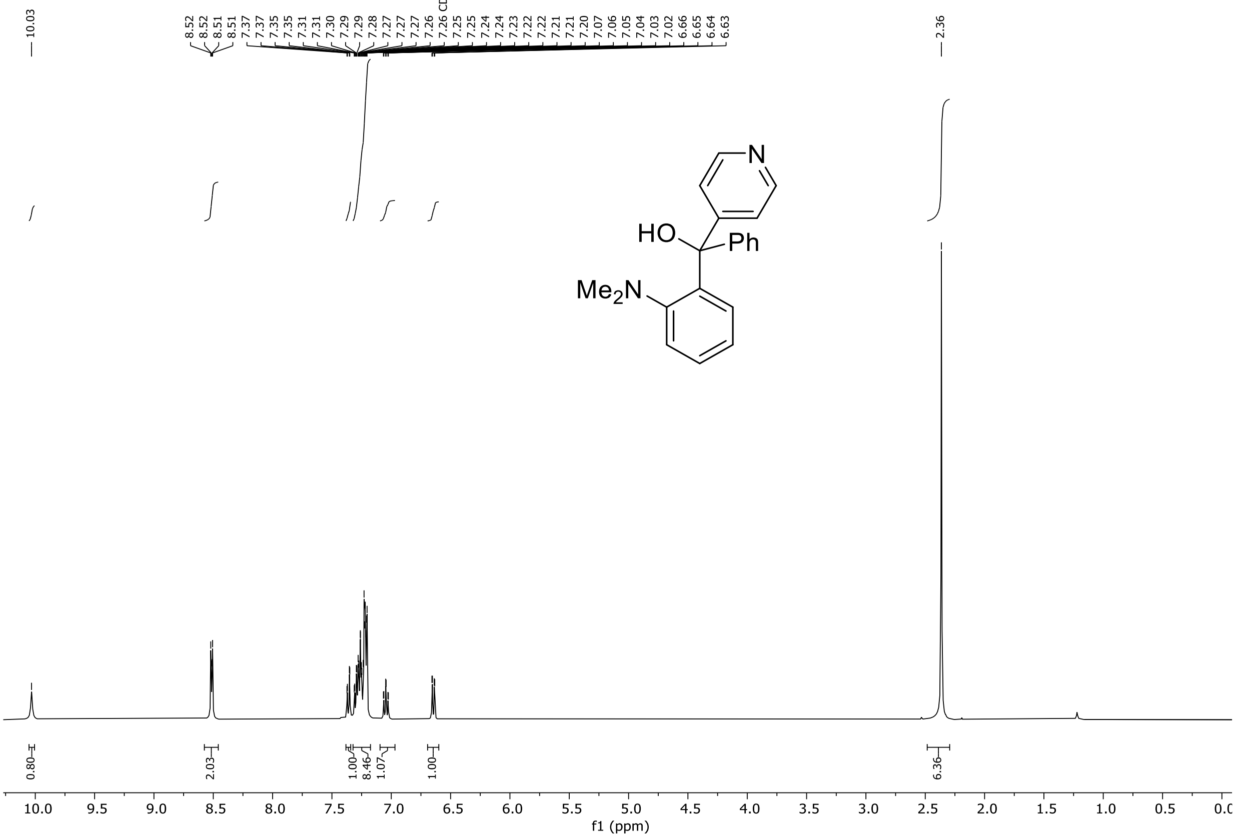

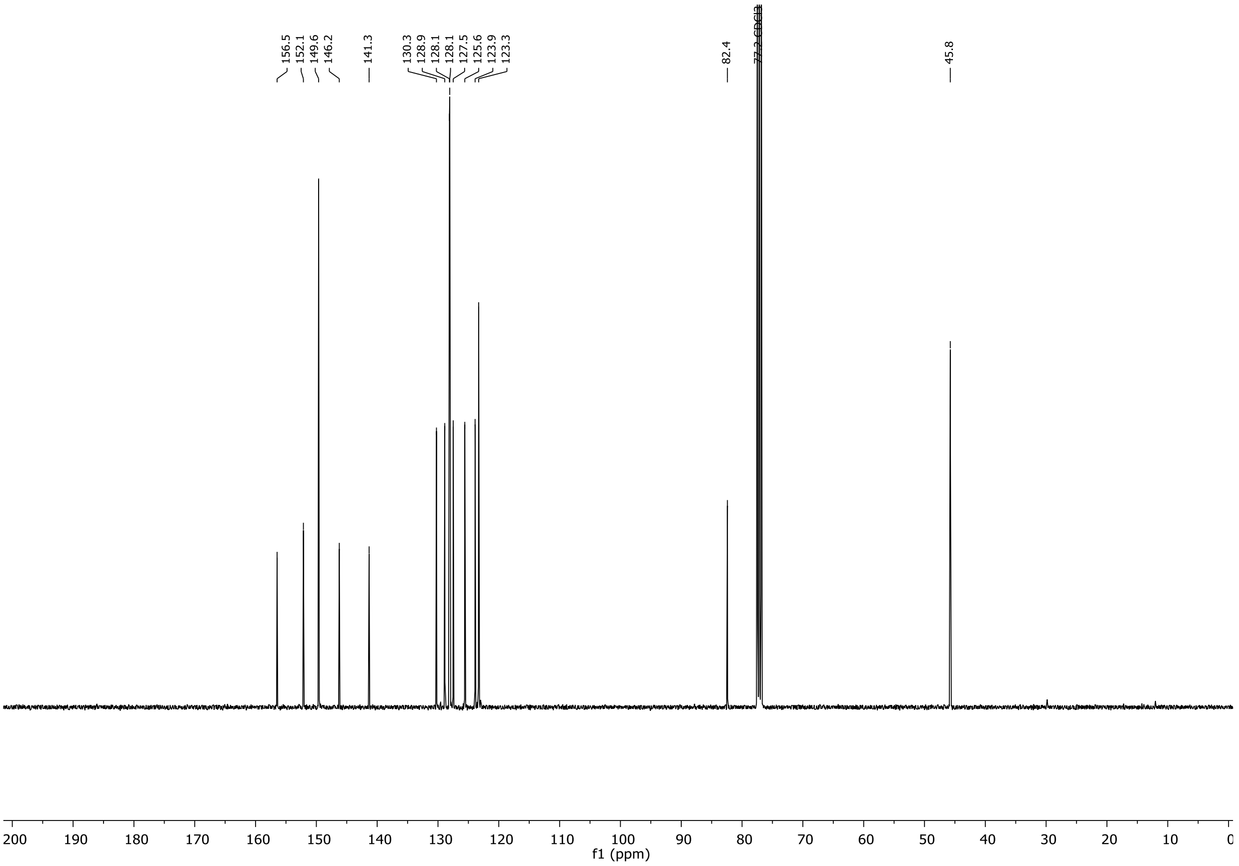

***N,N*-Dimethyl-2-(phenyl(phenylamino)methyl)aniline (6gd)**

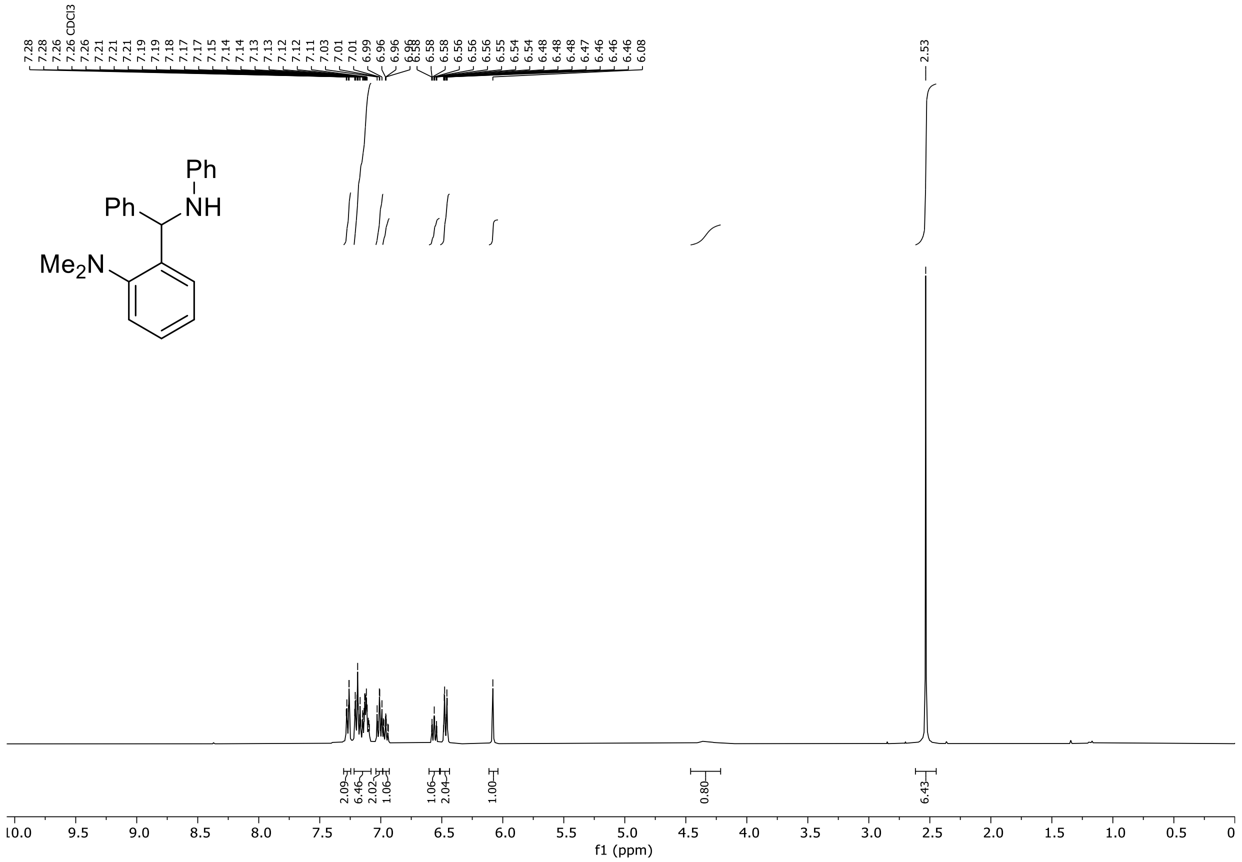

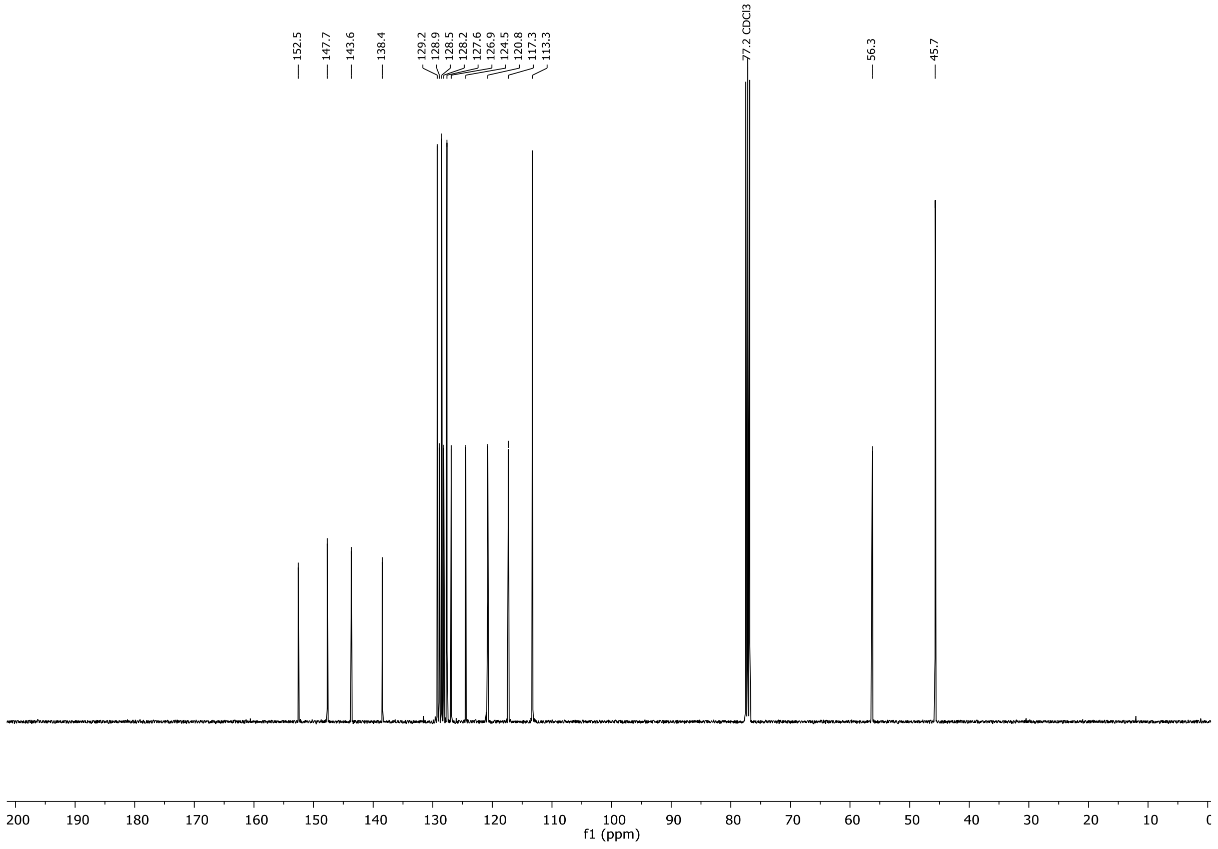

N,N-Dimethyl-2-(pyridin-2-ylthio)aniline (6gl)

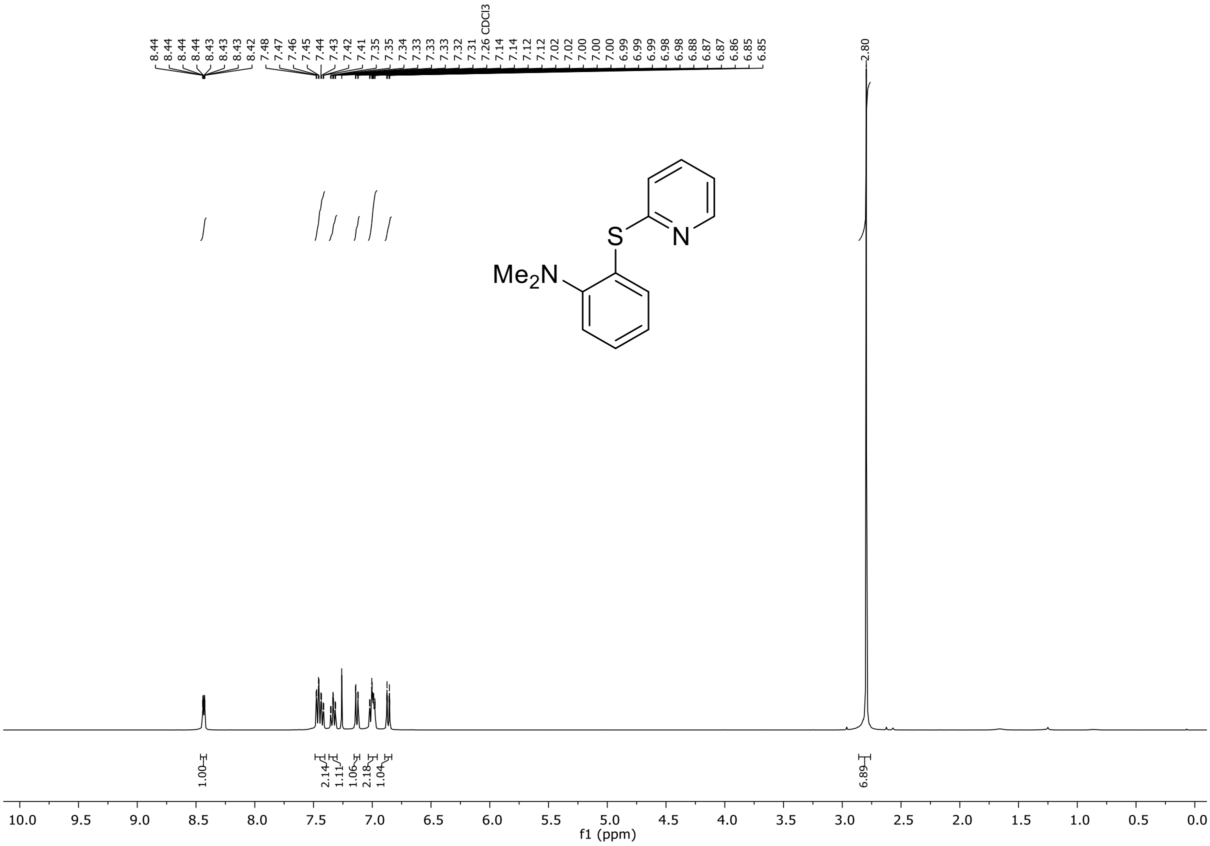

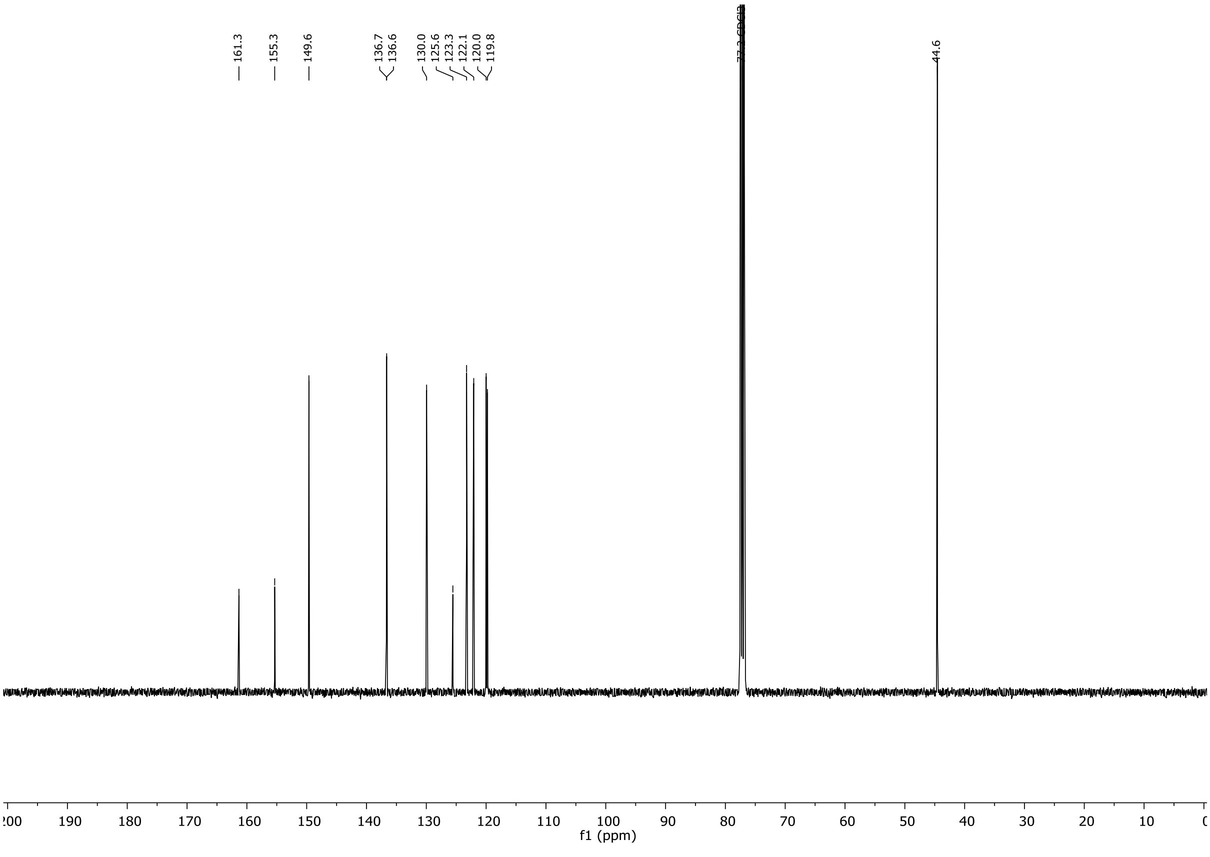

Diphenyl(pyridin-2-yl)methanol (9aa)

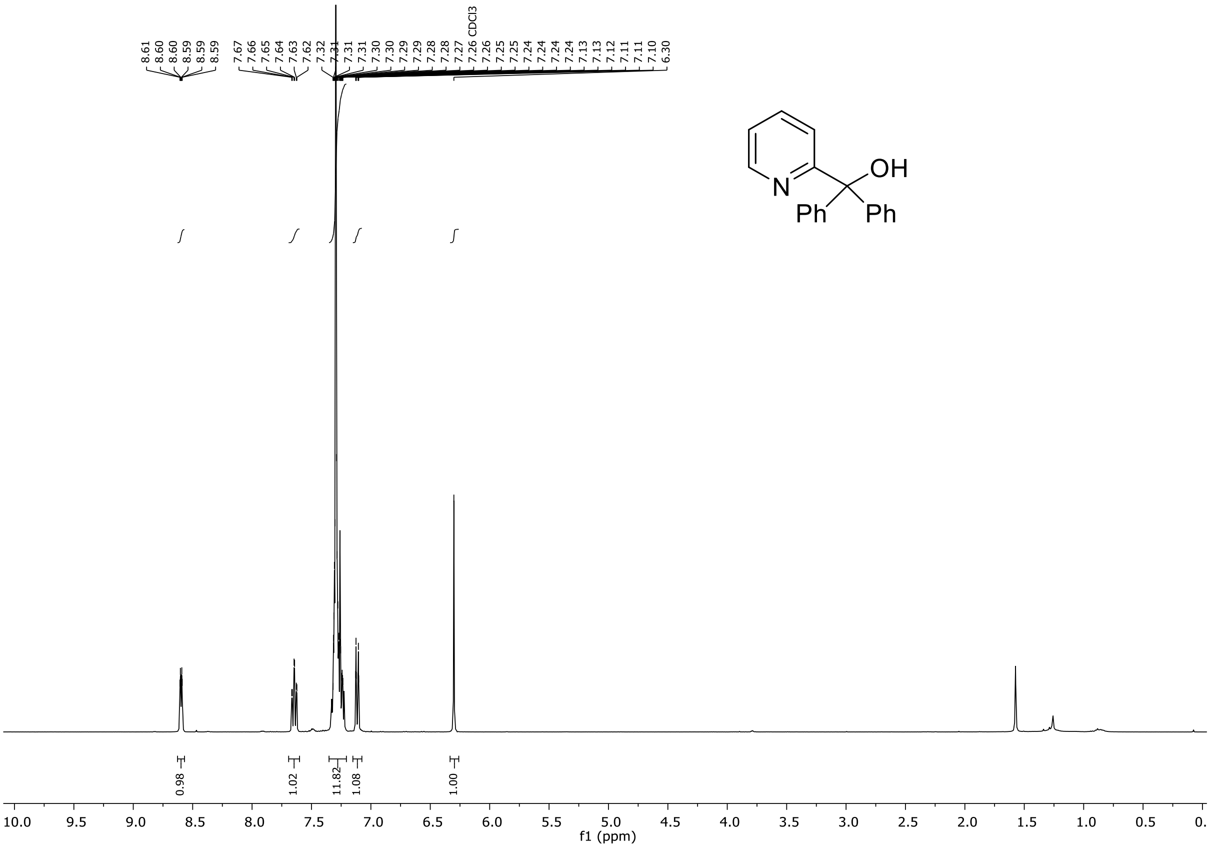

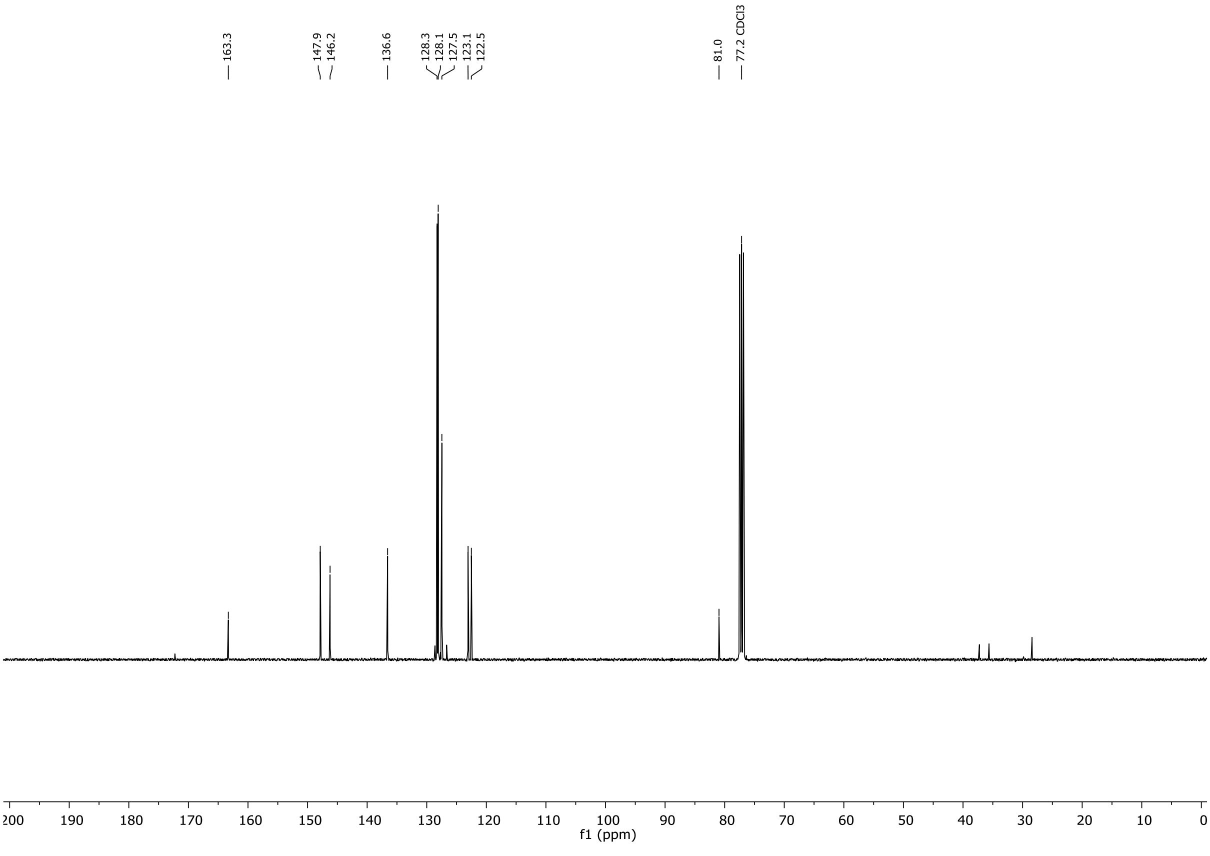

(4-Chlorophenyl)(cyclopropyl)(pyridin-2-yl)methanol (9am)

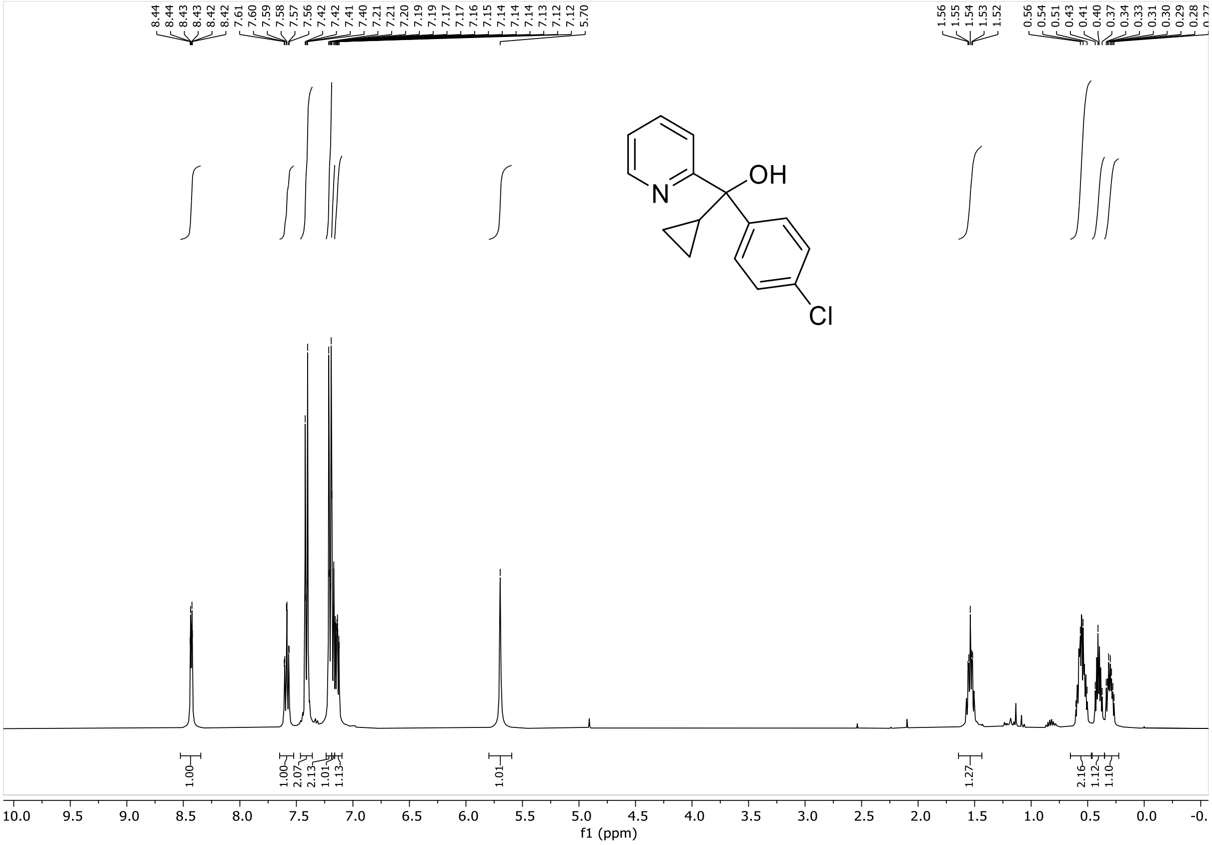

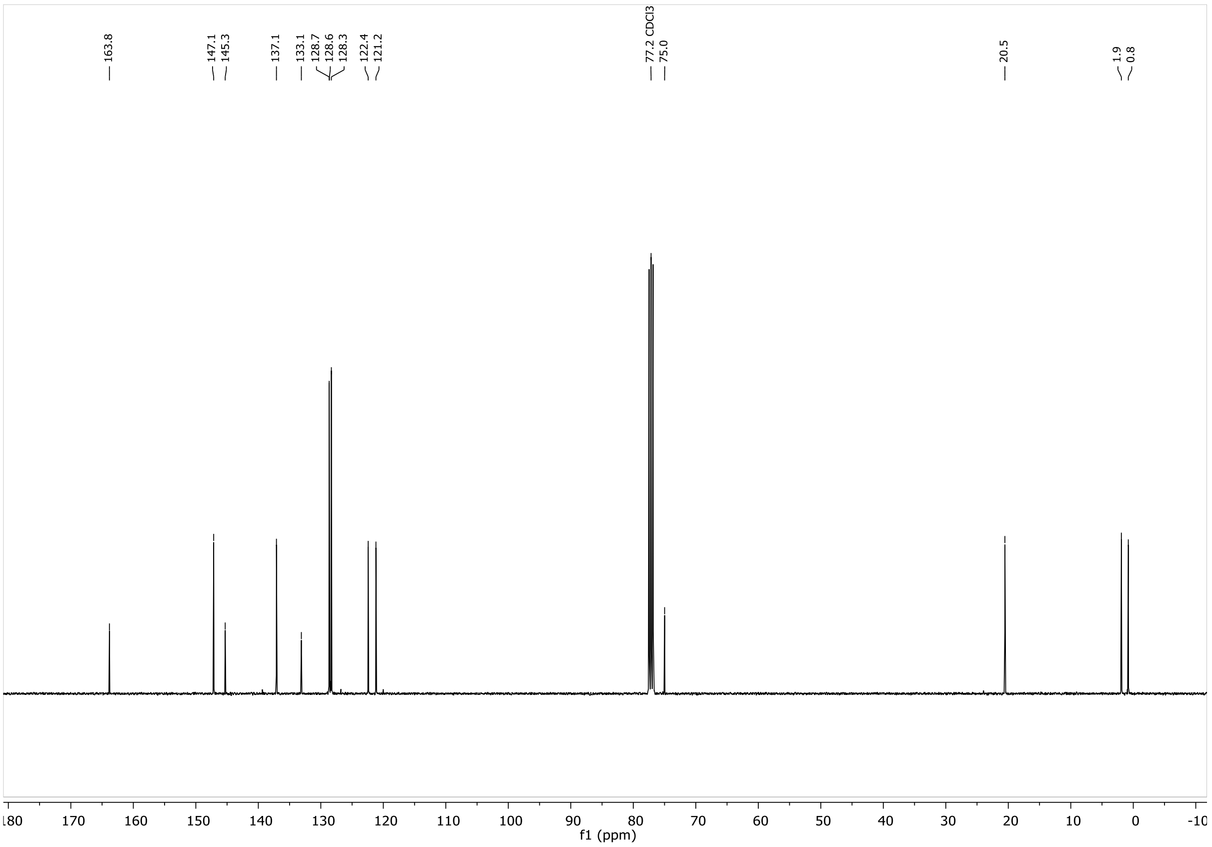

(5-Methylpyridin-2-yl)(4-(trifluoromethyl)phenyl)methanone (9bc)

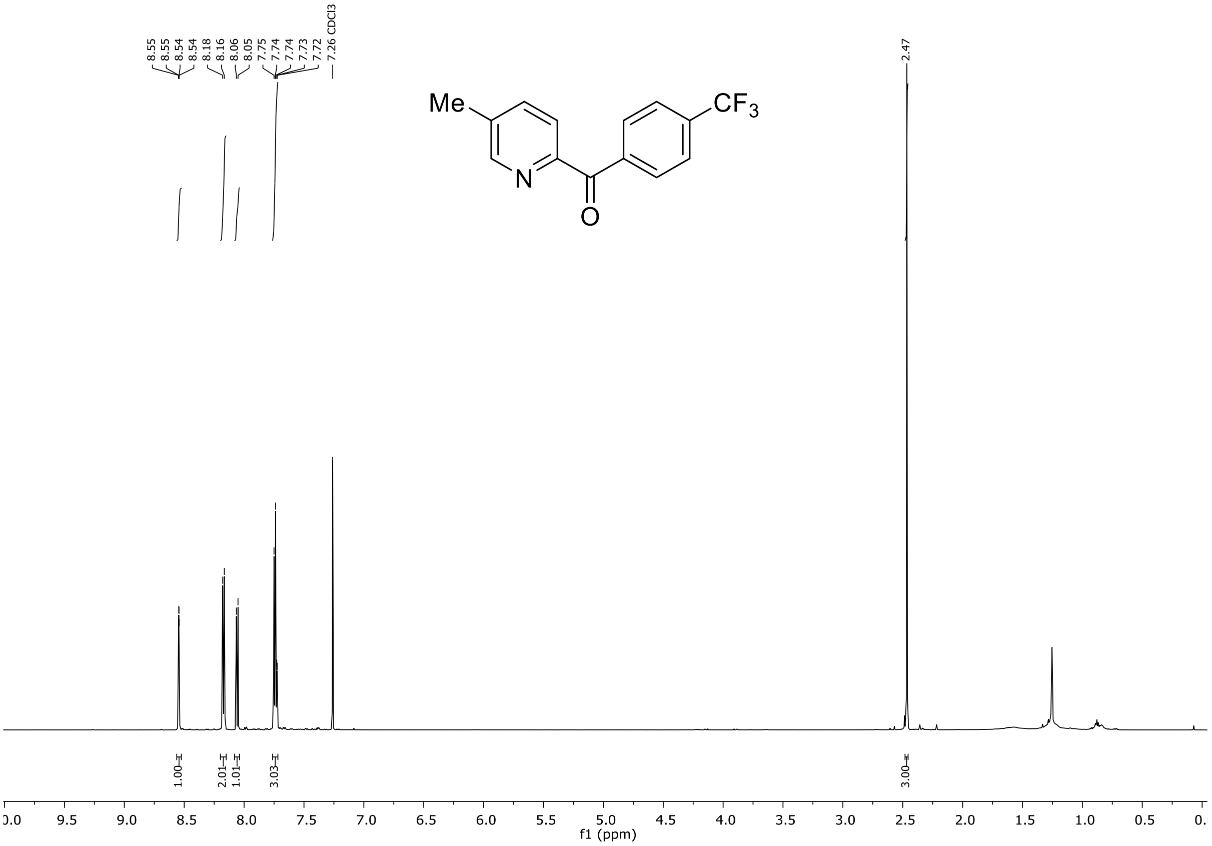

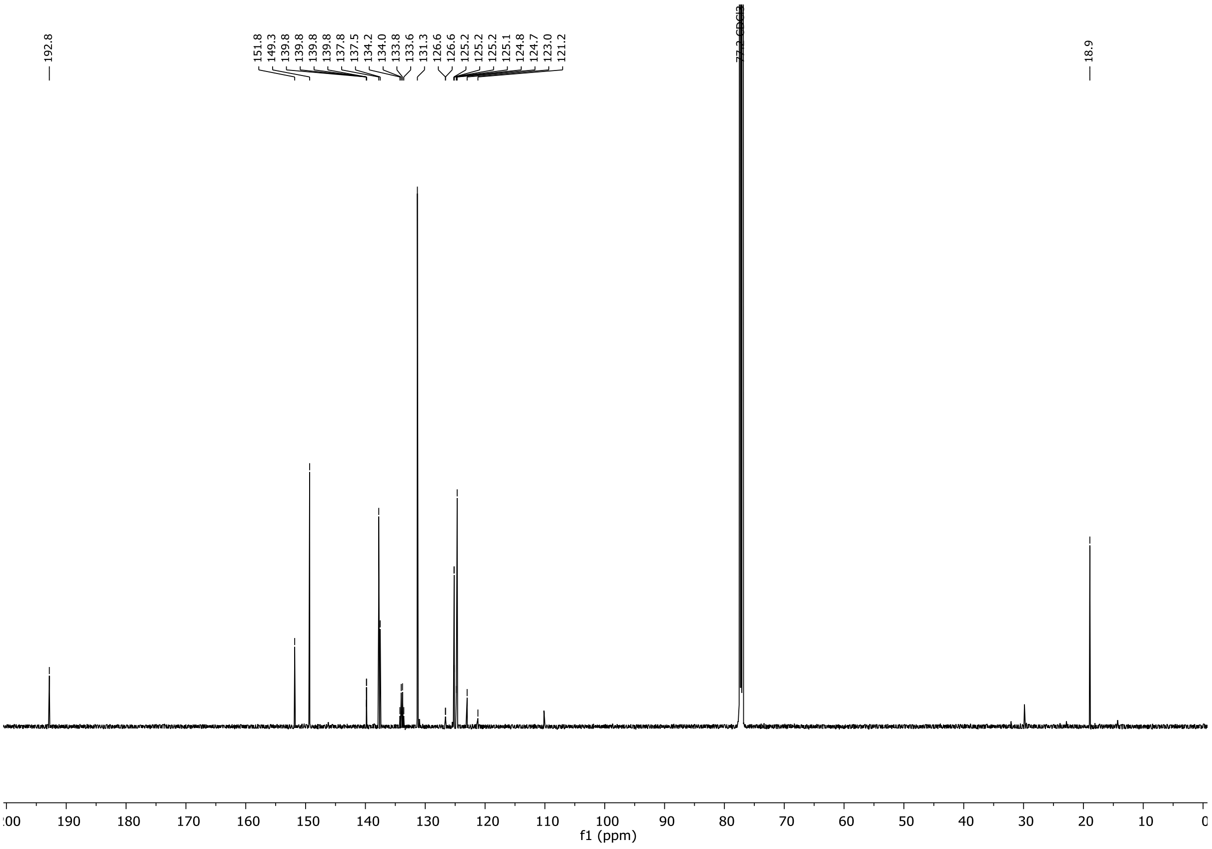

(2,6-Dichlorophenyl)(2,4-dimethoxypyrimidin-5-yl)methanol (9cg)

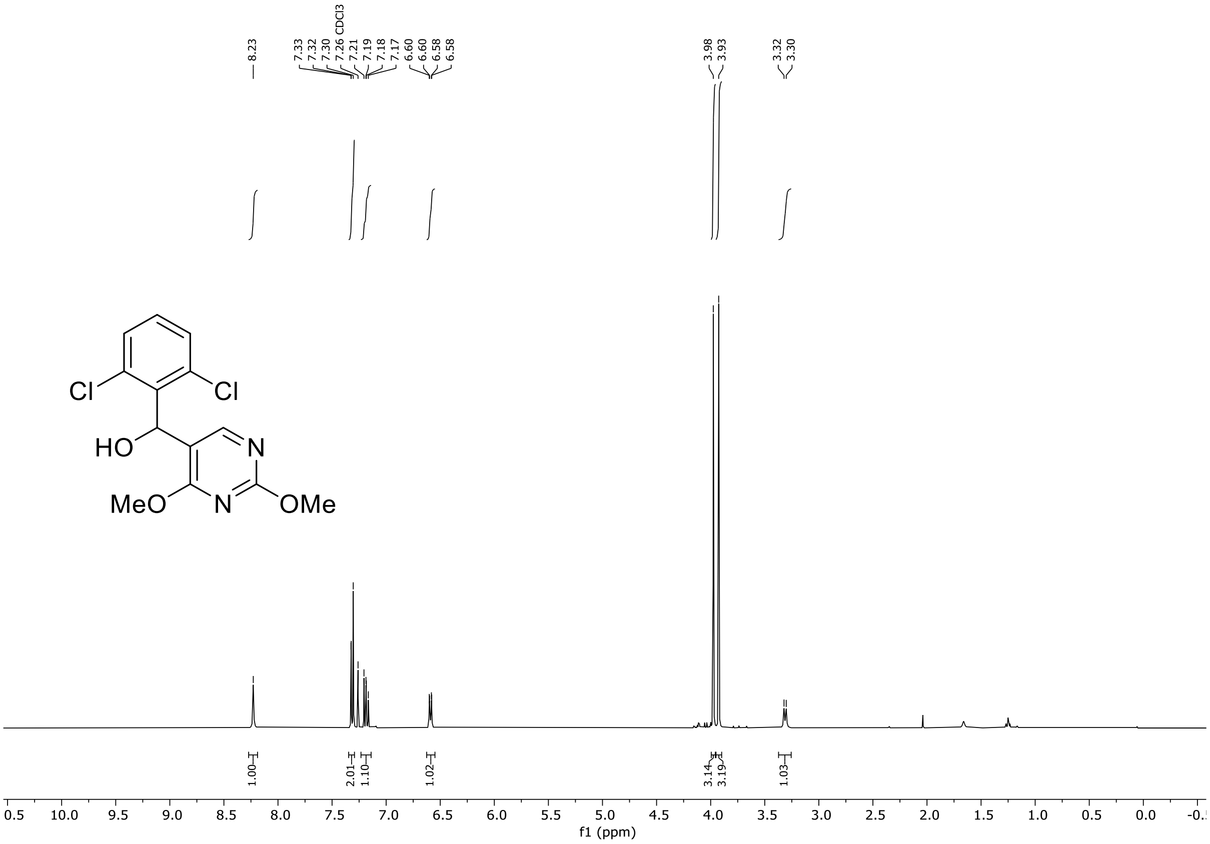

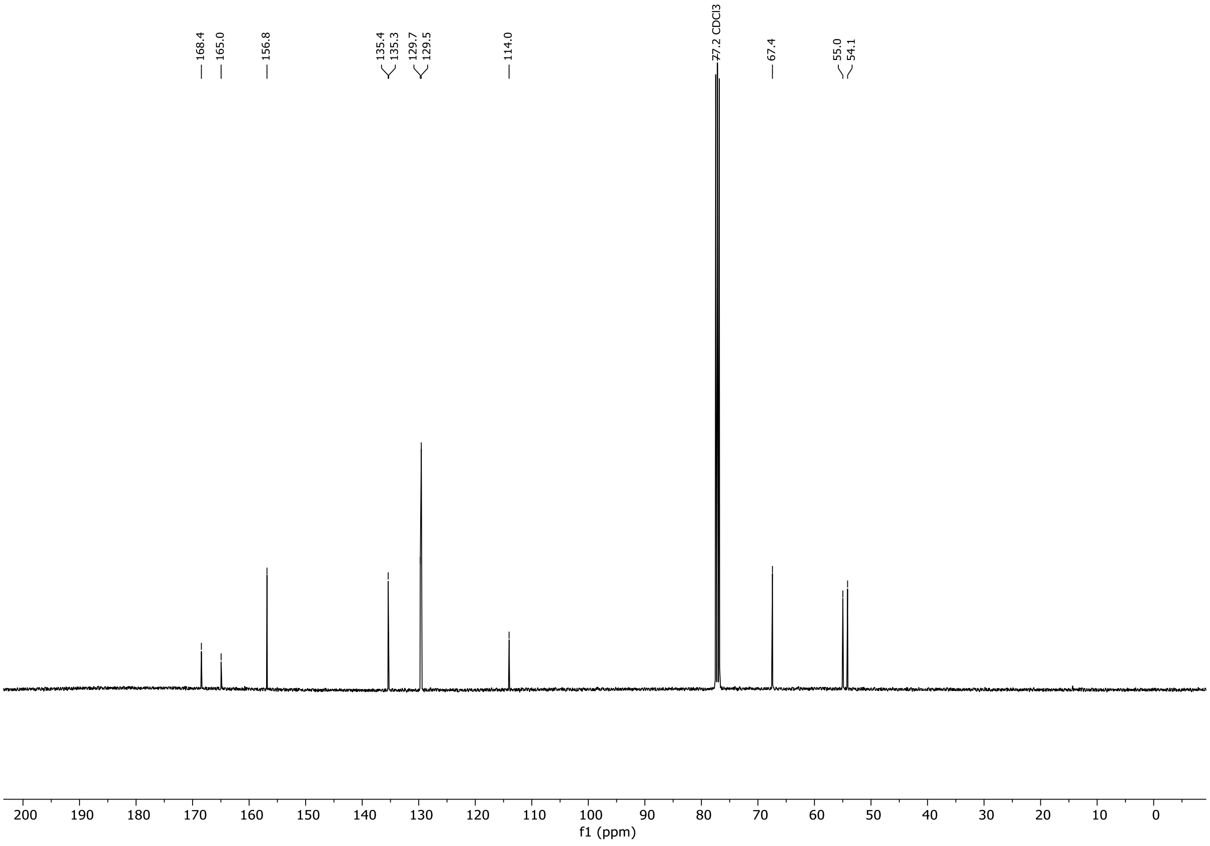

1-(2,4-Dimethoxypyrimidin-5-yl)-1-phenylethan-1-ol (9cn)

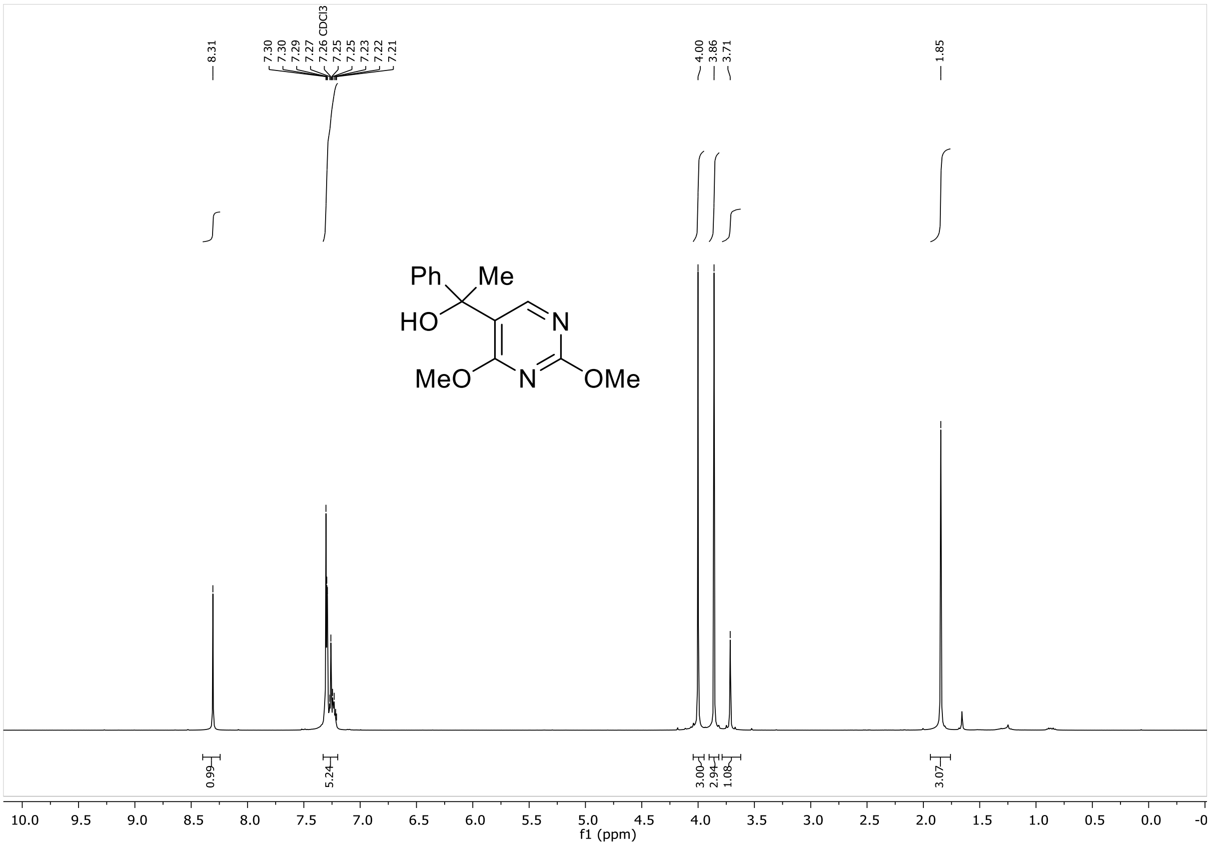

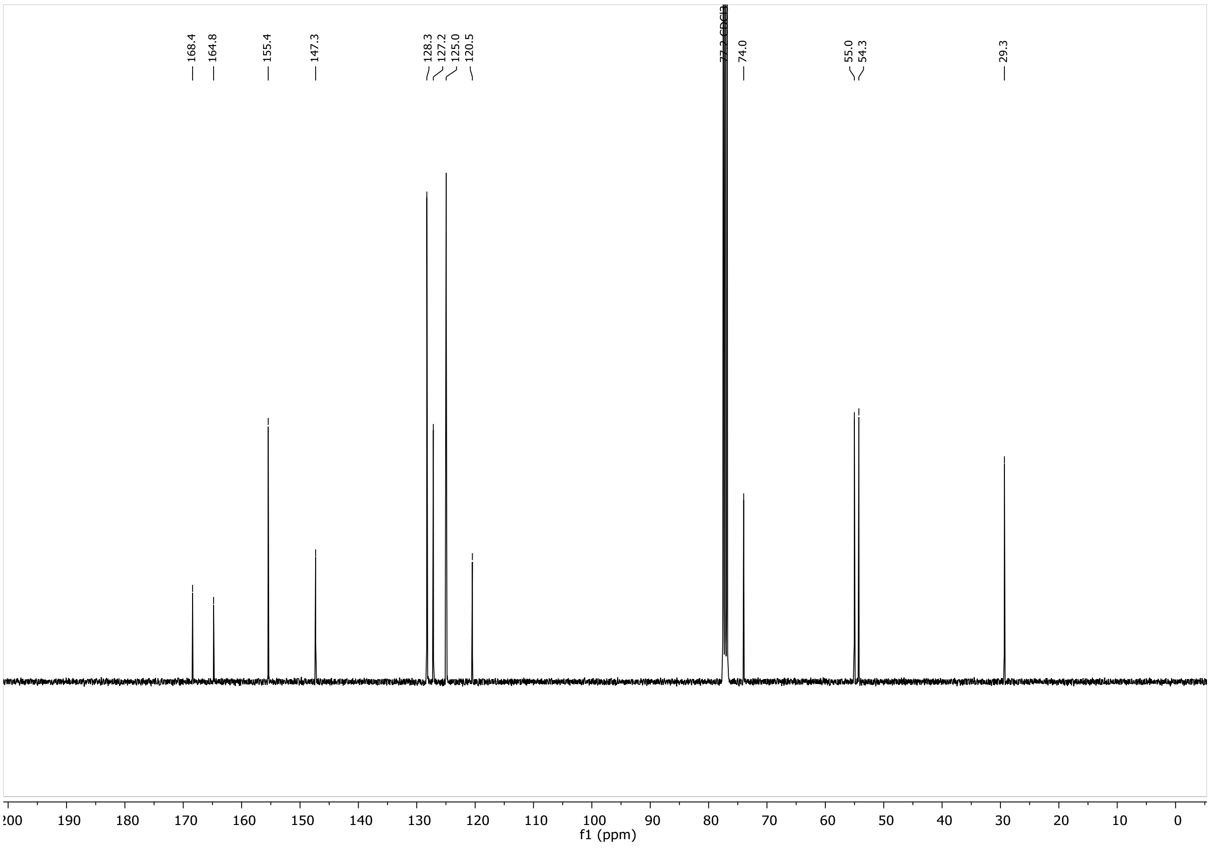

2,4-Dimethoxy-5-(pyridin-2-ylthio)pyrimidine (9cc)

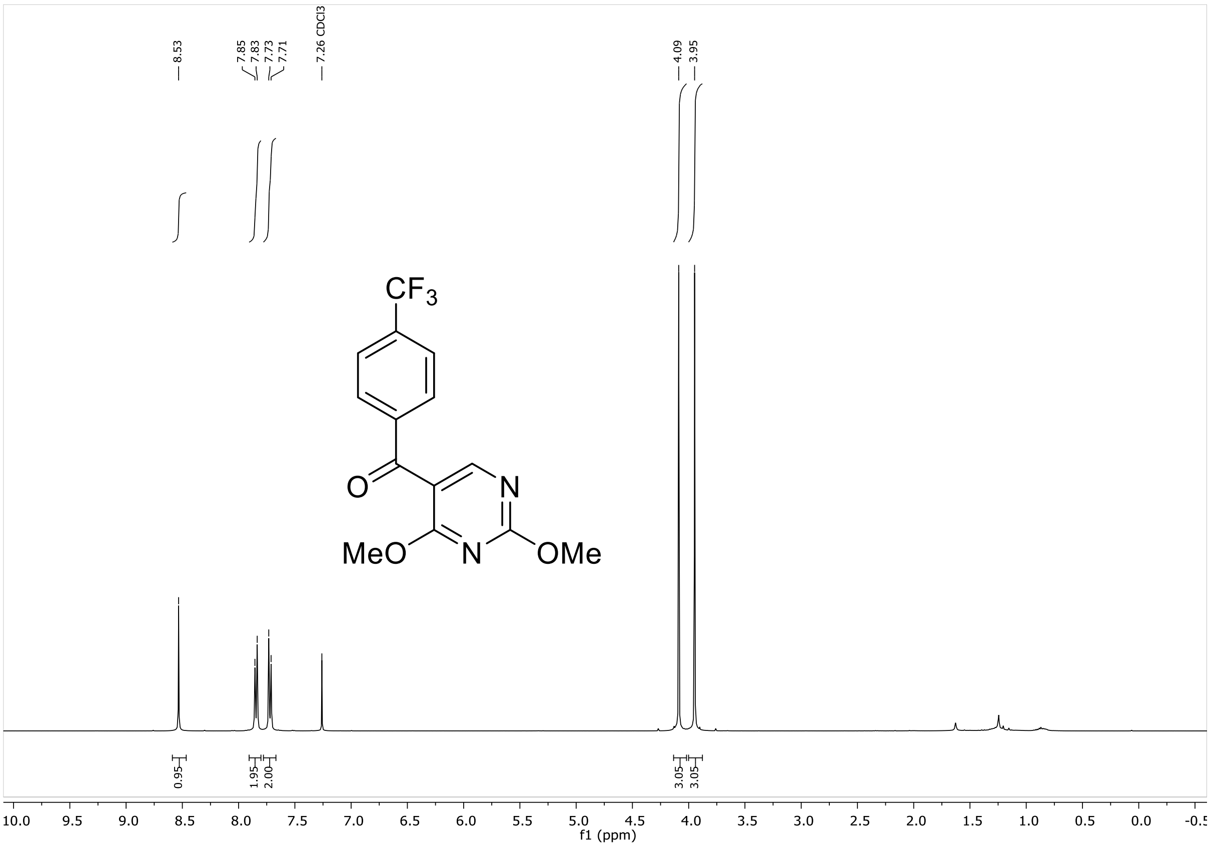

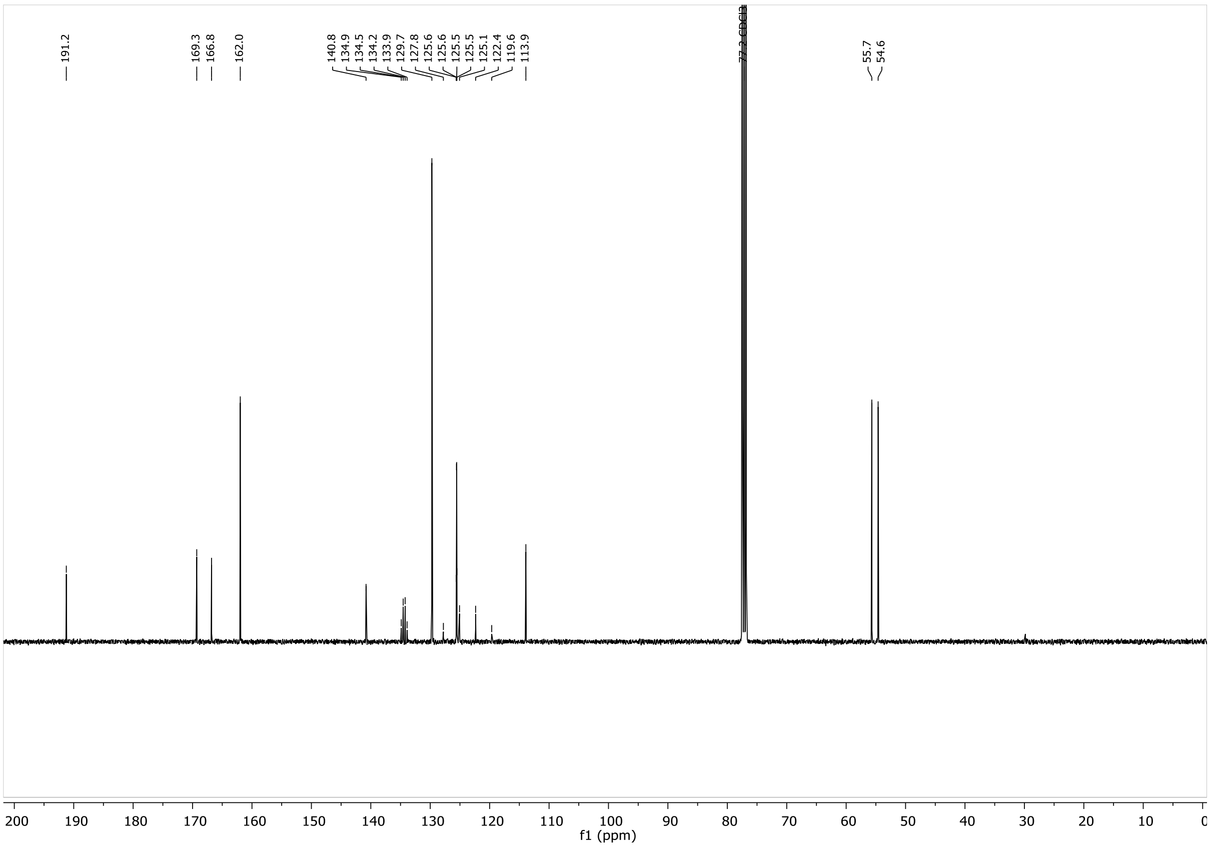

2,4-Dimethoxy-5-(pyridin-2-ylthio)pyrimidine (9cl)

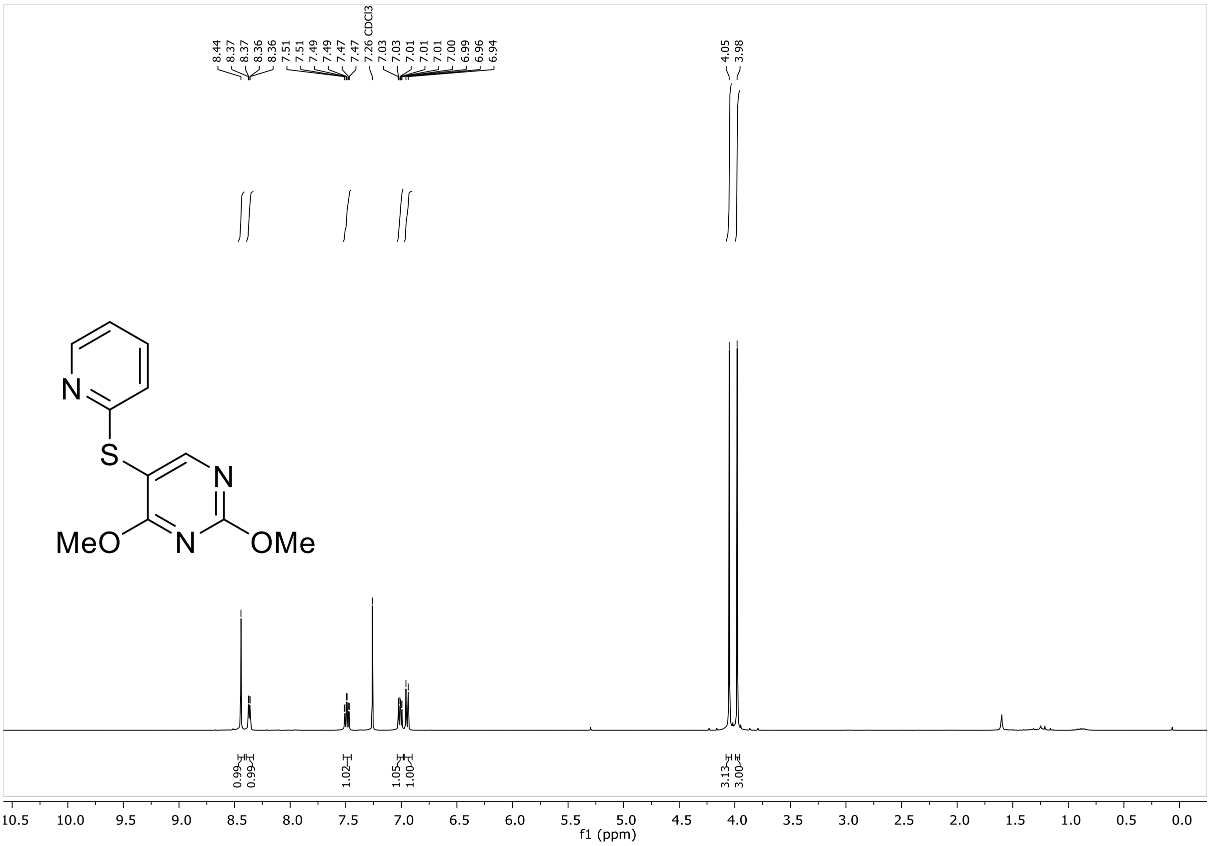

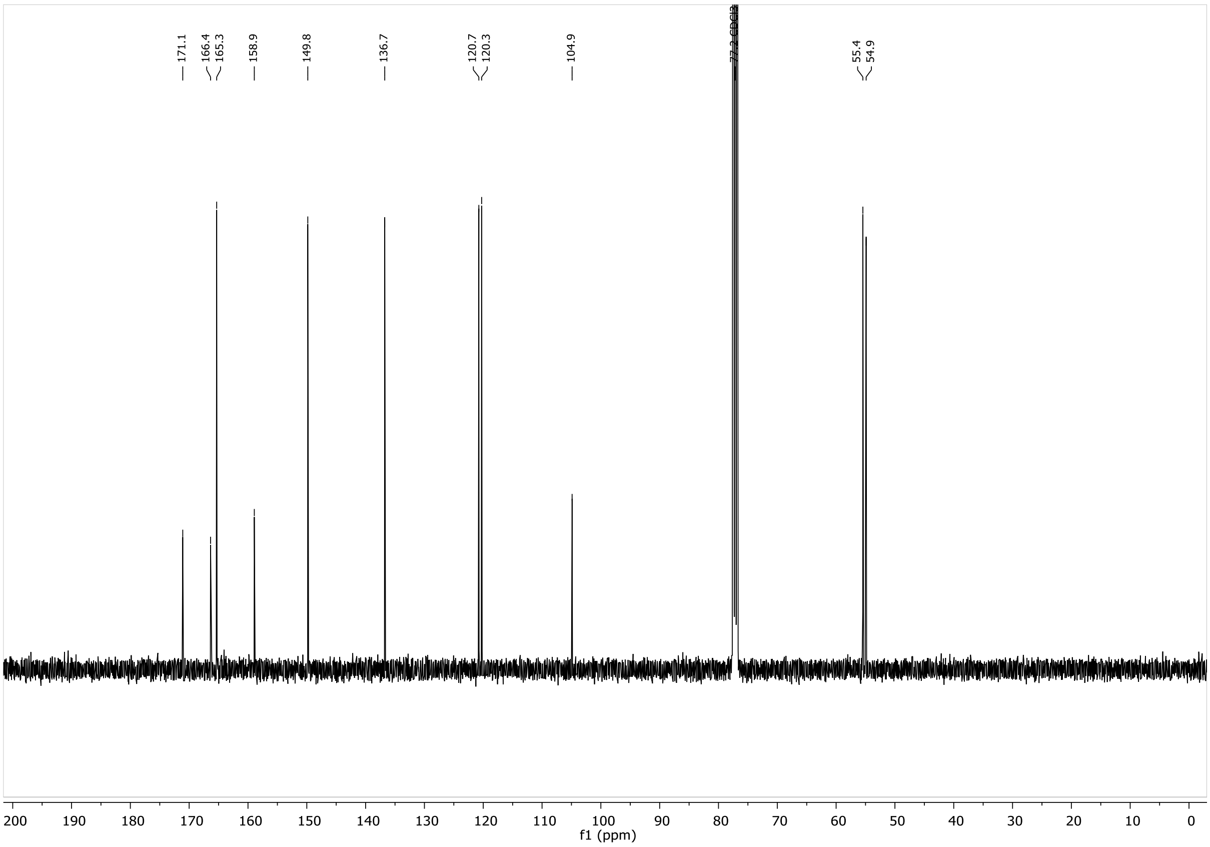

Cyclohexyl(phenyl)(thiazol-2-yl)methanol (9dj)

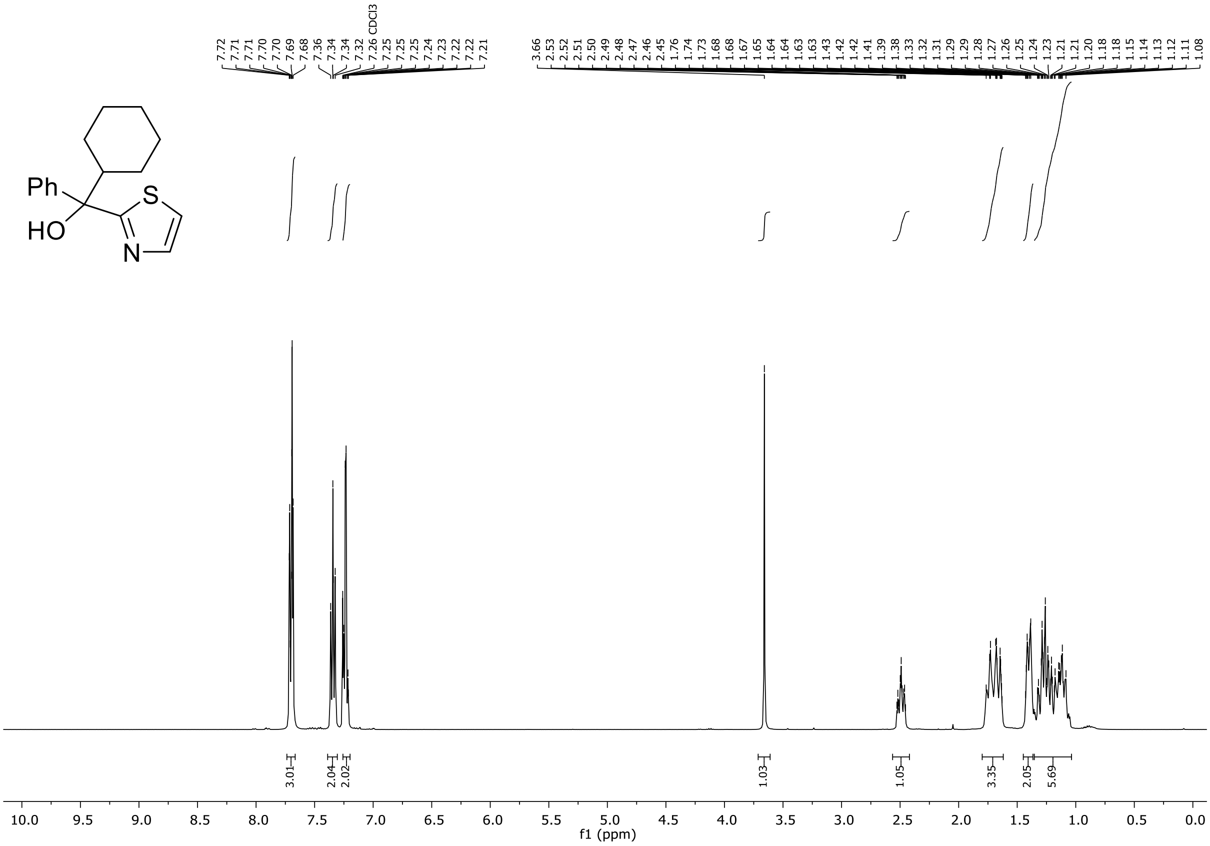

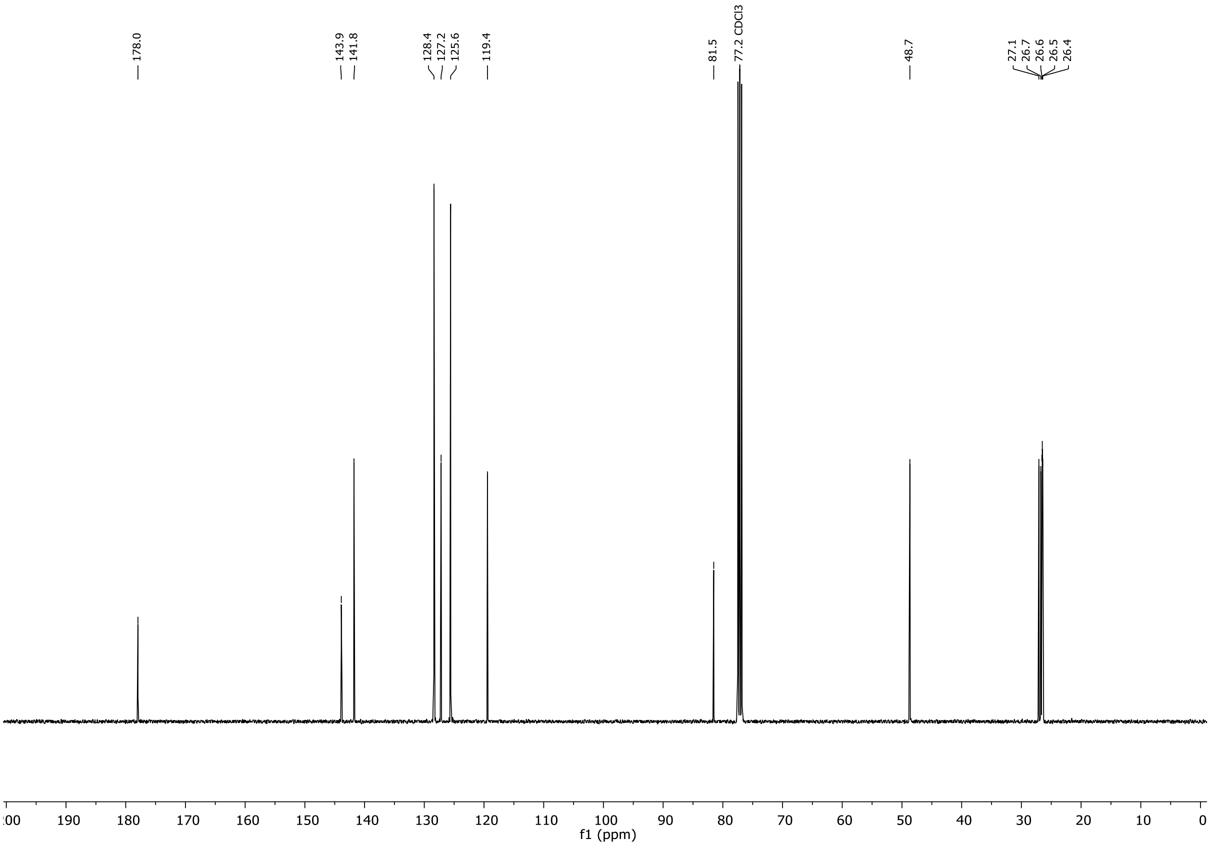

(*E*)-2-(Thiazol-2-yl)-4-(2,6,6-trimethylcyclohex-2-en-1-yl)but-3-en-2-ol (9do)

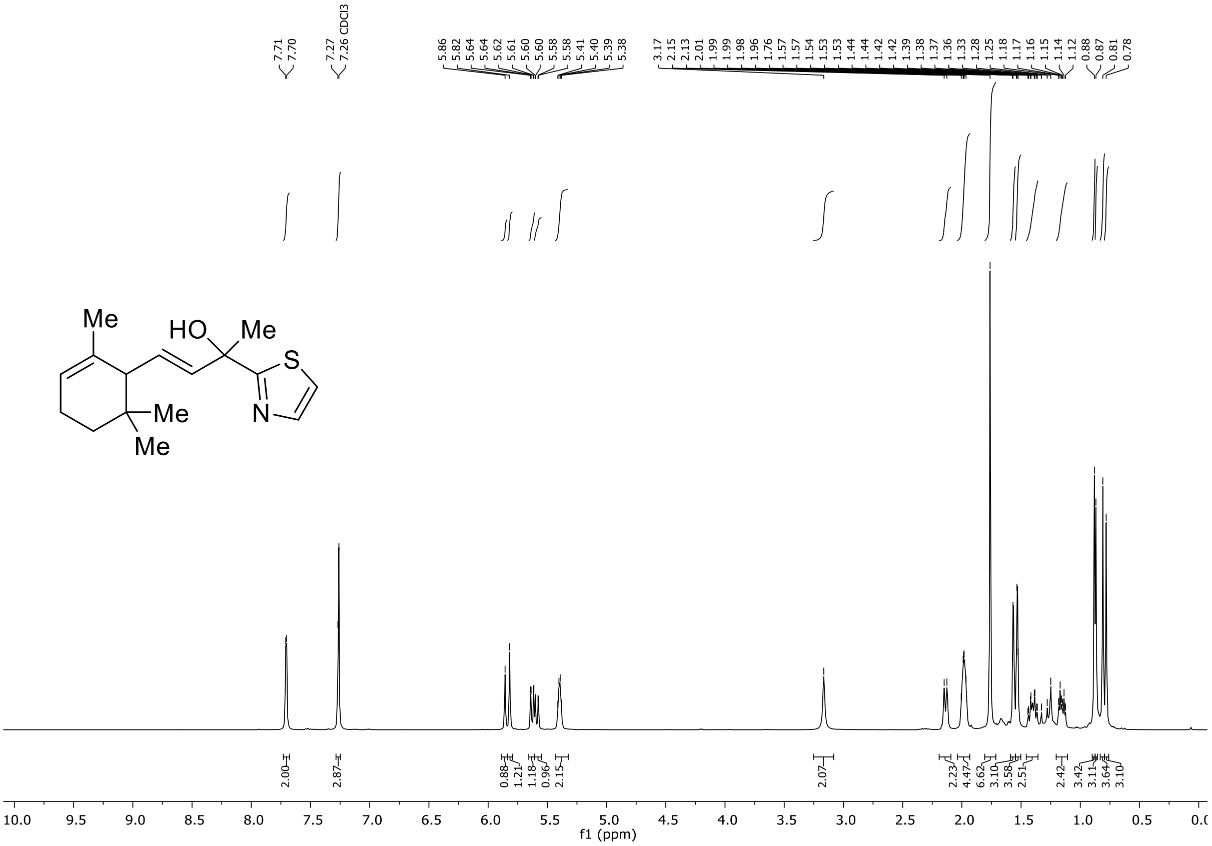

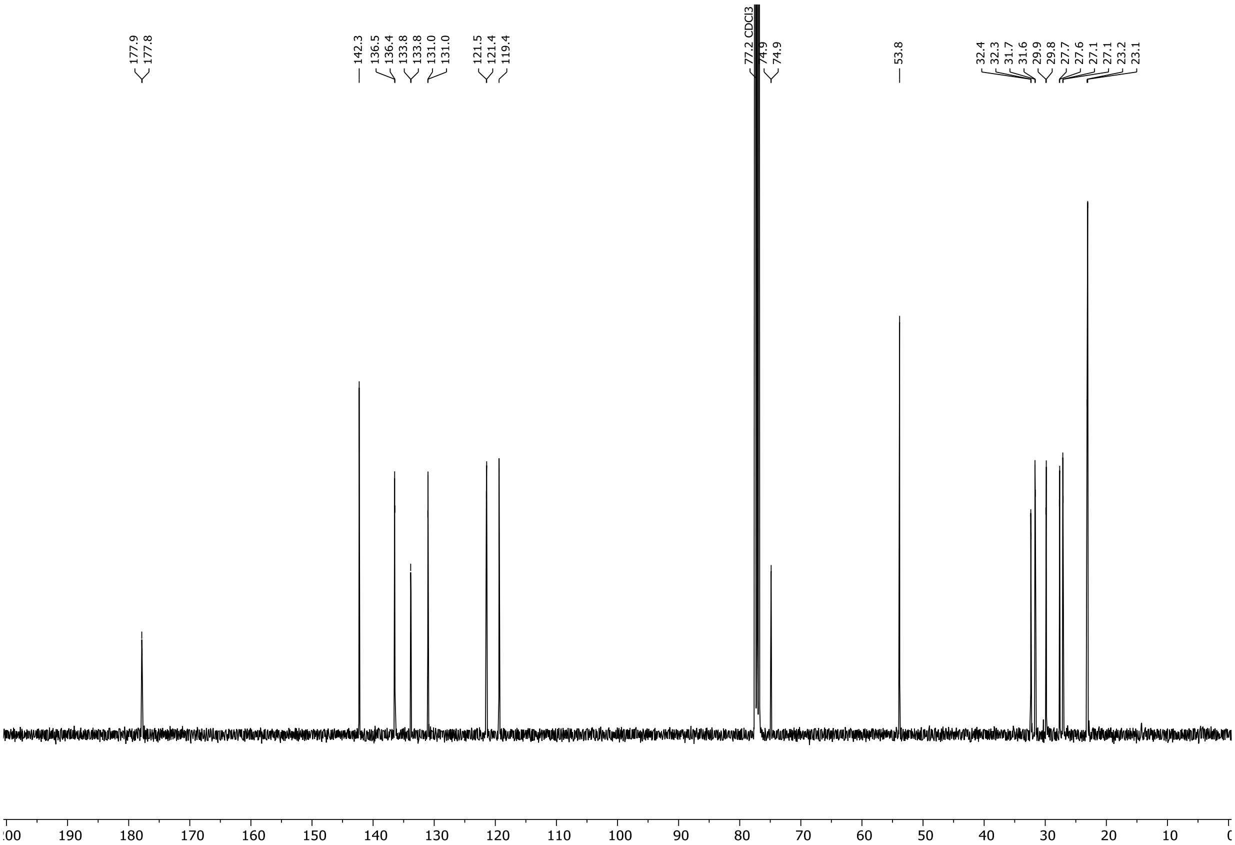

Benzo[*b*]thiophen-2-yl(4-chlorophenyl)(cyclopropyl)methanol (12am)

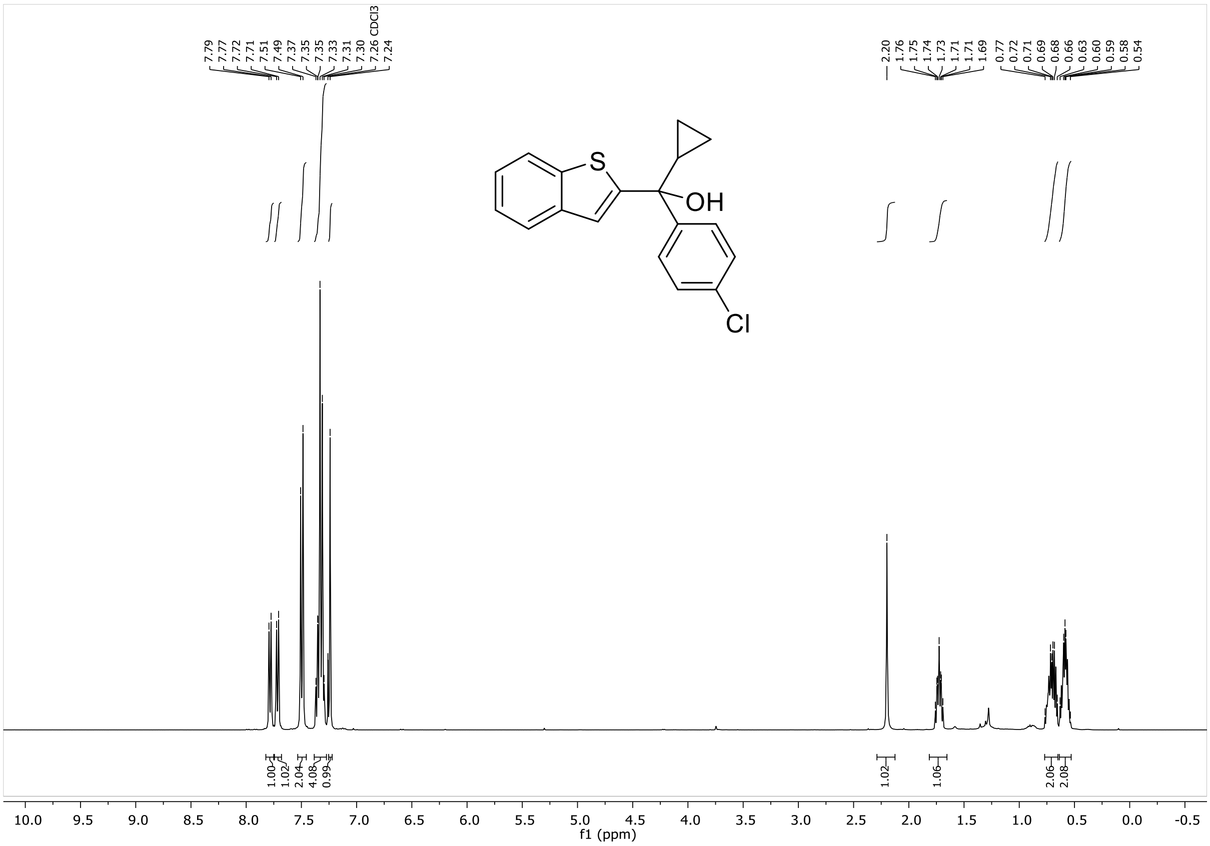

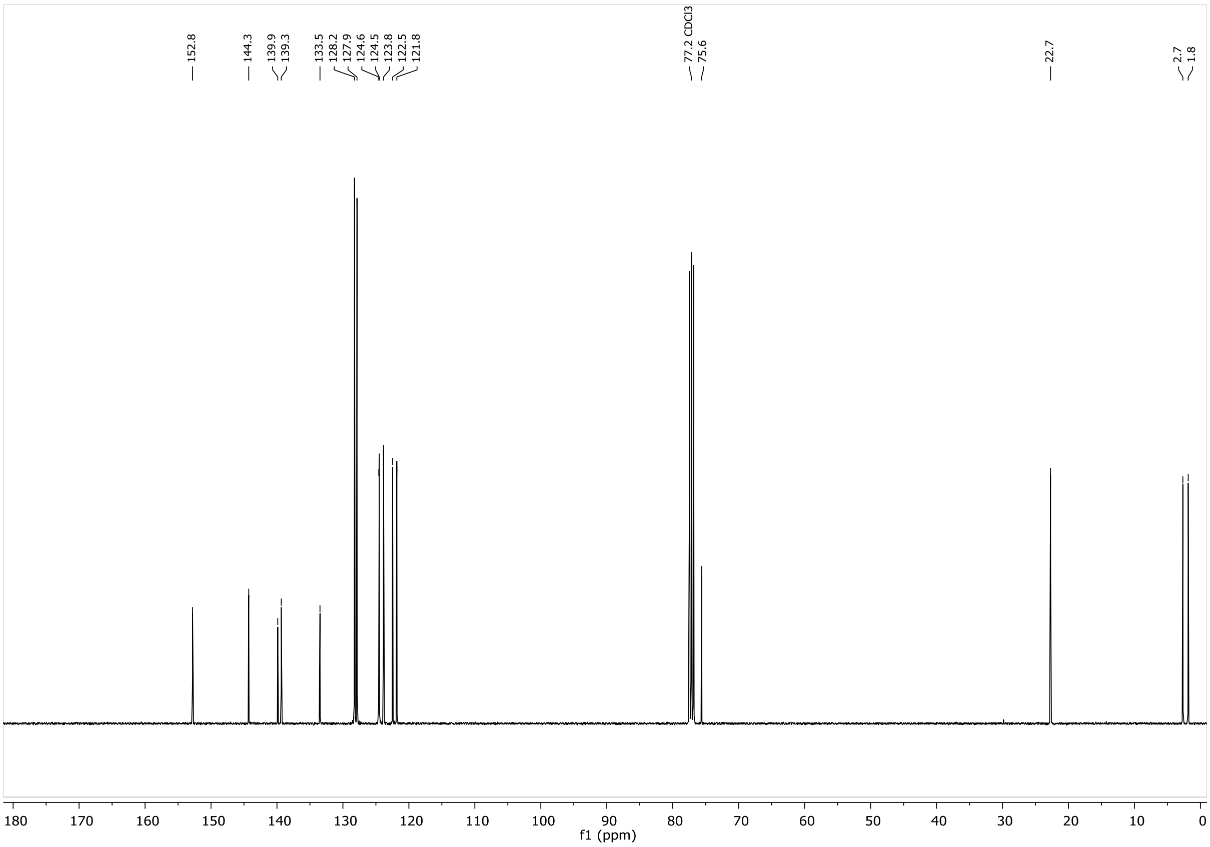

Benzo[*b*]thiophen-2-yl(4-(trifluoromethyl)phenyl)methanone (12ac)

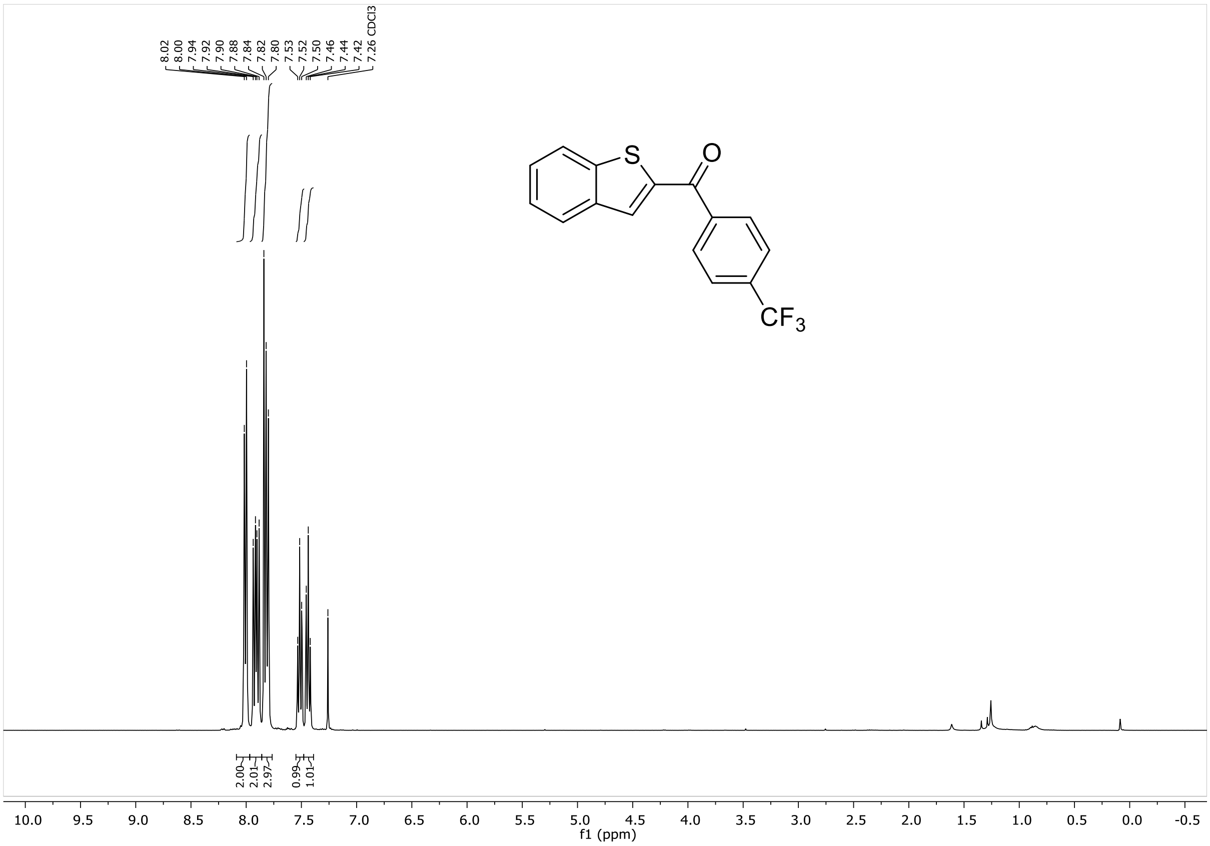

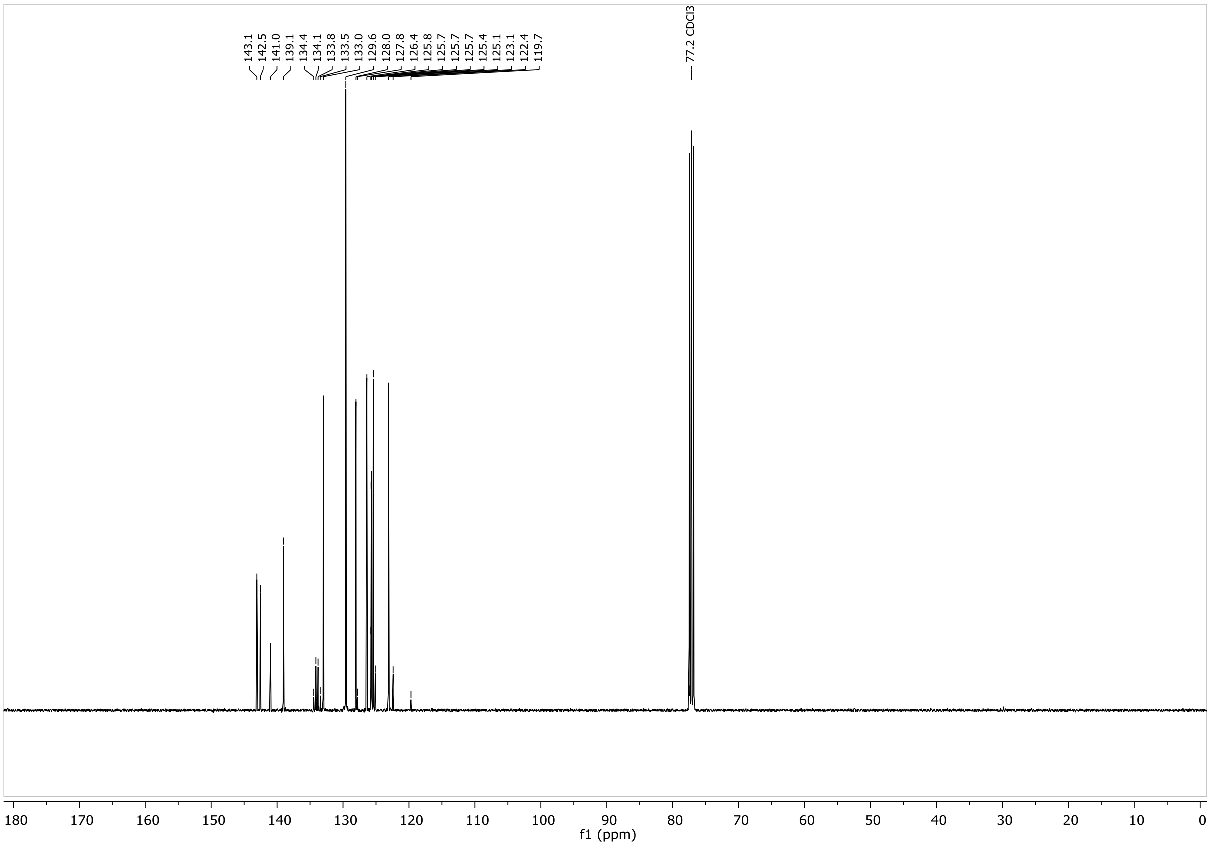

Benzo[*b*]thiophen-2-yl(2,6-dichlorophenyl)methanol (12ag)

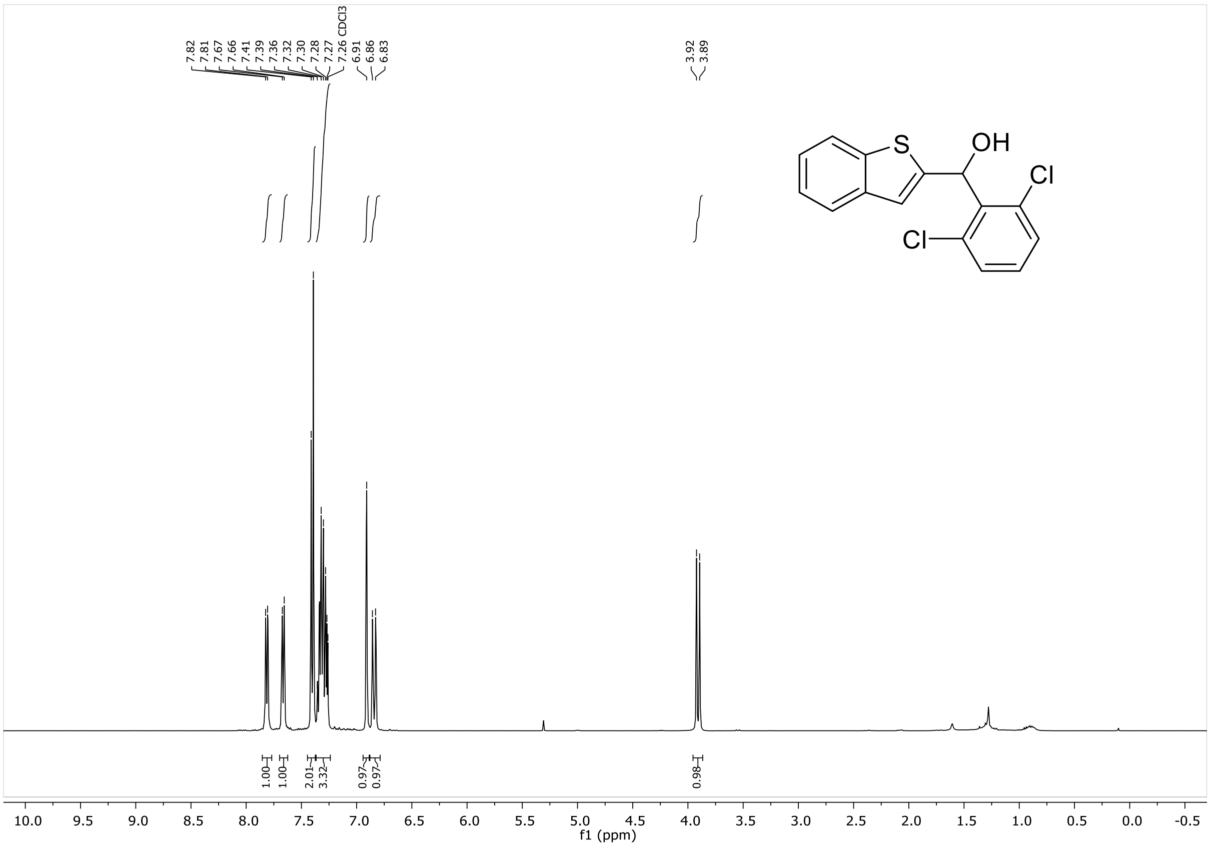

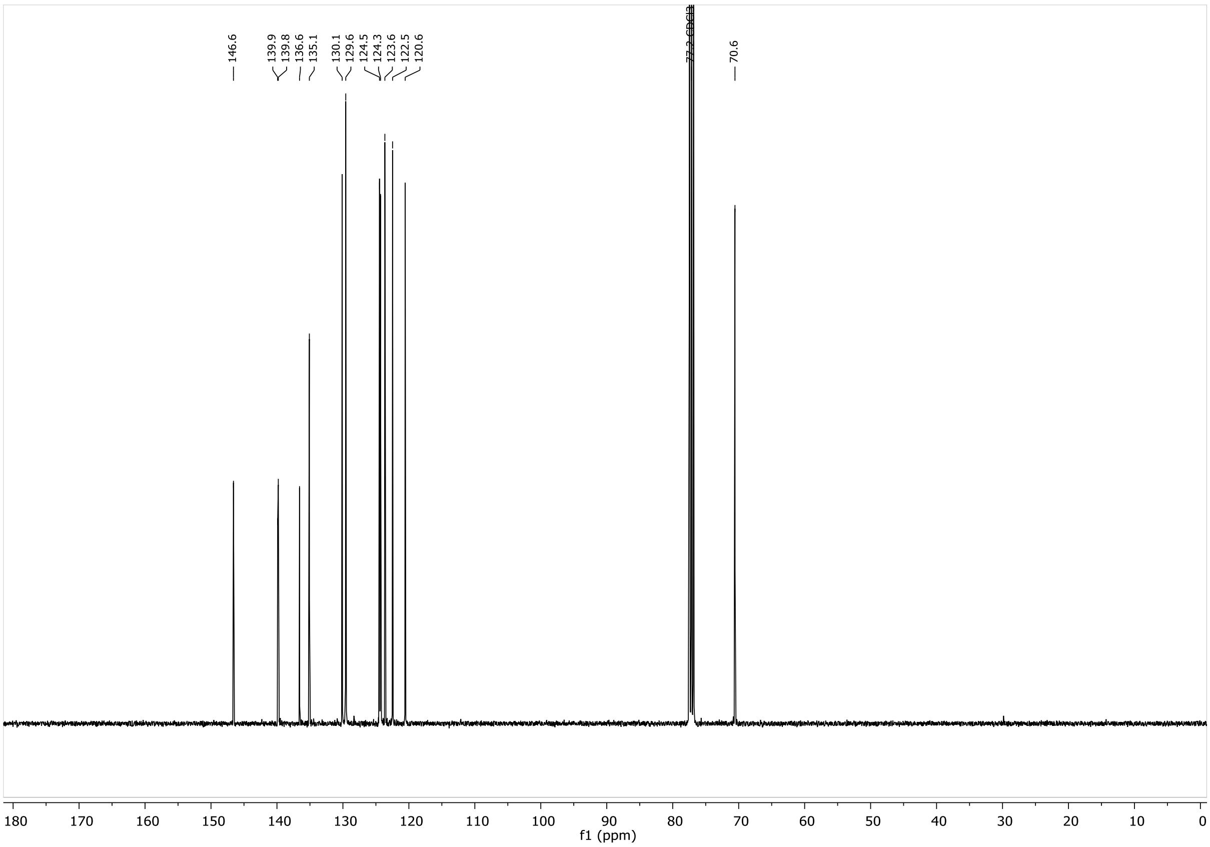

2-((1-Butyl-1*H*-imidazol-2-yl)thio)pyridine (12bl)

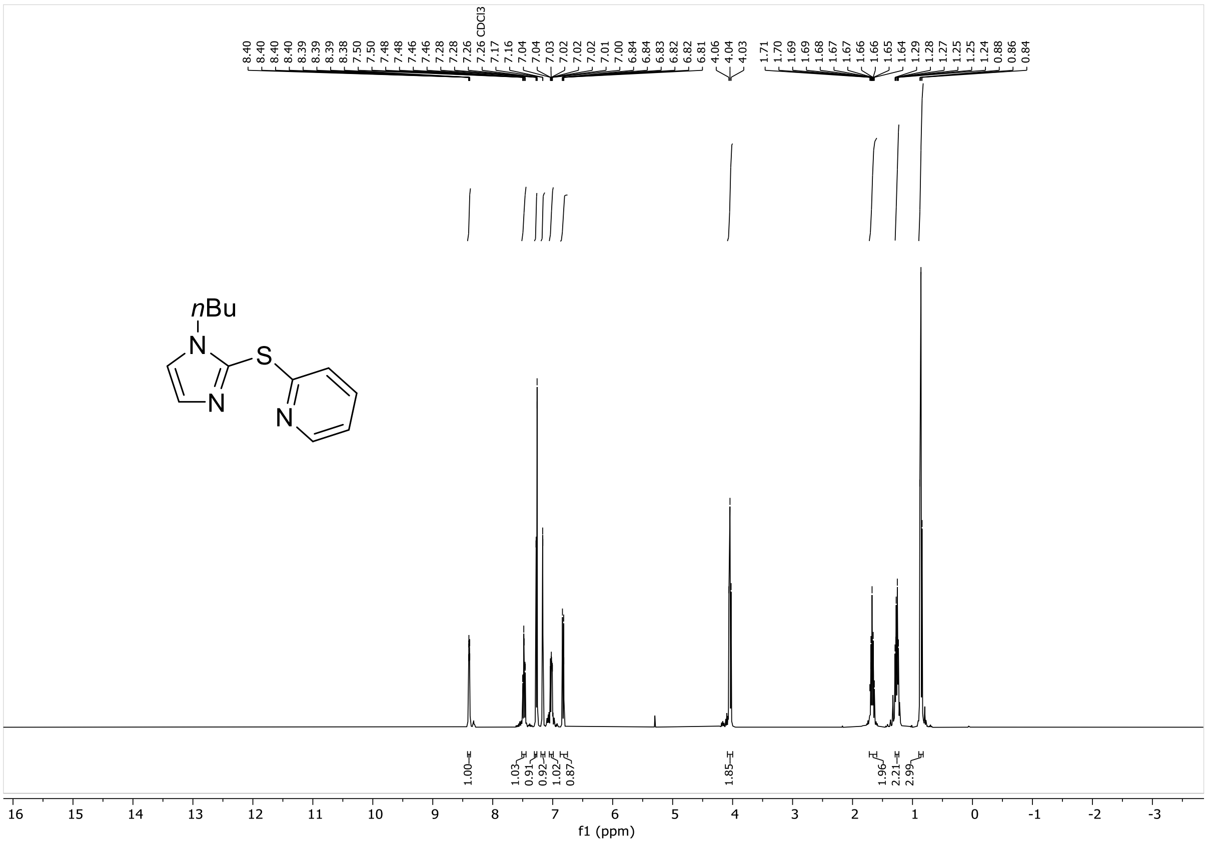

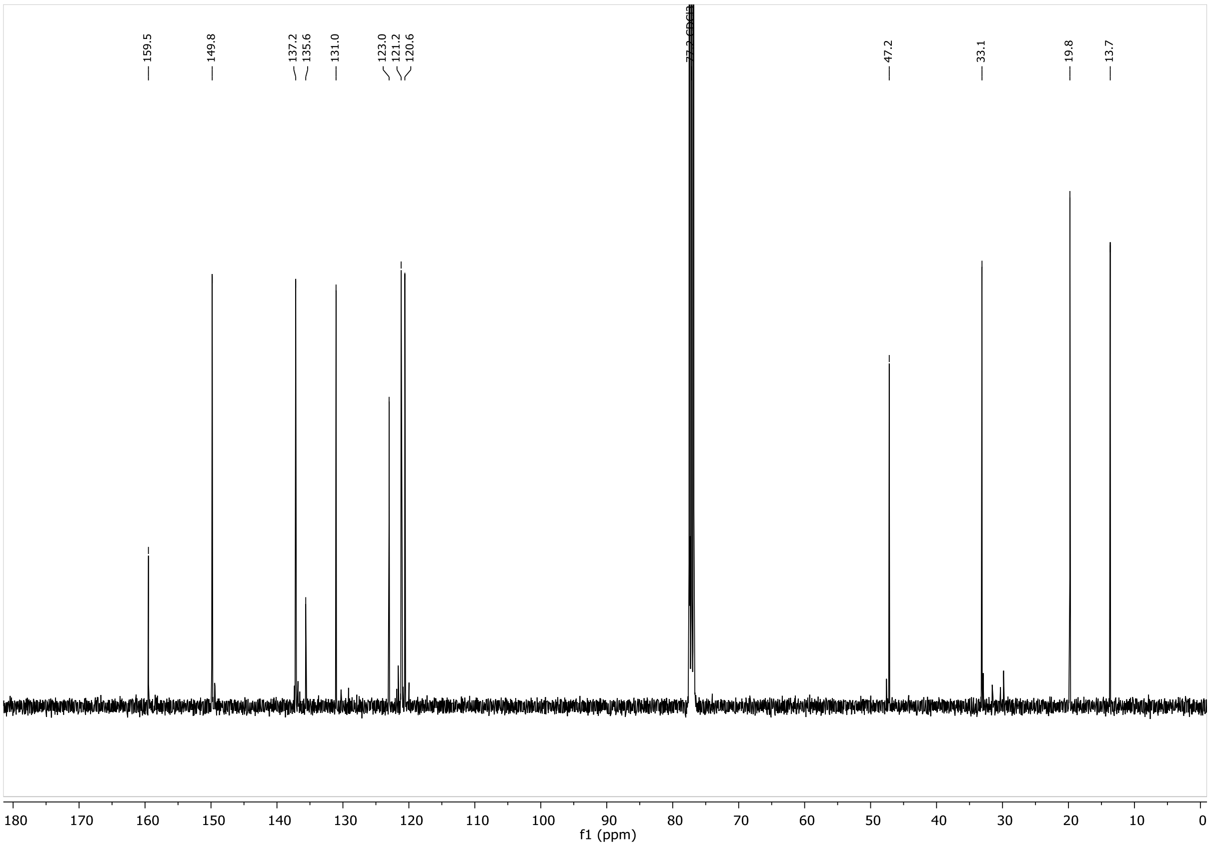

N-((1-Butyl-1*H*-imidazol-2-yl)(phenyl)methyl)aniline (12be)

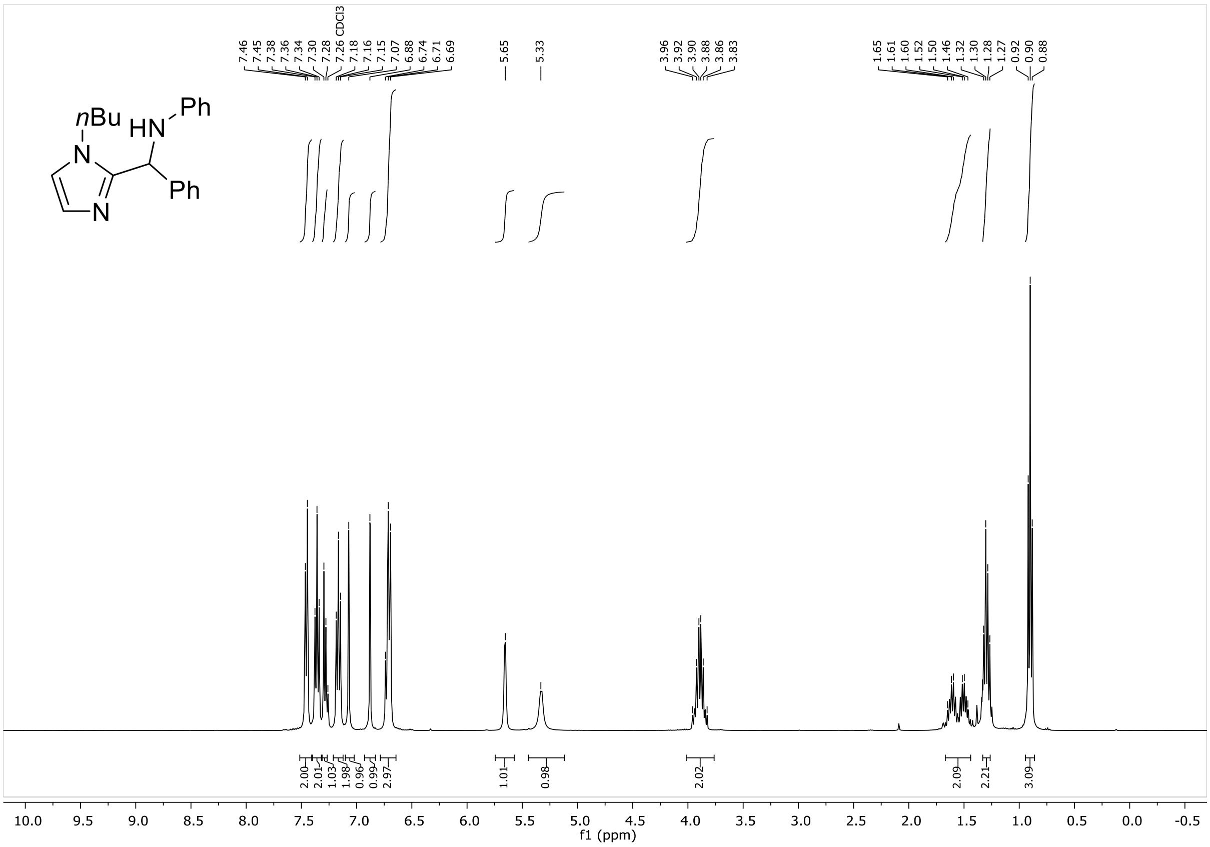

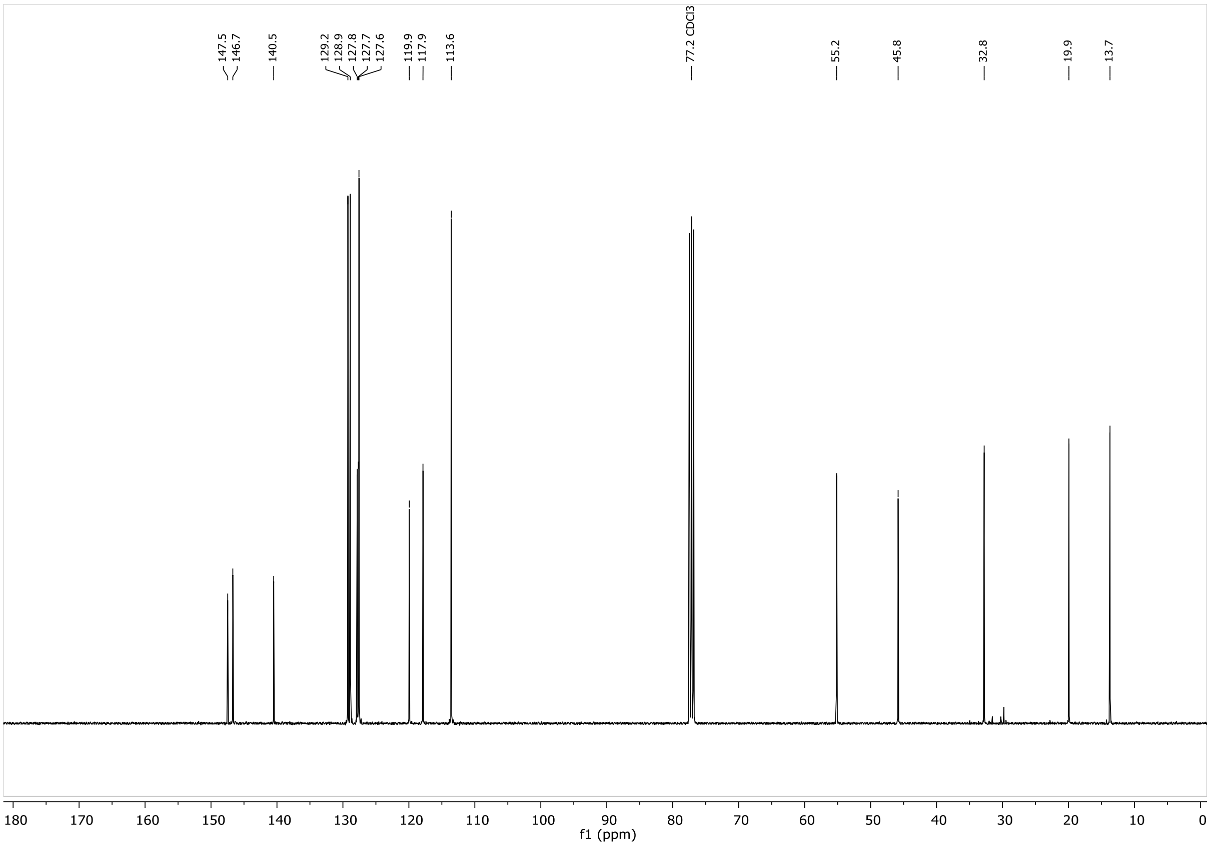

2-(1-butyl-1H-imidazol-2-yl)adamantan-2-ol (12bf)

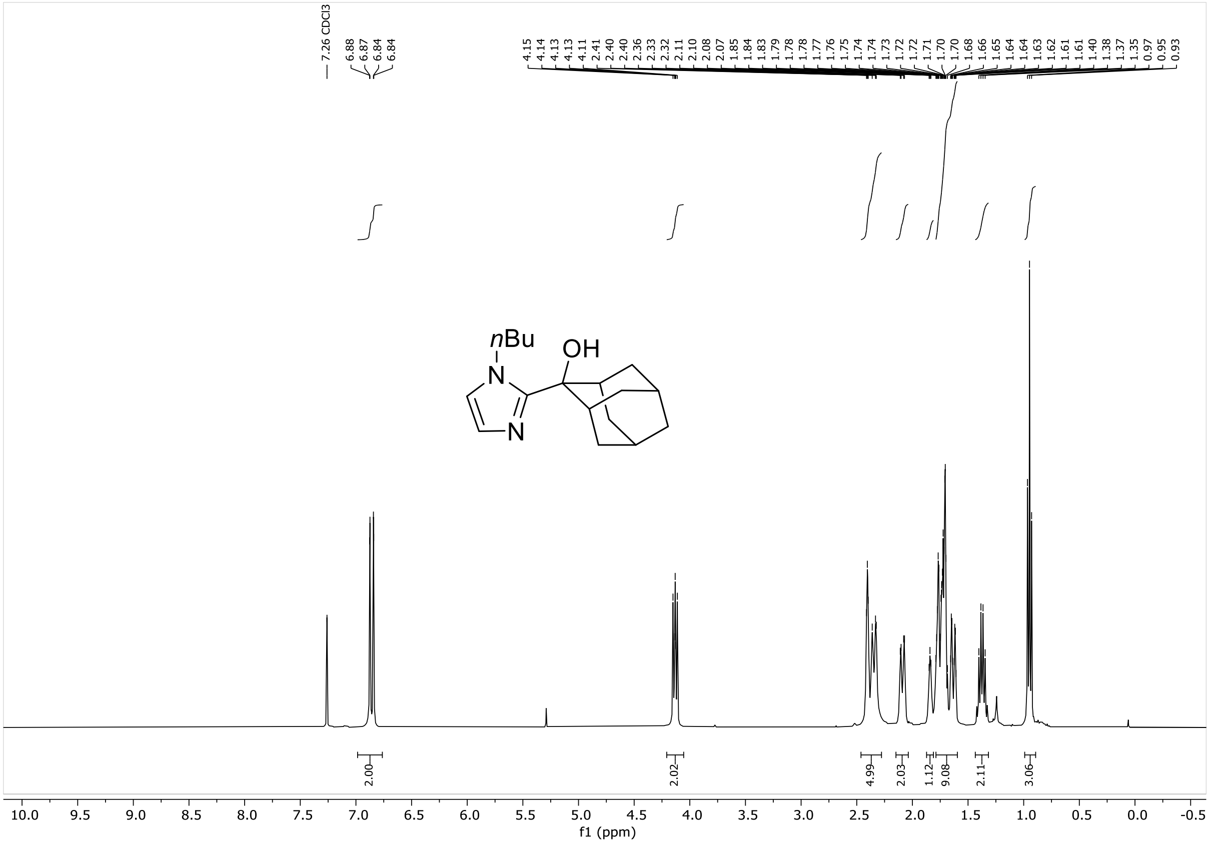

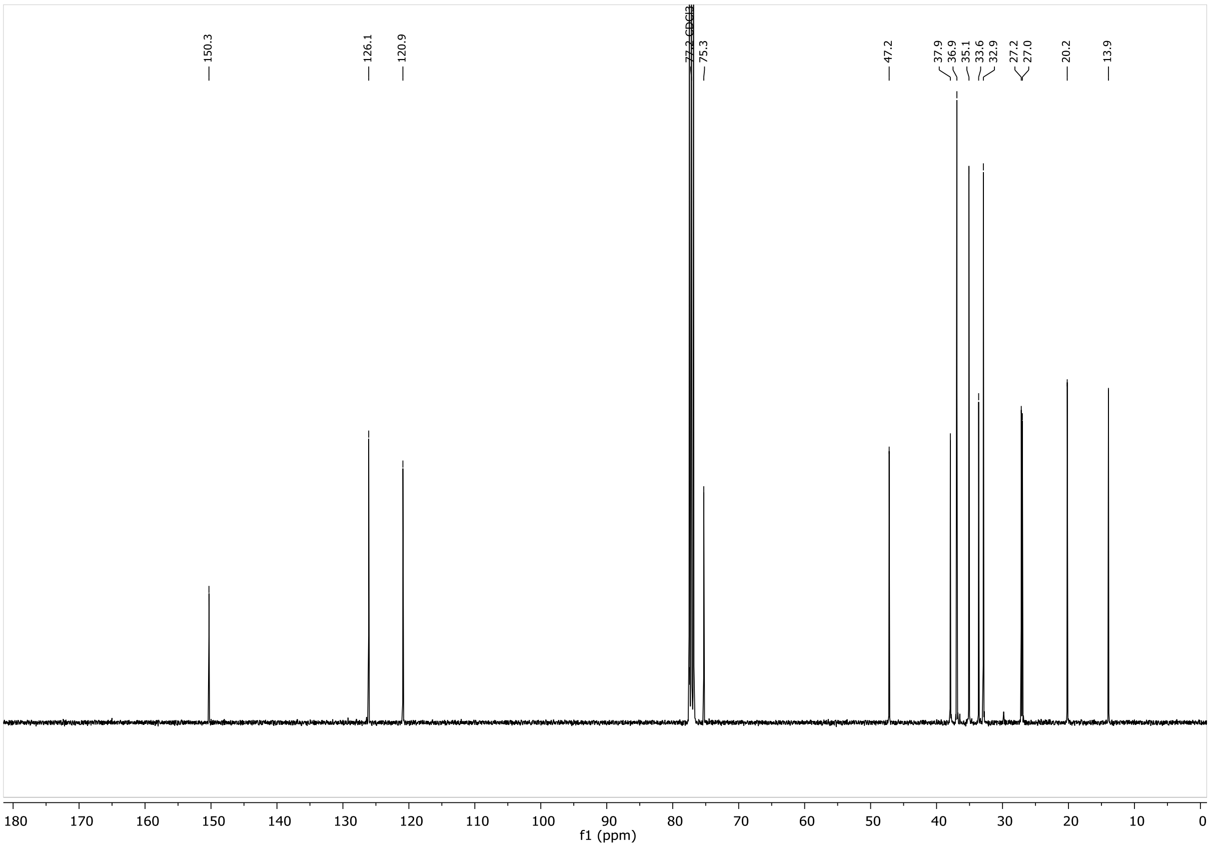

(4-Chlorophenyl)(cyclopropyl)(2,6-dimethoxyphenyl)methanol (12cm)

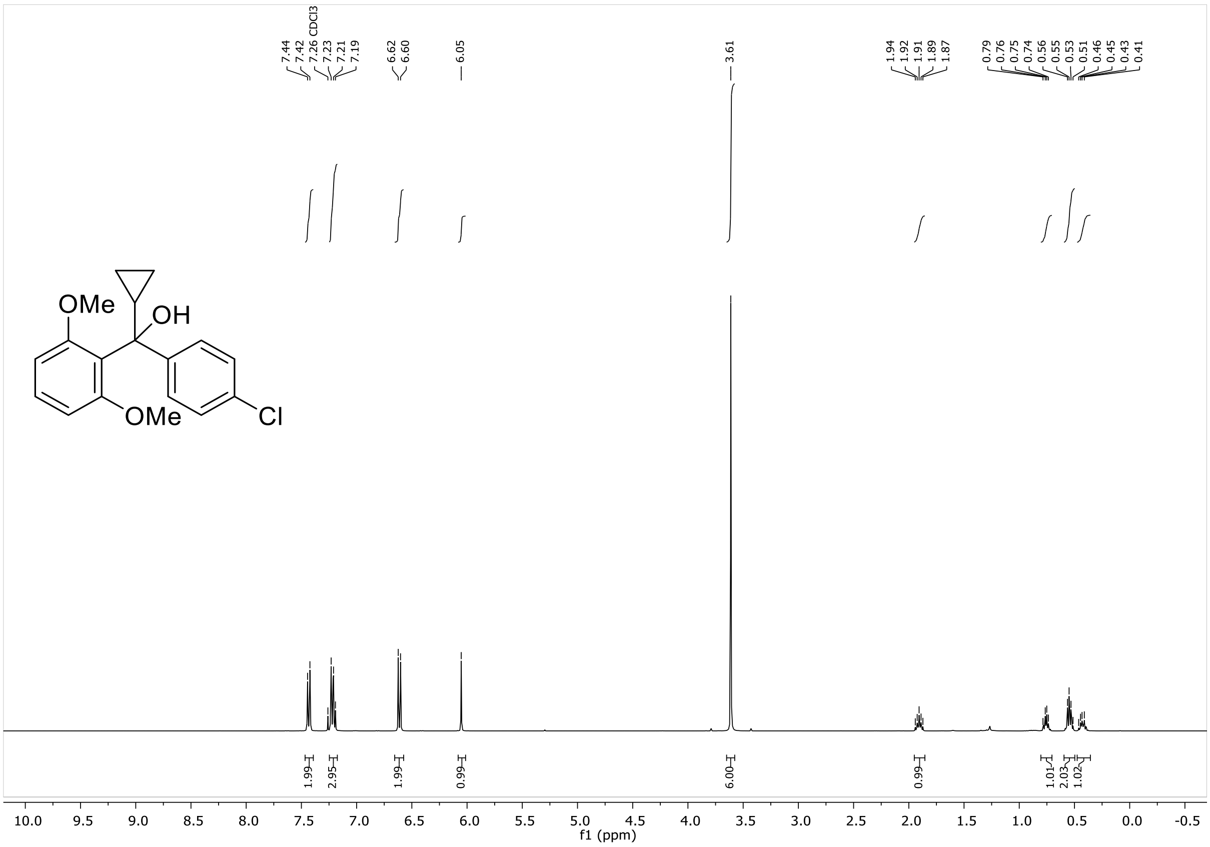

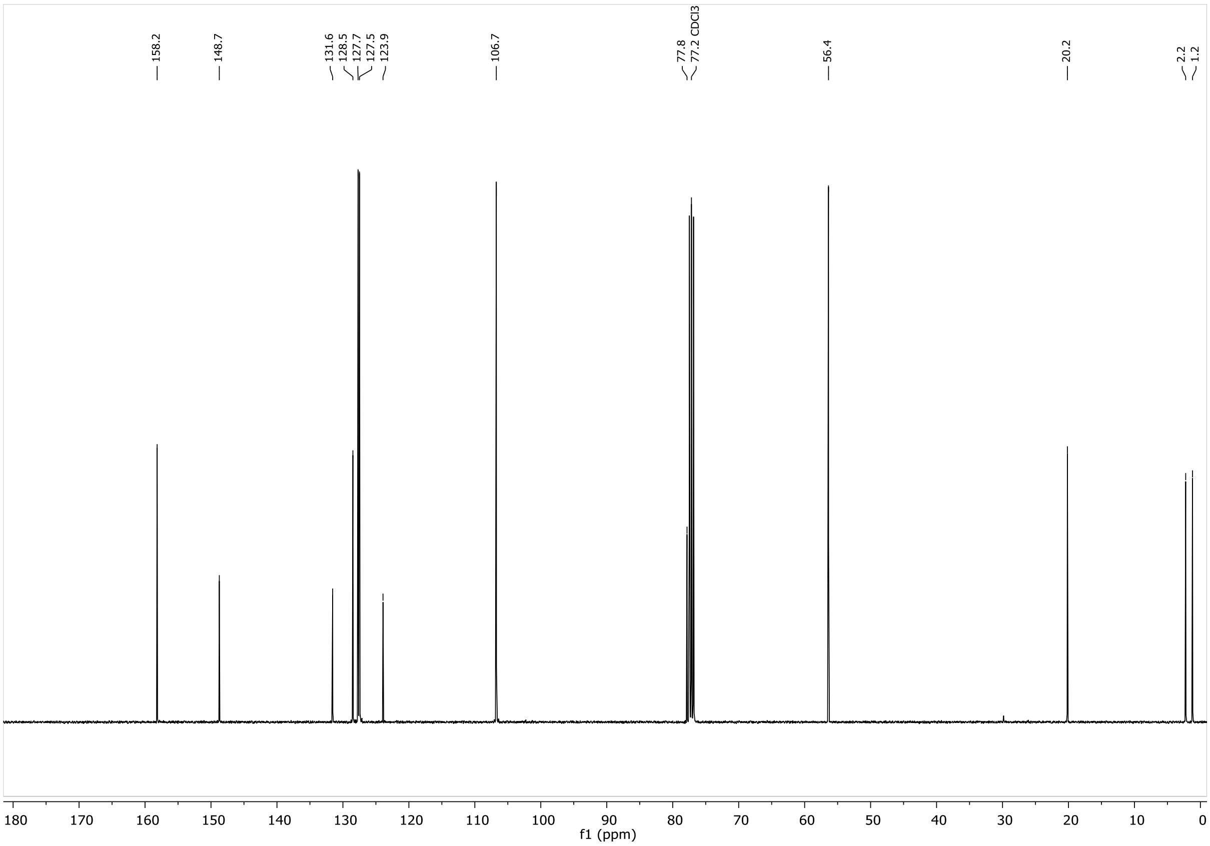

Butyl(2,6-dimethoxyphenyl)sulfane (12cp)

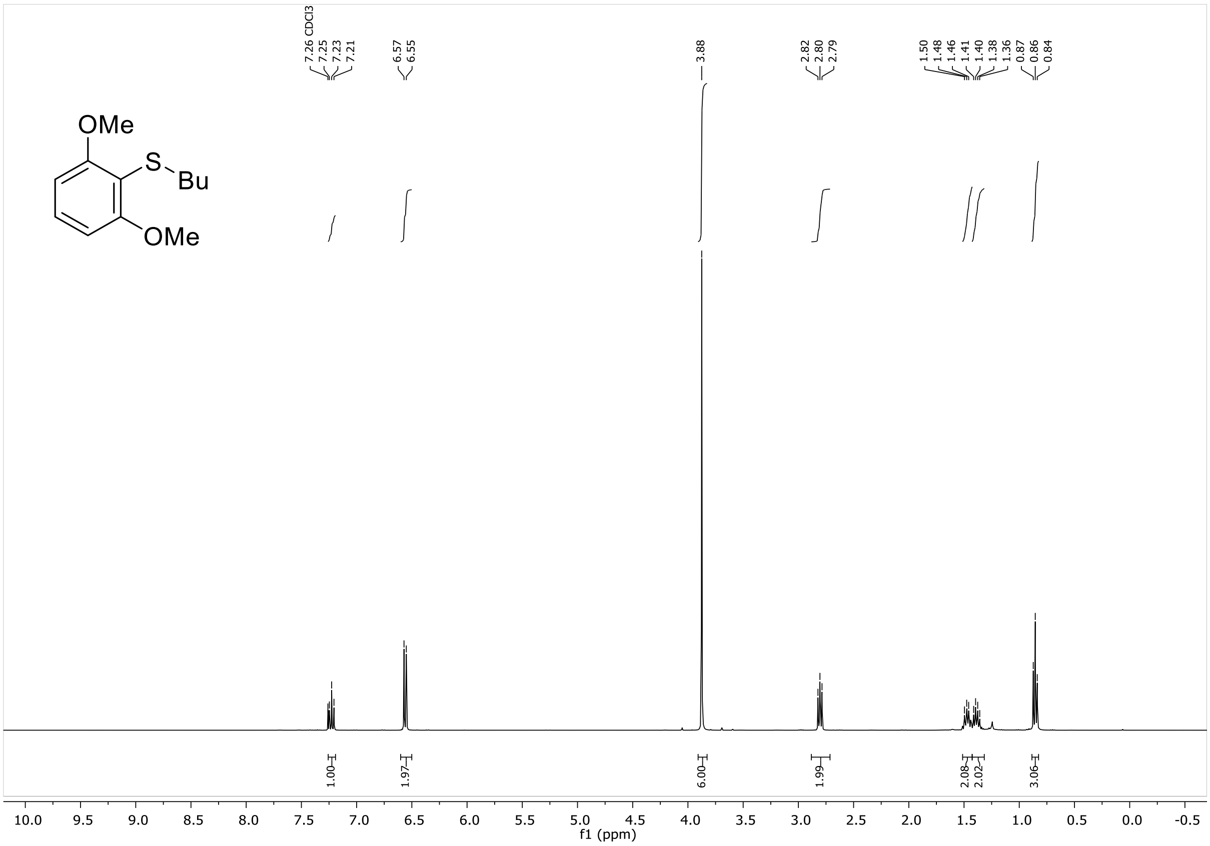

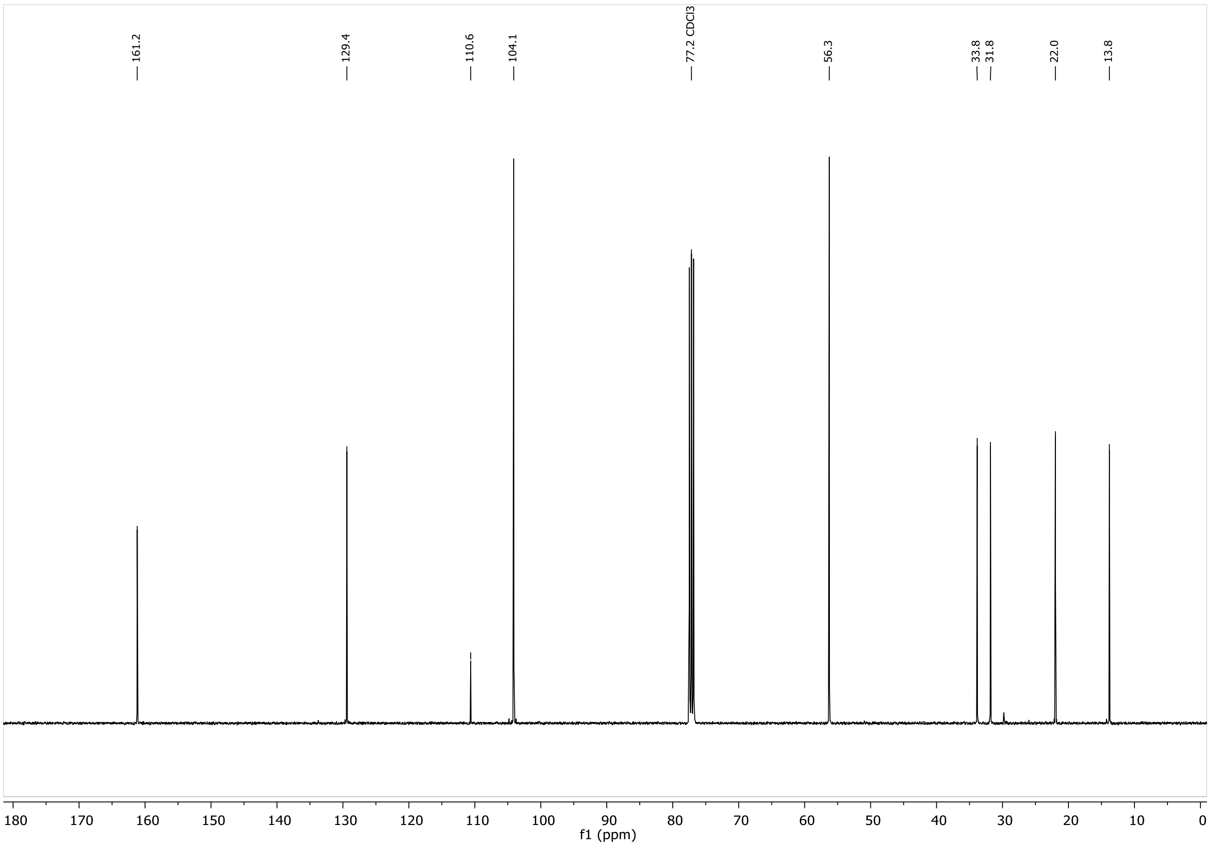

2-Dodecyl-1,3-dimethoxybenzene (12cq)

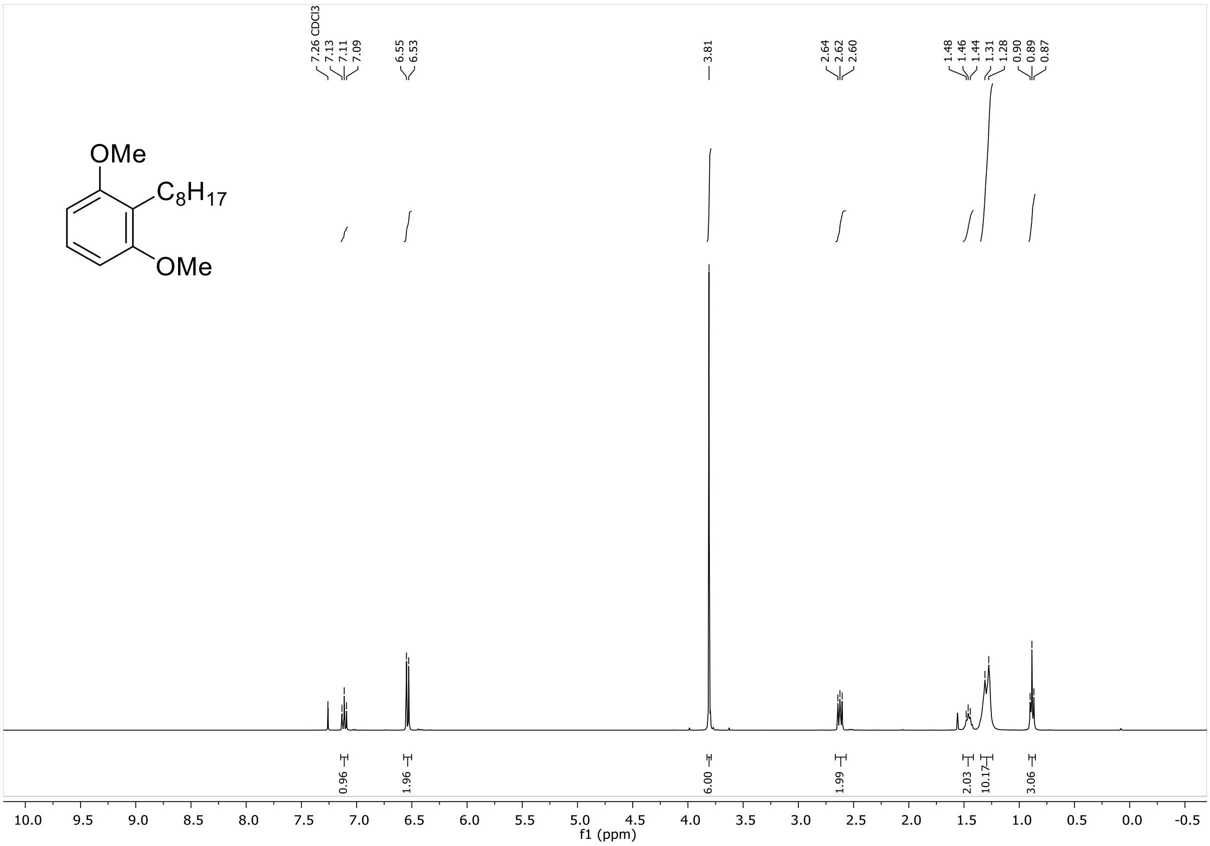

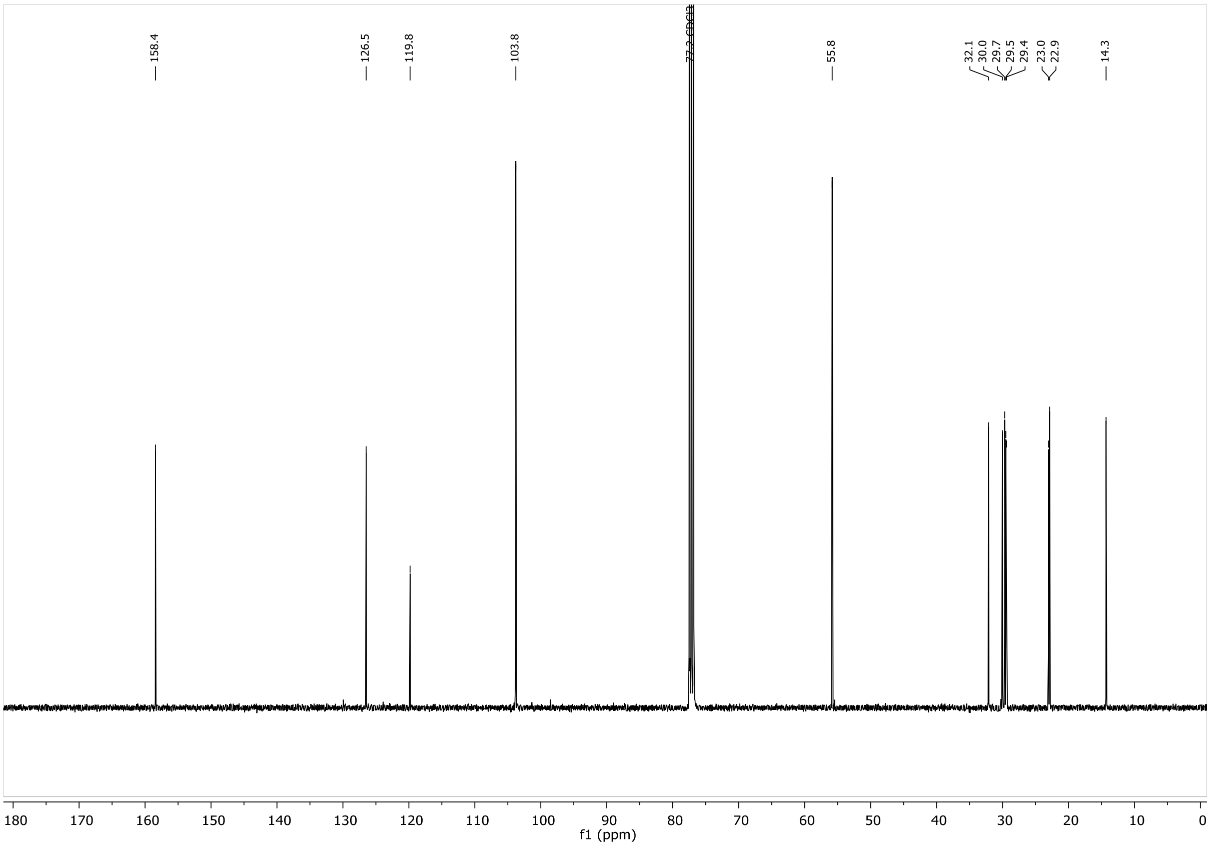

Supplement: Supplementary file 1 — Supplementary [file ANIE-60-14296-s001.pdf]
